# Dehydropolymerisation of Methylamine Borane and an *N*‐Substituted Primary Amine Borane Using a PNP Fe Catalyst

**DOI:** 10.1002/chem.202000809

**Published:** 2020-05-28

**Authors:** Felix Anke, Susanne Boye, Anke Spannenberg, Albena Lederer, Detlef Heller, Torsten Beweries

**Affiliations:** ^1^ Leibniz-Institut für Katalyse e.V. Albert-Einstein-Str. 29a 18059 Rostock Germany; ^2^ Leibniz-Institut für Polymerforschung Dresden Hohe Str. 6 01069 Dresden Germany; ^3^ Technische Universität Dresden 01062 Dresden Germany

**Keywords:** B−N polymers, dehydrogenation, iron, polymerisation, SEC analysis

## Abstract

Dehydropolymerisation of methylamine borane (H_3_B⋅NMeH_2_) using the well‐known iron amido complex [(PNP)Fe(H)(CO)] (PNP=N(CH_2_CH_2_P*i*Pr_2_)_2_) (**1**) gives poly(aminoborane)s by a chain‐growth mechanism. In toluene, rapid dehydrogenation of H_3_B⋅NMeH_2_ following first‐order behaviour as a limiting case of a more general underlying Michaelis–Menten kinetics is observed, forming aminoborane H_2_B=NMeH, which selectively couples to give high‐molecular‐weight poly(aminoborane)s (H_2_BNMeH)_*n*_ and only traces of borazine (HBNMe)_3_ by depolymerisation after full conversion. Based on a series of comparative experiments using structurally related Fe catalysts and dimethylamine borane (H_3_B⋅NMe_2_H) polymer formation is proposed to occur by nucleophilic chain growth as reported earlier computationally and experimentally. A silyl functionalised primary borane H_3_B⋅N(CH_2_SiMe_3_)H_2_ was studied in homo‐ and co‐dehydropolymerisation reactions to give the first examples for Si containing poly(aminoborane)s.

## Graphical abstract

**Getting poly(aminoborane)s**: The well‐known Fe amido complex [(PNP)Fe(H)(CO)] (PNP=N(CH_2_CH_2_P*i*Pr_2_)_2_) is a potent catalyst for the selective formation of poly(aminoborane)s from methylamine borane and a SiMe_3_‐substituted analogue. Mechanistic studies support a chain‐growth mechanism with polymer formation by nucleophilic attack at the terminus of a growing B−N chain.

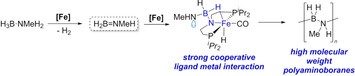

## Introduction

Poly(aminoborane)s represent a comparably new class of inorganic polymers that holds promising properties for a broad range of applications in materials chemistry, for example, as piezoelectric materials or as precursors to boron‐based ceramics.^1^ In most cases, these polymers have been prepared by transition‐metal catalysed dehydropolymerisation of primary amine boranes H_3_B⋅NRH_2_,^2^ however, lately, also metal‐free routes were introduced, including Brönsted acid‐mediated reactions^3^ and BH_2_ transfer to primary amines.^4^ The existing isoelectronic relationships between alkanes and amine boranes H_3_B⋅NRH_2_, olefins and aminoboranes H_2_B=NRH and thus between polyolefins and poly(aminoborane)s [H_2_BNRH]_*n*_ raises a series of questions of how such compounds are activated, coordinated and ultimately formed in the presence of transition metal centres. Control of aminoborane coordination and B−N coupling through a catalyst would allow for the selective and well‐ordered polymerisation, a principle that is known for olefins/polyolefins for decades.^5^ Commonly accepted challenges of amine borane dehydropolymerisation include the identification of general reaction mechanisms, the development of a set of common performance criteria that are deployed for polymer synthesis as well as the elucidation of structure–activity relationships.^6^ The most frequently studied amine boranes for dehydropolymerisation are ammonia borane, H_3_B⋅NH_3_, and methylamine borane, H_3_B⋅NMeH_2_.^7^, ^8^ In the latter case polymers that are soluble in organic solvents can be obtained, which makes mechanistic studies of the reaction much more convenient. Also, reactions with higher substituted dimethylamine borane, H_3_B⋅NMe_2_H, can give valuable insights into the reaction mechanism as in this case, well‐defined, spectroscopically observable and stable intermediates are formed.^9^

The synthesis of *N*‐1d, 4, 10, 11, 12 and *B*‐substituted^13^ poly(aminoborane)s was described in the past, however, to date apart from one example, in which a thiophenyl‐substituted amine borane was used^11^ as the substrate, all reports are limited to alkyl‐substituted compounds. To broaden the scope of this chemistry it would be very interesting to also study other heteroatom‐substituted amine boranes for dehydropolymerisation, the synthesis of some of these substrates was described in the past.^14^

Catalysts for controlled amine borane dehydropolymerisation typically operate in a bifunctional manner, first generating the reactive aminoborane monomer by dehydrogenation of the amine borane, followed by coupling of this intermediate to form oligomers and polymers. Notably, this strongly differs from olefin polymerisation, in which the monomer itself is a stable compound and the catalyst is only involved in coordination and C−C coupling. The stability of aminoborane monomers is strongly dependent on the substitution pattern with larger groups or an increased number of substituents stabilising these species. Methylaminoborane, H_2_B=NMeH, has only been observed at low temperatures,^3^ whereas dimethylaminoborane H_2_B=NMe_2_ can readily be observed spectroscopically at room temperature.^9^ As for the mode of B−N bond formation off‐metal polymerisation, in which the monomer is not coordinated to the metal centre and metal‐centred on‐metal polymerisation were described. Furthermore, for the latter scenario three pathways are suggested, namely (i) amine borane coordination/dehydrogenation/aminoborane insertion at the same metal complex (resembling insertion polymerisation of olefins),^15^ (ii) amine borane coordination/dehydrogenation/aminoborane insertion involving chain transfer through σ complexes^16^ and (iii) dehydrogenation and nucleophilic chain growth through the end of a metal‐coordinated oligomer unit by two different catalyst units (bicatalyst).^17^

**Scheme 1:**
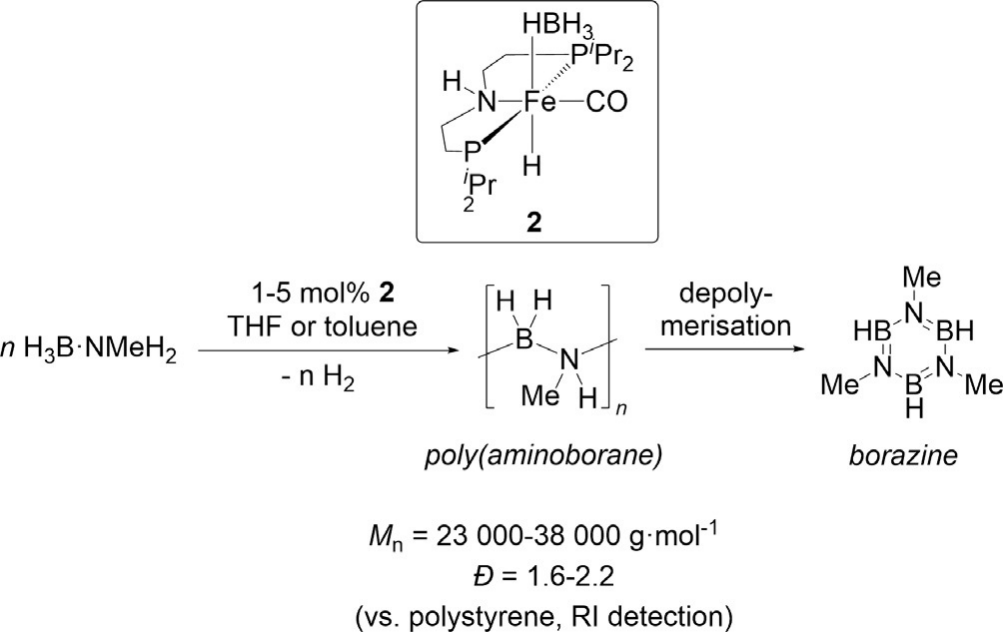
Dehydropolymerisation of H_3_B⋅NMeH_2_ using complex **2**.

Most of the known systems for dehydropolymerisation of H_3_B⋅NMeH_2_ comprise homogeneous late‐transition‐metal catalysts,1d, 10, 15, 18 however, very recently also first examples for highly active early‐transition‐metal complexes were reported.^11^, ^19^ Schneider has presented Ru^II^ pincer systems [(PNP)Ru(H)PMe_3_] and [(PN*H*P)Ru(H)_2_PMe_3_] (PN*H*P=HN(CH_2_CH_2_P*i*Pr_2_)_2_)8d, 8f for H_3_B⋅NH_3_ dehydropolymerisation; the latter was discussed as a bifunctional catalyst that is involved in H_3_B⋅NH_3_ dehydrogenation and B−N coupling. The importance of amine cooperativity for the dehydrogenation step was verified by using the methylated complex [(PN*Me*P)Ru(H)_2_PMe_3_] (PN*Me*P=MeN(CH_2_CH_2_P*i*Pr_2_)_2_), which showed significantly slower turnover. A study on a similar Fe^II^ system [(PNP)Fe(H)CO] (**1**) was later presented by the same authors,8c in which the Fe^II^ dihydrido complex [(PN*H*P)Fe(H)_2_CO] was found the be the resting state relevant to the dehydrogenation step. This is suggested to be followed by Fe‐centred B−N bond formation. Recently, we showed that the related Fe^II^ borate complex [(PN*H*P)Fe(H)(HBH_3_)CO] (**2**) is an excellent precatalyst for the selective dehydropolymerisation of H_3_B⋅NMeH_2_ (Scheme 1), giving borazine (HBNMe)_3_ only at the end of the reaction by depolymerisation of the poly(aminoborane).18d Polymer growth kinetics pointed to the presence of a chain‐growth rather than a step‐growth mechanism. Remarkably, analysis of polymers obtained at different catalyst concentrations suggested that off‐metal polymerisation appears to be dominant.

In this contribution, we present a study of H_3_B⋅NMeH_2_ dehydropolymerisation using the well‐known amido complex **1**.^20^ As an extension of our earlier work, we now give further mechanistic insights into the dehydropolymerisation process that allow for the development of a more general mechanistic picture in reactions with complexes of this type. Also, we present the first examples for Si‐functionalised poly(aminoborane)s that can be readily obtained using the Fe amido complex **1**.

## Results and Discussion

### Dehydropolymerisation with Fe amido complex 1

#### Catalysis

**Scheme 2:**
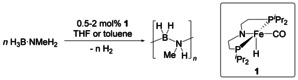
Dehydropolymerisation of H_3_B⋅NMeH_2_ using complex **1**.

Dehydropolymerisation experiments using complex **1** were done at room temperature under isobaric conditions with the reaction vessel connected to an automatic gas buret^21^ that records H_2_ evolution as a product‐proportional concentration. Reactions were done using 0.00167–0.00675 m **1** in THF or toluene (Scheme 2). We found that reactions in THF were much slower and less selective, giving comparably large amounts of borazine (HBNMe)_3_ and other B−N by‐products. Thus, for the discussion of H_3_B⋅NMeH_2_ dehydropolymerisation we focus on results that were obtained from reactions in toluene. Details of experiments that were done in THF can however be found in the Supporting Information.

**Figure 1:**
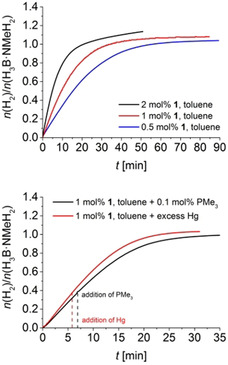
Top: Volumetric curve of H_3_B⋅NMeH_2_ dehydropolymerisation using complex **1** in toluene at *T=*25 °C and [H_3_B⋅NMeH_2_]=0.33 m. Bottom: PMe_3_ and Hg poisoning experiments (*T=*25 °C and [H_3_B⋅NMeH_2_]=0.33 m).

Volumetric curves for reactions in toluene at [H_3_B⋅NMeH_2_]=0.33 m (Figure 1, top) show rapid hydrogen evolution and full H_3_B⋅NMeH_2_ conversion within less than 60 minutes. GC analysis showed that H_2_ was the only gaseous reaction product in all cases.

^31^P NMR monitoring shows that similarly as in earlier work by Schneider8c and our group^22^ the well‐known *trans*‐dihydride [(PN*H*P)Fe(H)_2_CO] is the dominant species (Figure S28). This species is known to readily form from the amido complex **1** in the presence of H_2_.8c, 22, 23 Also, formation of the borate complex **2** occurs, possibly by BH_3_ addition to *trans*‐dihydride [(PN*H*P)Fe(H)_2_CO], as proposed earlier.8c Notably, formation of [(PN*H*P)Fe(H)_2_CO] can also be seen from an instantaneous colour change from purple, typical for **1**, to pale yellow upon addition of H_3_B⋅NMeH_2_. We discount the formation of a heterogeneous catalyst as the active species, as addition of 0.1 mol % PMe_3_ or excess Hg did not affect the kinetic profile of the reaction (Figure 1, bottom).^24^

NMR spectroscopic speciation experiments with equimolar amounts of catalyst **1** and H_3_B⋅NMeH_2_ showed formation of a new set of triplet ^1^H resonances at −14.53 and −15.07 ppm (^2^ *J*_H,P_=58.2 Hz), likely due to *cis*/*trans* isomers of an intermediate hydride species (Figure S54). Along with this a broad signal at −10.37 ppm could indicate the presence of a *μ*‐BH moiety of an aminoborane capped species [(PN${}^{\mathrm{BH}{}_{2}\mathrm{NMeH}}$ P)Fe(H)CO] **1‐H_2_BNMeH**. ^11^B NMR spectra of this new complex show a broad doublet at −10.2 ppm (^1^ *J*_B,H_=115 Hz) that collapses into a singlet upon proton decoupling (Figure S56). Related Ru borametallacycles derived from reactions of [(PNP)Ru(H)PMe_3_] with H_3_B⋅NH_3_ and H_3_B⋅NMeH_2_ were described before by Schneider and showed similar NMR spectra.9b Also, we have presented a BH_3_ adduct in an earlier communication.18d

Kinetic analysis using commonly applied linearisations of the integrated rate law for the determination of *k* values have shown that most of the plots clearly deviate from the expected linearity for an assumed simple first‐order reaction (Figure S17).^25^ This is also reflected in VTNA analysis^26^ of the reaction profiles with variations in [**1**], which are in agreement with a process that is first‐order in the Fe catalyst, but show minor deviations that cannot be neglected (Figure S23). In line with this, volumetric data obtained from reactions in toluene and THF at low catalyst concentrations can be best described using a Michaelis–Menten model (Eq. 1, see Supporting Information for details).
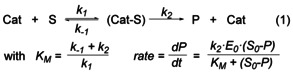



In contrast, volumetric curves of reactions using higher catalyst concentrations could be conveniently fitted using a first‐order model. In this model (Eq. (1), according to the fitted values for *K_M_* and *k* the active catalyst (Cat) and the substrate (S) are in equilibrium with a catalyst‐substrate complex (Cat–S), which is converted into the product (P) and releases the active catalyst (Cat) (Table 1). For a first‐order reaction as the limiting case of Michealis–Menten kinetics, the *K_M_* values should be higher. Or in other words, as 1/*K_M_* represents at least the upper limit of the thermodynamic stability constant, the pre‐equilibrium is far shifted to the left [Eq. (1)]. Saturation kinetics at high substrate concentrations were reported before in H_3_B⋅NMe_2_H dehydrocoupling for a [Rh(Ph‐Xantphos)]^+^ system.^15^ For H_3_B⋅NMeH_2_, similarly as in our case, this zero‐order regime (*v_max_*) could not be reached due to limited solubility of H_3_B⋅NMeH_2_ in toluene. In our case the rate is limited to *v*≈*v_max_*/2 (Table 1, entry 3). For this, *K_M_* should be in the same range as [H_3_B⋅NMeH_2_]_0_, which is in line with the observed values. In previous studies simple first‐order kinetics were proposed for related systems based on linearisations of the respective integrated rate law.8c, 8d, 27 We show that using a Michaelis–Menten‐type approach as a more general kinetic model, which includes often described first‐order behaviour as a limiting case, could help to refine the interpretation of experimental data and explain systematic deviations from ideal first‐order behaviour.

**Table 1:** Compilation of kinetic data of H_3_B⋅NMeH_2_ dehydropolymerisation using complex **1** in toluene. For reactions following first‐order kinetics only *k_obs_* is given. For reactions following the described Michaelis–Menten model *K_M_* is given.

Entry	Reaction conditions^[a]^	*k_obs_* [min^−1^]	*K_M_* [mmol mL^−1^]	*k_2_* [min^−1^]
1	[**1**]=0.00675 m	1.189(8)×10^−1[b]^	–	–
2	[**1**]=0.00335 m	7.02(7)×10^−2[b]^	–	–
3	[**1**]=0.00167 m	8.0(1)×10^−2[c]^	2.60(3)×10^−1^	12.46(9)
4	[**1**]=0.00167 m, [H_3_B⋅NMeH_2_]=0.25 m	1.05(4)×10^−1[c]^	1.94(5)×10^−1^	12.3(2)
5	[**1**]=0.00167 m, [H_3_B⋅NMeH_2_]=0.17 m	1.25(5)×10^−1[c]^	1.13(3)×10^−1^	8.5(1)
6	[**1**]=0.00167 m, *T=*2 °C	1.60(9)×10^−2[c,d]^	5.4(2)×10^−2^	5.18(7)×10^−1^

[a] *T=*25 °C, [H_3_B⋅NMeH_2_]=0.33 m. [b] According to first‐order model. [c] Calculated according to Michaelis–Menten model: *k_obs_=(k_2_⋅E_0_)/K_M_*. [d] An induction period is observed for this reaction. Kinetic analysis was done for the post‐induction period region.

In an attempt to identify the nature of (Cat–S), we performed a low temperature in situ NMR experiment using [**1**]=0.00167 m and [H_3_B⋅NMeH_2_]=0.33 m. ^1^H and ^31^P NMR spectra after addition of fresh H_3_B⋅NMeH_2_ showed a significant broadening of the resonances of the *trans*‐dihydride [(PN*H*P)Fe(H)_2_CO] and loss of the ^1^H coupling pattern (Figure S28). Although this could indicate fluxional behaviour caused by formation of an adduct between Fe dihydride and amine borane, we cannot unequivocally assign this to formation of a new species.

Comparative volumetric studies with deuterium‐labelled substrates have shown that reactions with H_3_B⋅NMeH_2_ and D_3_B⋅NMeH_2_ can be described using the same kinetic model with a moderate kinetic isotope effect (KIE, *k_obs_*(H_3_B⋅NMeH_2_)/*k_obs_*(D_3_B⋅NMeH_2_)=1.5(1)). This value could point to the presence of a bent transition state.^28^ Alternatively, B−H activation could occur very early in the transition state. Dehydropolymerisation of H_3_B⋅NMeD_2_ shows a different kinetic profile with the rate of gas evolution showing a maximum (Figure S24). This could point to differences in the rate‐determining steps of dehydrogenation. Therefore, *k_obs_* and KIE values could not be derived for H_3_B⋅NMeD_2_. A similar problem was described before for the related Ru complex [(PNP)Ru(H)(PMe_3_)].8d

### Polymer characterisation

**Figure 2:**
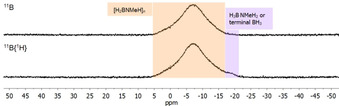
^11^B, ^11^B{^1^H} NMR (96 MHz, CDCl_3_) spectra of the isolated [H_2_BNMeH]_*n*_ from H_3_B⋅NMeH_2_ dehydropolymerisation using complex **1** (2 mol %) in toluene at *T=*25 °C and [H_3_B⋅NMeH_2_]=0.33 m.

Poly(aminoborane)s were isolated as pale‐yellow powders by precipitation into cold (−78 °C) *n*‐hexane. NMR spectroscopic data of the polymers are in line with literature values.1d, 15, 18 Other than in a previous study of our group on a dinuclear Zr catalyst,19a ^11^B NMR spectra show no pronounced resonances for BH_3_ end groups, indicating the presence of much higher‐molecular‐weight polymers (Figure 2). Closer inspection of the ^11^B NMR spectra shows that the BH_2_ signal is slightly shifted to higher field by 1.5 ppm for polymers prepared using **1** compared to those made using **2** in an earlier study (Figure S14).18d

Analysis of the polymers by size exclusion chromatography (SEC, light scattering (LS) detection giving absolute molecular weights)^29^ was done using sample concentrations of 2 mg mL^−1^ in THF using 0.1 wt % of tetrabutylammonium bromide, at *dn*/*dc* values of 0.09 mL g^−1^.19a For some of the samples, problems with reproducibility occurred due to low signal intensities; in these cases, the sample concentration was increased to 4 mg mL^−1^. It is once more noted that concentration sensitive refractive index (RI) detection in combination with standard calibration is commonly used for relative molecular weight determination of these materials. However, it is known that molecular weights will be overestimated by a factor of 3–6.^10^ To verify this fact for our set of samples made using Fe catalyst **1**, we applied both, RI and LS detection for selected samples and found that molecular weights *M_n_* and *M_w_* are larger by a factor of 2–10 using RI detection and polystyrene as calibration standard (see Table S3).

Table 2 shows a compilation of molecular weights of poly(aminoborane)s obtained using different reaction conditions. Higher concentrations of the catalyst **1** give higher molecular weights of the isolated polymers. Although this is significant, we do not regard this as evidence for step‐growth polymer formation, as in previous studies, a much more pronounced increase of *M_n_* with higher catalyst loading was reported.^11^ The presence of H_2_ has no significant influence on the molecular weight (entries 3 and 4). Notably, polymers formed in THF appear to be more uniform, giving much smaller dispersities *Ð* (Table S2). Repeated additions of H_3_B⋅NMeH_2_ in toluene resulted in full consumption of the substrate, however, molecular weights were not affected significantly (entry 3 vs. entry 5). Polymerisation is thus not living. However, that the reaction proceeded shows that the Fe catalyst is still intact and can be recharged. Notably, the same experiment in THF was not successful as the catalyst decomposed after the first run. Dilution of the reaction solution by a factor of 3 (entry 6) gave slightly lower molecular weights. Reactions at low temperature (*T=*2 °C) increased the molecular weights by a factor of 2 (entry 7).

**Table 2:** SEC–LS data.

Entry	Reaction conditions^[a]^	*t* [h]	*M*_n_ [g mol^−1^]^[b]^	*M*_w_ [g mol^−1^]^[b]^	*Ð*
1	[**1**]=0.00675 m	0.5	24 300	85 400	3.5
2	[**1**]=0.00335 m	1	18 900	78 600	4.2
3	[**1**]=0.00167 m	1.5	13 000	32 500	2.5
4	[**1**]=0.00167 m closed system	24	15 600	41 000	2.6
5	[**1**]=0.00167 m 3x H_3_B⋅NMeH_2_	3×1.5	11 300	36 550	3.2
6	[**1**]=0.00167 m [H_3_B⋅NMeH_2_]=0.11 m	2	11 400	28 100	2.5
7	[**1**]=0.00167 m *T=*2 °C	12	27 000	81 700	3.0
8	[**3**]=0.00335 m	48	4500	7000	1.6

[a] Toluene, *T=*25 °C, [H_3_B⋅NMeH_2_]=0.33 m. [b] Absolute molecular weights determined using light scattering detection.

**Figure 3:**
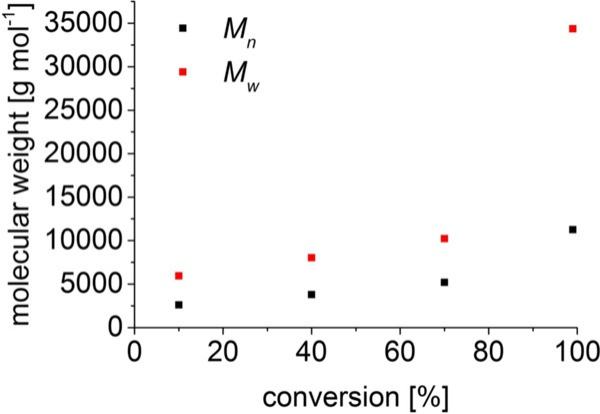
Poly(aminoborane) growth kinetics in toluene. Reaction conditions: 0.5 mol % **1**, [H_3_B⋅NMeH_2_]=0.33 m, *T=*25 °C, isobaric system connected to gas buret. Conversion was determined from volumetric data.

Analysis of molecular weights at different stages of the dehydropolymerisation reaction in toluene (Figure 3) shows that *M_n_* remains constant at low values throughout the reaction, whereas *M_w_* shows a significant increase towards the end of the reaction. This could be rationalised by a chain‐growth scenario in which depolymerisation occurs at low substrate concentrations (i.e. at high conversion). ^11^B NMR spectroscopy supports this assumption as polymer formation occurs directly at the onset of the reaction and short‐chain oligomers were not detected (Figure S16). A slightly different profile of molecular weights vs. conversion plots was observed by Weller in a recent study of [(dppp)Rh]^+^ catalysed dehydropolymerisation of H_3_B⋅NMeH_2_.18b In this report, *M_n_* remained constant up to high conversion (90 %) with a more pronounced increase towards the end of the reaction. Depolymerisation was not observed and NMR spectra of isolated polymers were similar throughout the course of the reaction, thus prompting the authors to conclude a hybrid chain‐growth/step‐growth mechanism.

### In situ NMR spectroscopy

Monitoring of the dehydropolymerisation reaction using ^11^B NMR spectroscopy shows that H_3_B⋅NMeH_2_ is initially converted into the polymer, [H_2_BNMeH]_*n*_ (*δ* −5 ppm, br s; Figure S16). As soon as the substrate is fully consumed, formation of small amounts of (HBNMe)_3_ is observed (*δ* 33 ppm), which is also reflected by the result of volumetric analysis, giving [*n*(H_2_)/*n*(H_3_B⋅NMeH_2_)] values close to 1.0. ^11^B{^1^H} NMR spectra show that the BH_2_ signal at −5 ppm is composed of two resonances, indicating the formation of small amounts of cyclotriborazane (H_2_BNMeH)_3_. Also, after consumption of H_3_B⋅NMeH_2_ minor amounts of diaminoborane HB(NMeH)_2_ (*δ* 28 ppm) are observed (Figure S16). Formation of such species was found before and was attributed to metal‐assisted B−N bond cleavage in amine boranes, followed by reaction of the amine with aminoborane.^30^ Alternatively, Paul and co‐workers computed a rearrangement reaction of aminoborane, H_2_B=NH_2_ to yield BH_3_ and HB(NH_2_)_2_ to be slightly exergonic by 3.5 kcal mol^−1^.^31^ The presence of diaminoboranes was discussed as indirect evidence for the presence of free aminoborane.

Addition of cyclohexene to trap transient aminoborane H_2_B=NMeH in toluene were not successful, indicating that either no free aminoborane is formed or hydroboration is much slower than B−N bond formation (Figure S42). Experiments in THF have shown that Cy_2_B=NMeH (*δ* 46 ppm) can only be detected in ^11^B NMR spectra after full consumption of H_3_B⋅NMeH_2_ when also formation of (HBNMe)_3_ takes place (Figure S41). This might indicate that H_2_B=NMeH is not involved in the polymer formation step, but at a later stage where also depolymerisation occurs to form (HBNMe)_3_. Notably, HB(NMeH)_2_ is formed already at earlier stages of dehydropolymerisation, further supporting the ambiguous role of species of this type.^3^, ^15^, 18b, 19b Schneider and co‐workers have shown that for H_3_B⋅NH_3_ dehydropolymerisation catalysed by **1**, Cy_2_B=NH_2_ can be observed, however, the authors only presented ^11^B NMR spectra at full conversion in presence of borazine.8c

Depolymerisation experiments using a catalyst‐free higher molecular weight sample of (H_2_BNMeH)_*n*_ 32 have shown that formation of (HBNMe)_3_ and HB(NMeH)_2_ only occurs in the presence of the Fe catalyst (Figure S44). Also, as shown by an experiment using freshly prepared (H_2_BNMeH)_3_,^33^ the latter is readily converted into (HBNMe)_3_ (Figure S48). Notably, free H_2_B=NMeH could only be trapped as Cy_2_B=NMeH for depolymerisation reactions in THF, again suggesting that in THF hydroboration is much faster than in toluene.

### Dehydrocoupling of H_3_B⋅NMe_2_H with amido complex 1

**Scheme 3:**
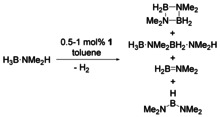
Dehydrocoupling of H_3_B⋅NMe_2_H using complex **1**.

To gain further insights into the role of the aminoborane and the role of the Fe centre for B−N bond formation, we have performed similar reactions using H_3_B⋅NMe_2_H as a model compound and **1** as the catalyst (Scheme 3).

**Figure 4:**
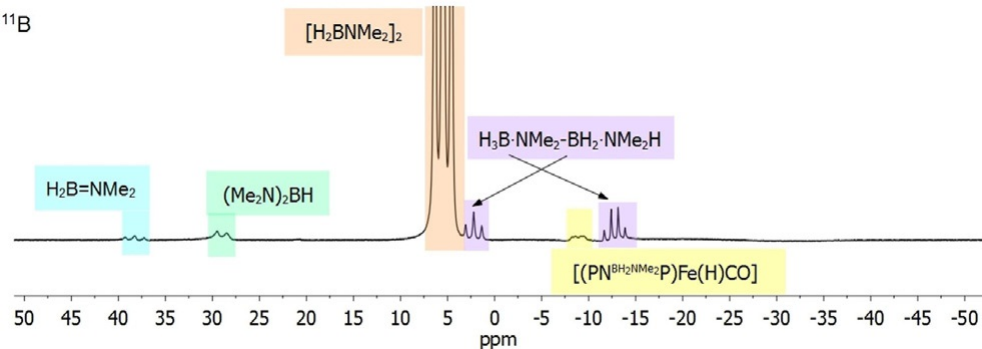
In situ ^11^B NMR spectrum (96 MHz, 298 K, 1024 scans) of H_3_B⋅NMe_2_H dehydrocoupling in [D_8_]toluene. Reaction conditions: 1 mol % **1**, *T=*25 °C and [H_3_B⋅NMe_2_H]=0.33 m.

Volumetric analysis of reactions using **1** in toluene shows that one equivalent of H_2_ is produced within two hours (Figure S61). Under these conditions, H_2_ release follows first‐order behaviour, most likely due to the higher solubility of the substrate compared to H_3_B⋅NMeH_2_. ^11^B NMR analysis of the reaction mixture indicates that H_3_B⋅NMe_2_H is quantitatively converted into the main product cyclic diborazane (H_2_BNMe_2_)_2_ (*δ* 5.5 ppm). Additionally, minor amounts of aminoborane H_2_B=NMe_2_ (*δ* 38.2 ppm), HB(NMe_2_)_2_ (*δ* 28.9 ppm), and the linear diborazane H_3_B⋅NMe_2_BH_2_⋅NMe_2_H (*δ* −12.9 and 2.1 ppm), were detected (Figure 4).^34^

Similarly as for reactions with H_3_B⋅NMeH_2_, a broad doublet at *δ* −9.0 ppm (^1^ *J*_B,H_=110 Hz) can be assigned to an aminoborane capped Fe amido species [(PN${}^{\mathrm{BH}{}_{2}\mathrm{NMe}{}_{2}}$ P)Fe(H)CO] (**1‐H_2_BNMe_2_**). In previous studies, the presence of H_3_B⋅NMe_2_BH_2_⋅NMe_2_H was discussed as evidence for the existence of an on‐metal pathway for B−N coupling.9b, 11, 34 It should however be noted that free aminoborane could originate either from metal‐based dehydrogenation of amine borane and release from the active species, or from dehydrogenation of linear diborazane, which was computed to be thermodynamically favourable and observed in case of the related Ru complex [(PNP)Ru(H)PMe_3_].9b

### Dehydropolymerisation with *N*‐methyl Fe amine complex 3

**Scheme 4:**
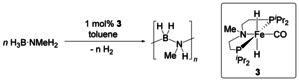
Dehydropolymerisation of H_3_B⋅NMeH_2_ using complex **3**.

To evaluate the role of cooperative effects between the PNP ligand and the Fe centre, we have synthesised the literature‐known related *N*‐methylated complex **3** 35 and tested this as a catalyst for H_3_B⋅NMeH_2_ dehydropolymerisation (Scheme 4). A bifunctional dehydrogenation and B−N coupling pathway that involves ligand cooperation should not be accessible using this complex. As a result, *N*‐methylation could have consequences for both, dehydrogenation rate and product selectivity.

**Figure 5:**
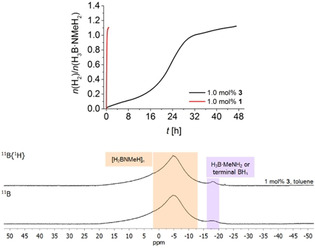
Top: Volumetric curves of H_3_B⋅NMeH_2_ dehydropolymerisation using complex **3** in toluene at *T=*25 °C and [H_3_B⋅NMeH_2_]=0.33 m. Bottom: ^11^B, ^11^B{^1^H} NMR (96 MHz, CDCl_3_) spectra of the isolated [H_2_BNMeH]_*n*_.

Figure 5 shows a comparison of volumetric curves of H_3_B⋅NMeH_2_ dehydropolymerisation using complexes **1** and **3**. Evidently, reactions using **3** are much slower compared to **1**. To our surprise, reactions in toluene show a long induction period, which was not observed in any of the other cases, but is known for reactions using the borate complex **2** in THF.18d We discount Fe nanoparticle formation as the reason for this long induction period as PMe_3_ poisoning experiments gave the same reaction profile and full conversion.

Complex **3** exhibits sufficient stability for NMR monitoring in toluene. At the onset of the dehydrogenation reaction in situ ^11^B NMR spectra show virtually the same product distribution as for complex **1** (Figure S52), namely a broad resonance at *δ* −5 ppm for poly(aminoborane) along with a signal at *δ* −18 ppm for residual H_3_B⋅NMeH_2_ or the BH_3_ end group of a shorter‐chain polymer. Upon further dehydropolymerisation, ^11^B NMR spectra become more complex, showing resonances for aminodiborane BH_2_(*μ*‐MeNH)(*μ*‐H)BH_2_ (*δ* −22 ppm), boronium species [BH_2_(MeNH_2_)_2_]^+^ (*δ* −9.0 ppm, t, *J*_BH_ *=*106 Hz; *cf*. −8.9 ppm, *J*_BH_=108 Hz18c) as well as hitherto unobserved signals at *δ* 0.3 and *δ* −1.5 ppm that could point to the presence of further boronium compounds or polymer branching.^3^ To our surprise we succeeded in detecting Cy_2_B=NMeH as the product of H_2_B=NMeH trapping at a later stage of the reaction, again suggesting that aminoborane might be more relevant for depolymerisation than for polymer formation.

Workup of the B−N products was done as before by precipitation into cold *n*‐hexane, however, in this case significantly lower polymer yields were observed. SEC analysis showed much lower molecular weights for samples made using the *N*‐methylated catalyst **3** (*M_n_*=4 500 g mol^−1^, Table 2). The low‐molecular‐weight nature of the material made using **3** is also reflected in NMR spectra of the isolated products, showing a more pronounced resonance for a BH_3_ end group (Figure 5).

### Dehydropolymerisation with borane capped complex 1‐BH_3_

**Figure 6:**
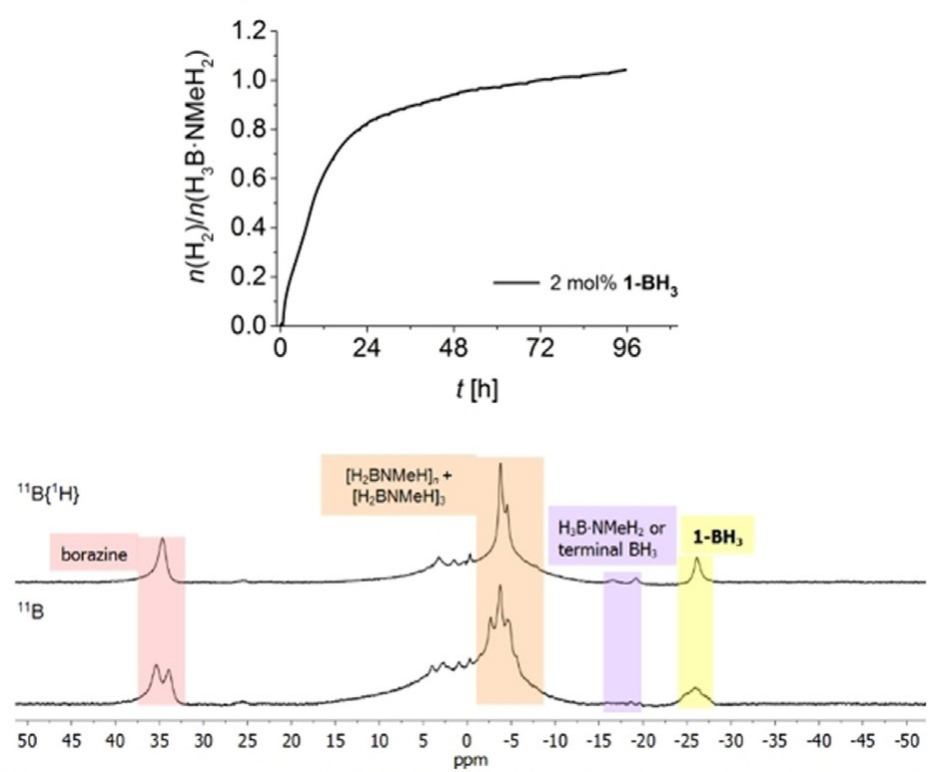
Top: Volumetric curve of dehydropolymerisation of H_3_B⋅NMeH_2_ using complex **1‐BH_3_** in toluene at *T=*25 °C and [H_3_B⋅NMeH_2_]=0.33 m. Bottom: In situ ^11^B, ^11^B{^1^H} NMR (96 MHz, [D_8_]toluene) spectra recorded at the end of the reaction.

In a previous study, we have reported the formation of a borane capped Fe amido complex [(PN^BH3^P)Fe(H)CO] **1‐BH_3_** during catalysis.18d As mentioned above, similar aminoborane species were detected before in dehydrocoupling of H_3_B⋅NMe_2_H using a related Ru complex, however, this was found to show poor reactivity with amine borane.9b To further evaluate the role of such species in dehydrogenation and growth of the B−N polymer, we have prepared **1‐BH_3_** by addition of BH_3_ to complex **1** and tested this in catalysis. Although this species is much less active than **1**, poly(aminoborane) is formed, but the dominant species is the cyclic borazane, clearly visible by the triplet in the in situ ^11^B NMR spectra (Figure 6). ^11^B{^1^H} NMR spectra of the isolated polymer show a relatively sharp resonance for the BH_2_ moiety and a pronounced resonance at −18 ppm, which could indicate residual starting material or BH_3_ end groups of a short‐chain poly(aminoborane) (Figure S35).

Low activity of **1‐BH_3_** can be traced back to the fact that no highly active dihydride complex [(PN*H*P)Fe(H)_2_CO] is accessible during catalysis. Instead, exclusively **1‐BH_3_** is present even after reaction times of 96 hours (Figure S32). Although the mechanism of dehydrogenation using this complex is unclear, it is evident from in situ ^11^B NMR spectra that B−N coupling occurs less selectively. Signals at −5<*δ*<5 ppm suggest the presence of significant amounts of branched products. Also, much more (HBNMe)_3_ was produced with **1‐BH_3_** as the catalyst, further suggesting the presence of an alternative B−N coupling pathway, possibly without involvement of the metal complex.

### Mechanistic considerations

**Scheme 5:**
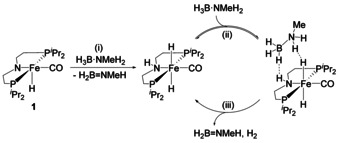
Proposed mechanism of H_3_B⋅NMeH_2_ dehydrogenation using precatalyst **1**.8c, 22 It should be noted that this scheme does not reflect all reaction conditions. As shown above, the equilibrium (ii) is determined by the experimental conditions, that is, solvent, [**1**] and [H_3_B⋅NMeH_2_].

Taken together, the above observations indicate that H_3_B⋅NMeH_2_ dehydropolymerisation takes place through a chain‐growth mechanism in which the Fe catalyst is bifunctional and involved in both, dehydrogenation and B−N bond formation. This is reflected in comparisons of in situ ^11^B NMR spectra and the observed trends in molecular weights with changes in catalyst concentration (Table 2) and at different conversions (Figure 3). Methylation of the nitrogen backbone of the pincer ligand in complex **3** leads to H_2_ release that is slower by approximately a factor of 10–15 (Figure 5), thus suggesting that for **1** the dehydrogenation predominantly occurs through a well‐known metal‐ligand bifunctional mechanism. Mechanistic models for related catalytically active *N*‐alkylated pincer complexes were described before;^36^ a similar scenario should be operating in the herein reported case. In a previous experimental and theoretical investigation of hydrazine borane dehydrocoupling using complex **1**, we have proposed facile, essentially barrier‐free, activation of the Fe amido moiety by the amine borane substrate to yield the dihydride complex [(PN*H*P)Fe(H)_2_CO].^22^ This subsequently reacts with further substrate to eliminate hydrogen and the aminoborane. Based on these data as well as the kinetic and spectroscopic studies reported herein (vide supra), we suggest a similar scenario for the dehydrogenation of H_3_B⋅NMeH_2_ (Scheme 5):

 

activation of the precatalyst **1** by proton/hydride transfer across the Fe amido bond to form the resting state complex [(PN*H*P)Fe(H)_2_CO]

 

formation of a transient catalyst–substrate complex between [(PN*H*P)Fe(H)_2_CO] and H_3_B⋅NMeH_2_

 

B−H and N−H activation, followed by hydrogen release and formation of the aminoborane. The KIE for B−H/B−D being small supports the presence of a non‐linear transition state or B−H activation occurring very early in the transition state.

 

**Scheme 6:**
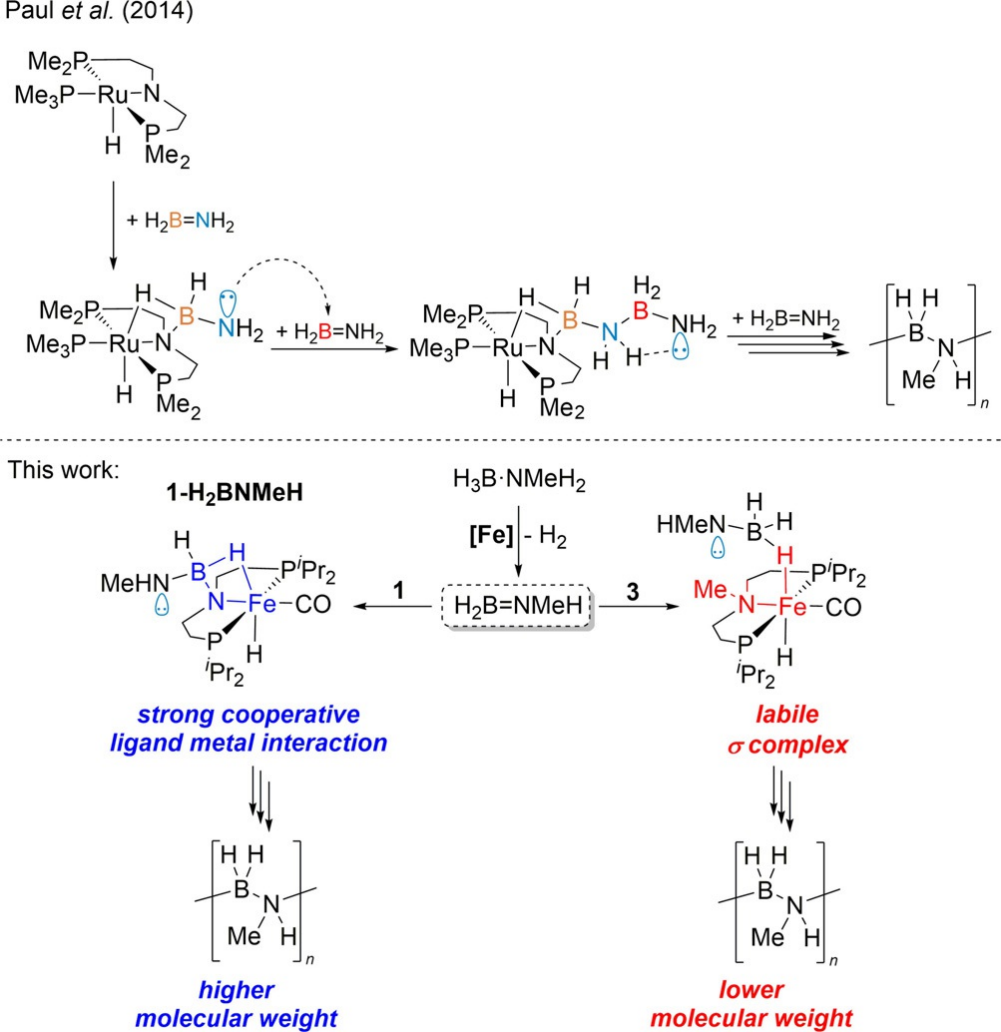
Proposed poly(aminoborane) formation from Fe amido complex **1** and *N*‐methylated Fe amine complex **3**, resulting in high‐ and low‐molecular‐weight polymers.

That the distribution of B−N products is similar for complex **1** and the N‐methylated complex **3**, but poly(aminoborane)s are different in molecular weight, suggests that chain propagation occurs at the metal complex. More specifically, chain growth occurs through interaction of the growing chain with the metal centre, but involvement of the PNP ligand is beneficial to obtain higher molecular weight polymers. For the related Ru system [(PNP)Ru(H)PMe_3_], Paul and co‐workers have suggested interaction of the aminoborane with the Ru centre and the amido group of the ligand, followed by nucleophilic chain growth through the end of the growing polymer chain (Scheme 6).^17^ Stoichiometric experiments of **1** with H_3_B⋅NMeH_2_ and H_3_B⋅NMe_2_H confirmed the formation of such species [(PN${}^{\mathrm{BH}{}_{2}\mathrm{NMeH}}$ P)Fe(H)CO] **1‐H_2_BNMeH** (Figure S54) and [(PN${}^{\mathrm{BH}{}_{2}\mathrm{NMe}{}_{2}}$ P)Fe(H)CO] **1‐H_2_BNMe_2_**. Based on the NMR spectroscopic observation of aminoborane‐capped Fe amido species and the found trends in *M_n_* (Table 2) we propose that in the herein described Fe system a similar scenario could explain the formation of high‐molecular‐weight polymers for **1**. In contrast, the absence of such interactions in catalysis with **3** only allows for the formation of comparably low‐molecular weight polymers. Stoichiometric reactions of **3** with H_3_B⋅NMeH_2_ (Figure S60) show no other species than the precatalyst.

Involvement of complexes of the type **1‐BH_3_** in dehydrogenation and B−N coupling using **1** is less likely as rates of H_2_ release were much lower and product distributions were different compared to reactions with **1** or **3**. We thus conclude that **1‐BH_3_** rather represents an off‐cycle species with limited relevance to the dehydropolymerisation catalysis using **1**.

### Synthesis and characterisation of silyl functionalised amine boranes

Heteroatom functionalised poly(aminoborane)s are rather rare, to the best of our knowledge, only a handful of examples have been presented to date.^11^ Silicon containing B−N polymers are promising single‐source precursors that could possess new interesting properties, for example, for coating applications. Also, inclusion of Si into B−N ceramics could furnish new materials with unforeseen thermal properties.^37^, ^38^ We have therefore prepared an example for a silyl substituted amine borane and tested this for dehydropolymerisation.

**Scheme 7:**
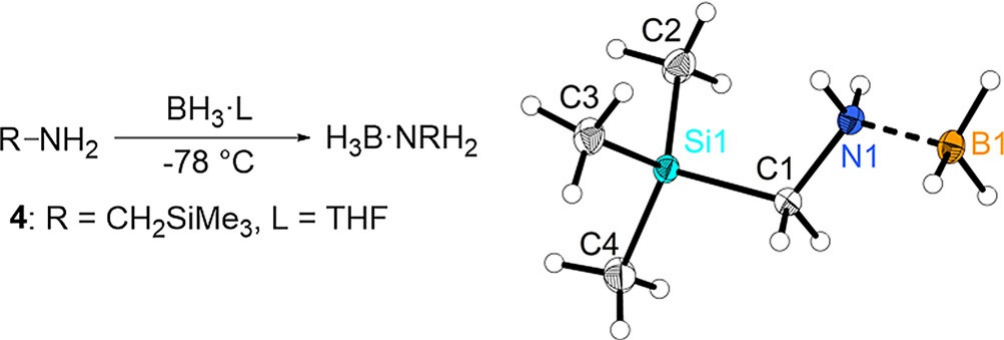
Synthesis of silyl functionalised amine borane and molecular structure of compound **4**. Thermal ellipsoids correspond to 30 % probability. Deposition Number 1961735 contains the supplementary crystallographic data for this paper. These data are provided free of charge by the joint Cambridge Crystallographic Data Centre and Fachinformationszentrum Karlsruhe Access Structures service www.ccdc.cam.ac.uk/structures.

H_3_B⋅N(CH_2_SiMe_3_)H_2_ (**4**) was prepared in analogy to the procedure established for H_3_B⋅NMeH_2_ 39 (Scheme 7), giving the product as a white, crystalline solid that can be purified by sublimation.

^1^H NMR spectroscopic analysis of **4** shows the expected signals due to SiMe_3_ (*δ* 0.11 ppm), BH_3_ (*δ* 1.53 ppm), CH_2_ (*δ* 2.28 ppm), and NH_2_ group (*δ* 3.71 ppm). In ^11^B NMR, an ill‐resolved quartet can be detected at −17.5 ppm (Figure S3). Crystals suitable for an X‐ray analysis were obtained by slow cooling of a saturated solution of **4** in diethyl ether to −30 °C. The molecular structure is depicted in Scheme 7. As found for other amine boranes, the B−N bond (1.604(2) Å) in compound **4** is slightly elongated compared to a typical B−N single bond (Σ*r*_cov_=1.56 Å^40^, H_3_B⋅NMeH_2_: 1.594(1) Å^41^).

### Dehydropolymerisation of functionalised amine borane 4

For initial dehydropolymerisation experiments using **4** we tested two well‐studied amine borane dehydrogenation catalysts, namely Brookhart's [(POCOP)IrH_2_]1d, 8g, 13, 42 (POCOP=2,6‐(*t*Bu_2_PO)_2_‐C_6_H_3_) as well as commercially available [Rh(cod)Cl]_2_ 39, 43 (cod=1,5‐cyclooctadiene) in THF at room temperature. Despite using comparably high catalyst loadings of 5 mol % release of less than one equivalent of H_2_ was found and reactions were stopped after 24 hours (Figure S63). [(POCOP)IrH_2_] is known to be less tolerant towards sterically more demanding amine borane adducts^10^ and functional groups close to the amine moiety,^12^ which is in line with our observation of release of only 0.4 equivalents of H_2_ and the presence of residual substrate **4** in ^11^B NMR spectra. Furthermore, diaminoborane HB(NHR)_2_ (*δ* 28.5 ppm) and the corresponding borazine (HBNR)_3_ (*δ* 32.0 ppm, R=CH_2_SiMe_3_) were detected (Figure S64, Table S4). When using the Rh precursor, the dehydrogenation reaction was much faster, however, after 24 hours only approximately 0.7 equivalents of H_2_ were released. In situ ^11^B NMR spectra show the above‐mentioned resonances as well as additional signals that we assign to aminodiborane species (BH_2_)_2_(*μ*‐RNH)(*μ*‐H) (*δ* −22.0 ppm, R=CH_2_SiMe_3_).

**Figure 7:**
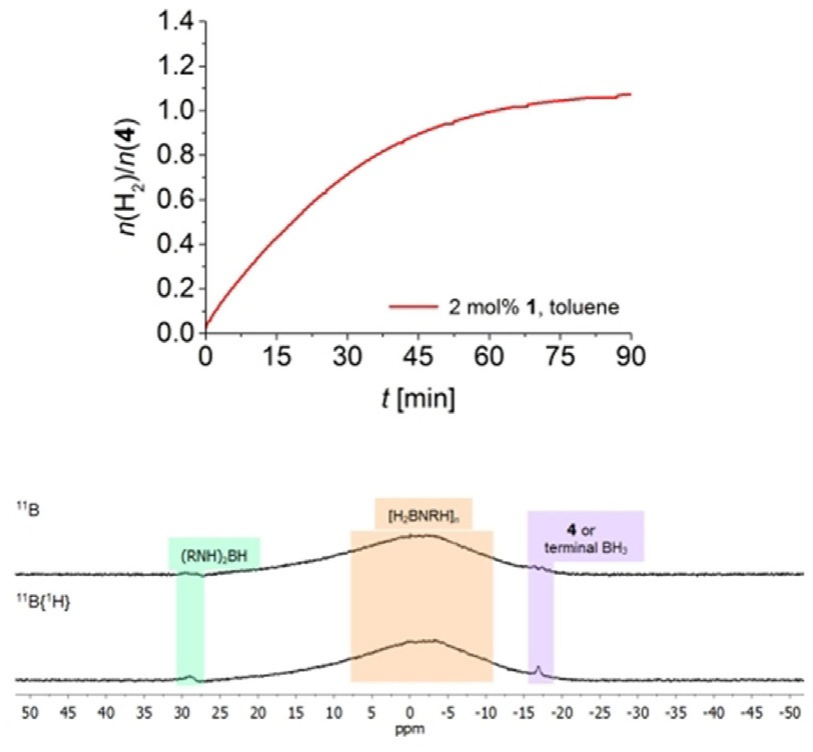
Top: Volumetric curve of dehydropolymerisation of **4** using complex **1** in toluene at *T=*25 °C and [H_3_B⋅NRH_2_]=0.21 m. Bottom: In situ ^11^B NMR spectra (96 MHz, [D_8_]toluene) recorded after full conversion. R=CH_2_SiMe_3_.

Based on the above‐described results on H_3_B⋅NMeH_2_ dehydropolymerisation and Schneider's related work on H_3_B⋅NH_3_ we have next tested the Fe amido catalyst **1** for dehydropolymerisation of **4**. Reactions in THF were very slow, requiring 14 days for release of one equivalent of H_2_ (Figure S65). Most remarkably, changing the solvent to toluene has a dramatic effect as in this case full conversion was observed after only 90 minutes (Figure 7, top) along with an increase in viscosity of the reaction mixture. We thus note that complex **1** is much more tolerant toward sterically demanding groups at the methyl group of the amine borane than the well‐studied catalyst [(POCOP)IrH_2_].

^11^B NMR analysis of the reaction mixture shows a pronounced broad signal centred at approximately *δ* −2 ppm (Figure 7, bottom). Additional resonances at *δ* 29 and −17 ppm indicate the presence of HB(NHR)_2_ and BH_3_ end‐groups of a polymer or unreacted **4**, respectively. Unfortunately, after precipitation of the polymer into *n*‐hexane, the pale‐yellow precipitate was insoluble in commonly used solvents THF or CHCl_3_, thus complicating further analysis by NMR (Figures S73 and S74) or SEC.

**Scheme 8:**
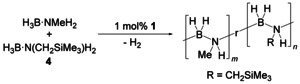
Co‐dehydropolymerisation of H_3_B⋅NMeH_2_ and **4** to yield a random copolymer. r used to link both monomer subunits in the polymer structure is used to indicate that the copolymer is most likely random.

H_3_B⋅NMeH_2_ and **4** show similar rates in dehydropolymerisation reactions using **1** as the catalyst. We thus envisioned a co‐dehydropolymerisation reaction to increase the solubility of the Si containing poly(aminoborane) (Scheme 8), an approach that was reported to be feasible before.1d, 10, 11, 12

**Figure 8:**
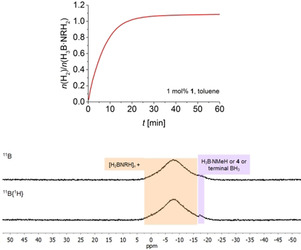
Top: Volumetric curve of co‐dehydropolymerisation of an equimolar mixture of H_3_B⋅NMeH_2_ and **4** using complex **1** at *T=*25 °C and [H_3_B⋅NRH_2_]=0.33 m. Bottom: ^11^B NMR spectra (96 MHz, CDCl_3_) of isolated co‐polymer after co‐dehydropolymerisation of H_3_B⋅NMeH_2_ and **4**. R=Me or CH_2_SiMe_3_.

As before for reactions with **4**, reactions of equimolar mixtures of H_3_B⋅NMeH_2_ and **4** in toluene show full conversion, in this case after only 30 minutes and albeit using a lower catalyst loading (1 instead of 2 mol % **1**; Figure 8, top). In situ ^11^B NMR spectra show a broad resonance at *δ* −5 ppm without any detectable proton coupling, typical for a polymer. Two minor additional signals at *δ* 29 and 33 ppm indicate formation of HB(NHR)_2_ and (HBNR)_3_, respectively (Figure S84). Reactions in THF were again much slower and did not show full conversion, producing only 0.6 equiv. of H_2_ (Figure S74).

Other than for *N*‐methylpoly(aminoborane), workup proved to be rather difficult in this case. Addition of *n*‐hexane at −78 °C did not lead to precipitation and only resulted in turbid solutions from which no (filter paper) or only small amounts (5–10 % yield, short plug of Al_2_O_3_) of polymer could be isolated by filtration. To our delight, MeCN proved to be well‐suited for workup. Concentration of the reaction solution to a minimum volume and addition of MeCN resulted in the formation of white plates, which were isolated and dried in vacuum (40 % yield). Notably, when changing the amine borane ratio [**4**]:H_3_B⋅NMeH_2_ to 3:1, yields could be improved to 60 % of a random 3:1 copolymer. The ^11^B{^1^H} NMR spectrum of the isolated 1:1 co‐polymer shows the aforementioned broad resonance at *δ* −5 ppm for the BH_2_ groups of the polymer chain (Figure 8, bottom). ^1^H NMR analysis reveals the expected signals for CH_2_SiMe_3_ (*δ* 2.0 and 0.12 ppm) and CH_3_ groups (*δ* 2.23 ppm), which are in in the same range as found for the homopolymers (Figure S78).1d ^1^H NMR integral ratios, the lack of signals in ^11^B NMR spectra in the region −5<*δ*<5 ppm and the uniform main signal for the BH_2_ groups suggest the presence of a linear random polymer without significant branching. Soluble polymer isolated from toluene solutions shows a molecular weight (SEC, LS detection) of *M*_n_=11 400 g mol^−1^ (*M*_w_=14 600 g mol^−1^) with comparably narrow dispersity of *Đ*=1.26.

**Figure 9:**
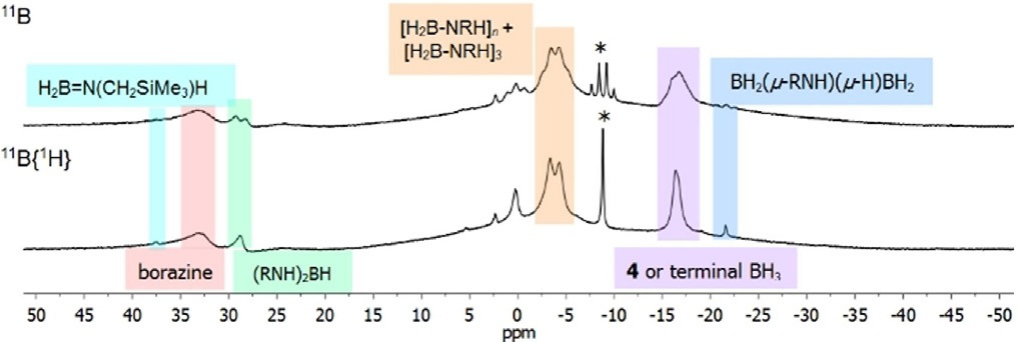
In situ ^11^B and ^11^B{^1^H} NMR spectra (96 MHz, [D_8_]toluene) after dehydropolymerisation of **4** in toluene using 0.5 mol % **1** and 10 mol % NMe_2_Et at *T=*25 °C and [**4**]=0.33 m. R=CH_2_SiMe_3_, *=H_3_B⋅NMe_2_Et_._ 8c

As shown above for reactions with H_3_B⋅NMeH_2_, a decrease in the catalyst loading results in the formation of lower‐molecular‐weight polymers. We have thus repeated dehydropolymerisation of pure **4** with less catalyst **1** to obtain a well‐defined, more soluble homopolymer derived from **4**. Use of 0.5 mol % **1** instead of 2 mol % (vide supra) in toluene gave no full conversion (release of 0.6 equivalent of H_2_, Figure S75), most likely due to BH_3_ deactivation of the active dihydrido species that was discussed before by Schneider.8c In that case, addition of an amine was found to increase the stability of the catalyst. In a more recent study, Weller and co‐workers have shown that for [Rh(DPEphos)]^+^ and Rh pincer complexes amine addition serves to bring the catalyst on‐cycle.18a, 44 We have thus added NMe_2_Et and found that this gives full turnover (i.e. release of one equivalent of H_2_) after 14 hours. NMR analysis of the reaction solution (Figure 9) shows that the product mixture is however rather complex, containing (HBNR)_3_, HB(NHR)_2_, (H_2_BNRH)_3_, and (BH_2_)_2_(*μ*‐RNH)(*μ*‐H) along with the polymer. A well‐defined quartet resonance for the product of BH_3_ trapping, H_3_B⋅NMe_2_Et is found at *δ* −8.8 ppm. Notably, a minor resonance at *δ* 37.5 ppm indicates the presence of aminoborane H_2_B=NRH, which was not observed directly before for other primary amine boranes.

Addition of cold *n*‐hexane to the product mixture results in precipitation of the polymer, which could be isolated in approximately 40 % yield. Again, the ^11^B and ^1^H NMR spectra show the expected broad signals (^11^B: *δ* −7 ppm; ^1^H: *δ* 0.13, 1.86, 2.03, 2.93 ppm, Figure S76), supporting the assignment as a homopolymer derived from **4**. Signals for BH_3_ end groups of the polymer were not observed. The ^29^Si{inept} NMR spectrum shows a broad resonance at *δ* 1 ppm that is slightly shifted to higher field compared to **4** (*δ* 0.6 ppm). SEC analysis (LS detection) confirmed the presence of a well‐defined high‐molecular‐weight polymer (*M*_n_=61 200 g mol^−1^, *M*_w_=98 500 g mol^−1^, *Đ*=1.6).

## Conclusions

Dehydropolymerisation of methylamine borane and a silyl substituted analogue using the iron amido complex **1** furnishes high‐molecular‐weight poly(aminoborane)s. Kinetic studies of H_3_B⋅NMeH_2_ dehydrogenation show that this process follows a kinetic regime which can be described as a first‐order process as a limiting case of more general Michaelis–Menten‐type kinetics with the iron dihydride [(PN*H*P)Fe(H)_2_CO] as the active species. Analysis of the molecular weights of the obtained polymers, comparative studies using a related *N*‐methylated catalyst and dimethylamine borane suggest involvement of the metal complex. We propose that polymer growth occurs through well‐precedented nucleophilic chain growth from the end of a coordinated oligomer chain in a chain‐growth scenario. Control of the molecular weight of the polymers is possible by variation of the catalyst concentration, reaction temperature, the solvent as well as the structure of the Fe catalyst. Reactions in toluene are significantly faster and much more selective for poly(aminoborane) compared to those in THF.

The facile synthesis of a Si‐substituted primary amine borane has allowed for an extension of dehydropolymerisation studies to more functional substrates. Sterically demanding trimethylsilyl groups were not found to interfere with the dehydropolymerisation process when complex **1** was used in toluene. In fact, this precatalyst was found to be superior to other late transition‐metal complexes typically used in amine borane chemistry. The presented results once more highlight the great potential of Fe PNP complexes, not only for catalytic hydrogenation and dehydrogenation reactions directed at the synthesis of organic compounds and small molecule activation,^45^ but also for the synthesis of novel main group polymers. Future studies on this and related systems should be directed at the rational design of ligand and catalyst structures to not only control the molecular weights, but also the microstructure (branching, tacticity, incorporation of further monomer units) of the poly(aminoborane)s.

## Conflict of interest

The authors declare no conflict of interest.

## Supporting information

As a service to our authors and readers, this journal provides supporting information supplied by the authors. Such materials are peer reviewed and may be re‐organized for online delivery, but are not copy‐edited or typeset. Technical support issues arising from supporting information (other than missing files) should be addressed to the authors.

Supplementary
