# Supplementary material for: Dehydropolymerisation of Methylamine Borane and an N‐Substituted Primary Amine Borane Using a PNP Fe Catalyst
Source: Chemistry. 2020 May 28;26(35):7889–99. doi: 10.1002/chem.202000809 (PMC7383739; doi:10.1002/chem.202000809)
Supplement: Supplementary file 1 — Supplementary [file CHEM-26-7889-s001.pdf]

Chemistry–A European Journal

Supporting Information

Dehydropolymerisation of Methylamine Borane and an *N*-Substituted Primary Amine Borane Using a PNP Fe Catalyst

Felix Anke,[a] Susanne Boye,[b] Anke Spannenberg,[a] Albena Lederer,[b, c] Detlef Heller,[a] and Torsten Beweries*[a]

Supporting Information

Dehydropolymerisation of methylamine borane and an *N*-substituted primary amine borane using a PNP Fe catalyst

Felix Anke[a], Susanne Boye[b], Anke Spannenberg[a], Albena Lederer[b,c], Detlef Heller[a], Torsten Beweries*[a]

Table of content

Experimental Section ... **2**
Catalytic dehydropolymerisation of $H_3B{\cdot}NMeH_2$ ... **9**
Polymer analysis ... **23**
***In situ* NMR spectroscopy** ... **25**
Catalytic dehydrocoupling of $H_3B{\cdot}NMe_2H$ ... **34**
Catalytic dehydrocoupling of $H_3B{\cdot}N(CH_2SiMe_3)H_2$ ... **35**
References ... **45**

Experimental Section

General: All manipulations were carried out in an oxygen- and moisture-free argon atmosphere using standard Schlenk and drybox techniques. The solvents were purified with the Grubbs-type column system "Pure Solv MD-5" and dispensed into thick-walled glass Schlenk bombs equipped with Young-type Teflon valve stopcocks.
$H_3B{\cdot}NMeH_2$ (Boron Specialties) was used without further purification. Alternatively, $H_3B{\cdot}NMeH_2$ was produced according to a protocol described by Manners *et al.*, purified by sublimation (30 °C, $1{\cdot}10^{-3}$ mbar) before use and stored in the glovebox at -30 °C.[1] $H_3B{\cdot}NMe_3$ (Sigma) was sublimed prior to use and stored in the glovebox. [(dppp)Rh(nbd)][BF_4] was synthesised according to a literature procedure.[2] D_2 (Linde), $NMeH_2$ (2 M in THF, Sigma) and $N(CH_2SiMe_3)H_2$ (ABCR) were used as received. The iron complexes **1**[3], **1-BH₃**[4] and **3**[5-6] were synthesised according to a literature procedure and stored in the glovebox at -30 °C to prevent decomposition. [(POCOP)IrH_2] was prepared according to a literature procedure.[7] [Rh(cod)Cl]$_2$ (TCI Chemicals) was used as received.

NMR spectroscopy: NMR spectra were recorded on Bruker AV300 and AV400 spectrometers. ^{1}H, ^{13}C and ^{29}Si chemical shifts are given in ppm and were referenced to the tetramethylsilane and the solvent signal: THF-d_8: δ^{1}H: 1.73, 3.58, δ^{13}C: 25.4, 67.6; $CDCl_3$: δ^{1}H: 7.26, δC 77.2; toluene-d_8: δ^{1}H: 2.08, 6.97, 7.01, 7.09, δ^{13}C: 20.4, 125.1, 127.9, 128.9, 137.5; C_6D_6: δ^{1}H: 7.16, δ^{13}C: 128.1; $CDCl_3$: δ^{1}H: 7.26, δ^{13}C: 77.1. For the ^{31}P{^{1}H} NMR spectra H_3PO_4 was used as external standard. The baselines of ^{11}B and ^{11}B{^{1}H} NMR spectra have been corrected and the spectra were referenced to $BF_3{\cdot}Et_2O$ as external standard.
MS analysis: Mass spectra were recorded on a MAT 95XP Thermo Fisher mass spectrometer in electrospray ionization mode.
IR spectroscopy: IR active substances were measured on an FT-IR spectrometer Alpha from Bruker in ATR mode. The vibration bands are given in cm^{-1}.
CHN analysis: Samples for elemental analysis were prepared in the glovebox and measured on a microanalyser TrueSpec CHNS (Leco) by combustion analysis. The results of the elemental analysis are given in percent.
Gas chromatography: Using a gas-tight syringe, the gas samples were taken from the reaction chamber and injected splitless into the gas chromatograph (Agilent 7890A). The gas mixture was separated by a column of the type 60/80 Carboxen 1000 (Supelco) and registered with a thermal conductivity detector.
X-ray analysis: Diffraction data for **4** were collected on a Bruker Kappa APEX II Duo diffractometer. The structure was solved by direct methods (SHELXS-97)[8] and refined by full-matrix least-squares procedures on F^2 (SHELXL-2014).[9] Diamond[10] were used for graphical representations. CCDC 1961735 contains the supplementary crystallographic data for this paper. These data are provided free of charge by The Cambridge Crystallographic Data Centre.
SEC analysis: SEC analysis was performed for molecular weight determination. The SEC system consists of an isocratic pump series 1200 (Agilent Technologies, US), an autosampler series 1100 (Agilent Technologies, US), a refractive index (RI) detector Dn-2010 (λ=620 nm, Bures, DE), a multi-angle laser light scattering detector (MALLS, Wyatt Technologies, US). For the separation a PL MIXED-C column (300x7.5 mm, 5 µm PSgel, Agilent Technologies, US) was used. The flow rate was 1.0 mL/min at 25°C. The calculation of the molecular weights from the MALLS detector was performed by taking into account the dn/dc of the samples, which were determined by direct injection into the RI detector of samples with varied concentration.[11]
All samples (2 or 4 mg mL^{-1}) were prepared in air, then filtered through Ministart SRP 15 syringe filter (PTFE-membrane, pore size: 0.2 µm) and measured immediately. The eluent consists of THF and 0.1 wt% *n*-Bu_4NBr (Sigma).
Analysis of volumetric data: Volumetric studies were done using an automatic gas buret that operates under isobaric conditions. These conditions allow for the registration of the volume change of the setup which corresponds to the amount of gas evolved during the

dehydropolymerisation reaction. Details of the experimental setup were published before.[12-14] Kinetic analysis of the volumetric data was done using the program "Canalys".[15]
At the beginning of the catalytic experiments (2 to 3 minutes) significant gas evolution was detected in THF. This was much less pronounced in toluene. The detected volume can be attributed to adjustment of the equilibrium between liquid and gas phase due to vapor pressure and thermal effects. This was confirmed by blank experiments (Figure S1). To determine the amount of H_2 in catalytic experiments the overall volume was corrected by the vapor pressure of the solvent at T = 25°C.[13] The first part (t = 3 min) was not considered for kinetic analysis.

$$V_{Hydrogen} = \frac{nRT}{p^*} \qquad p^* = \frac{p(atmosphere) - p(solvent)}{p(atmosphere)}$$

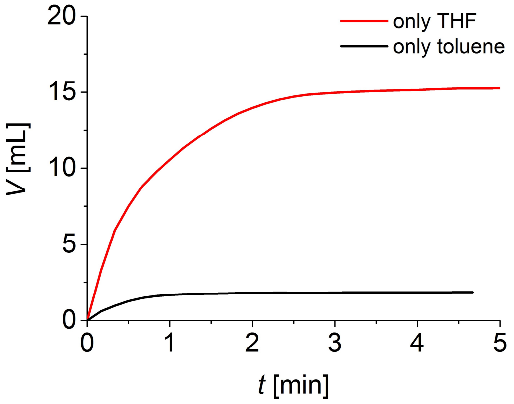

Figure S1. Volumetric curves from blank experiments using only THF or toluene.

General procedure for the catalytic dehydropolymerisation of $H_3B·NMeH_2$: $H_3B·NMeH_2$ (60 mg, 1.33 mmol) and the corresponding catalyst were weighed in the glovebox and transferred to a three-necked reaction vessel. Then, $H_3B·NMeH_2$ containing dehydrogenation vessel was connected to the gas buret under an Ar atmosphere. The gas buret was initialised and $H_3B·NMeH_2$ and the catalyst were dissolved in 4 mL of THF or toluene and data acquisition was started immediately. After completion of the dehydrogenation reaction, a gas sample was taken and analysed by GC-TCD and an aliquot was analysed by $^{11}B/^{11}B\{^1H\}$ NMR spectroscopy. The reaction solution was cannula-transferred into an oven dried Schlenk flask under Ar flow. The polymer was obtained by precipitation into 50 mL of cold (-78 °C) *n*-hexane, allowed to precipitate for 30 minutes and subsequently filtered. The pale yellow solid was dried in vacuum overnight. Isolated yields varied from 10 - 70%.

Synthesis of *N*-trimethylsilylmethylamine borane, $H_3B·N(CH_2SiMe_3)H_2$ (4): An oven-dried Schlenk flask was charged with $H_3B·THF$ (20.4 mmol, 1 M in THF), cooled to -78 °C and trimethylsilylmethylamine $H_2NCH_2SiMe_3$ (2.0 g, 2.6 mL, 19.94 mmol) was added slowly to the solution via syringe. The cooling bath was removed and the solution was stirred for 3 h upon warming at room temperature. The solvent was removed in vacuum, the white crystalline residue was washed with 2 x 15 mL of *n*-hexane and dried in vacuum. The substrate was purified by sublimation (30 °C, 10^{-3} mbar) prior to use. Crystals suitable for X-ray analysis were obtained from cooling a saturated diethyl ether solution to -30°C.
Yield: 50-70%. Melting point: 56-58°C. ^1H NMR (300 MHz, $CDCl_3$, 22°C): δ 3.71 (br, 2H, NH_2), 2.28 (t, $^2J_{HH}$ = 6.7 Hz, 2H, CH_2), 1.86 (q br, J_{BH} = 80 Hz, J =110 Hz, 3H, BH_3), 0.11 (br, 9H, $(CH_3)_3Si$). ^{11}B NMR (96 MHz, $CDCl_3$, 22°C) δ -17.5 (br q, J_{BH} = 95 Hz). $^{13}C\{^1H\}$ NMR (101 MHz, $CDCl_3$, 22°C): δ 41.5 (br, CH_2), 0.0 (br, Me_3Si). ^{29}Si\{inept\} NMR (79 MHz, $CDCl_3$, 22°C): δ 0.6 (m). IR (ATR, 22 °C): $\tilde{\nu}$ 3248, 3226 (m, $\tilde{\nu}_{NH}$); 2955, 2915 (w, $\tilde{\nu}_{CH}$); 2378, 2306, 2265 (m, $\tilde{\nu}_{BH}$). Anal calcd. for $C_4H_{16}BNSi$ (%): C, 41.04, H, 13.78, N, 11.96. Found (%): C, 40.79, H, 14.04, N, 11.77. ESI-MS, calculated for $[C_4H_{13}NSi + H]^+$: 104.089. Found: 104.0893 $[M + H]^+$.

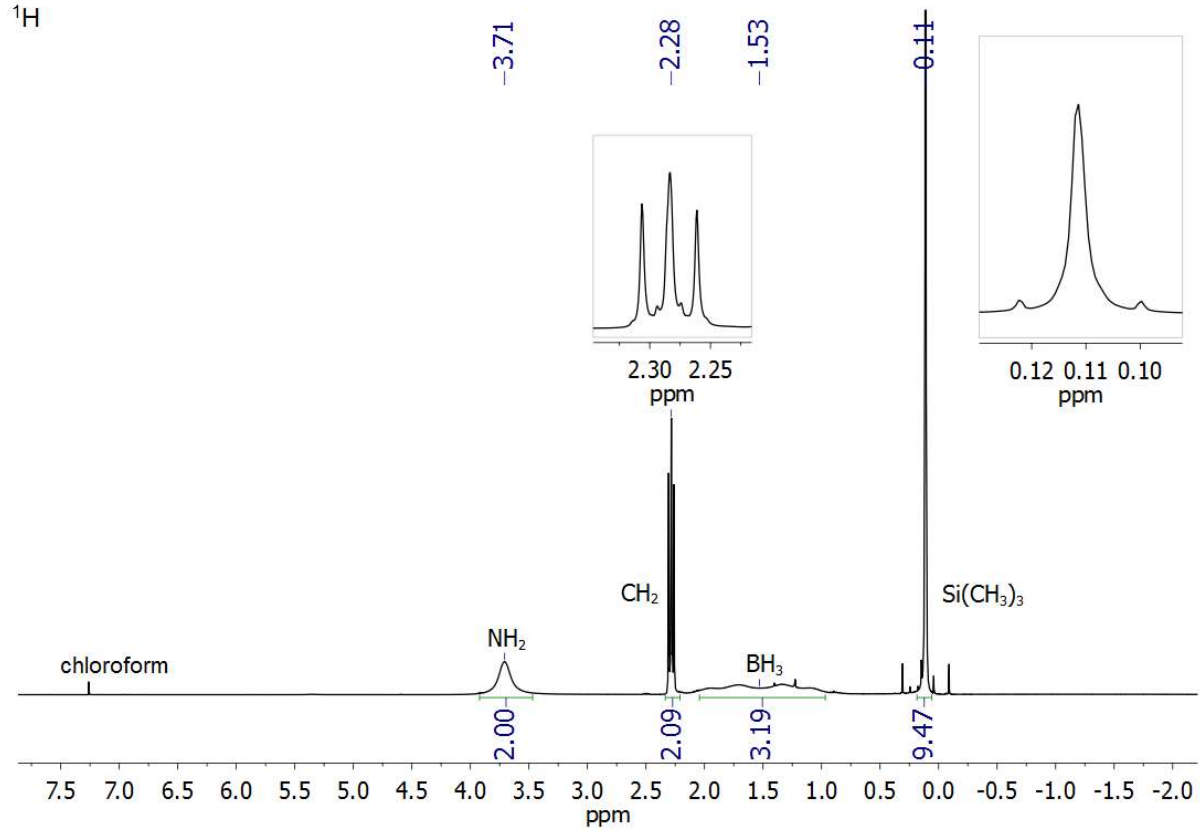

Figure S2. ^{1}H NMR spectrum (300 MHz, $CDCl_3$) of **4**.

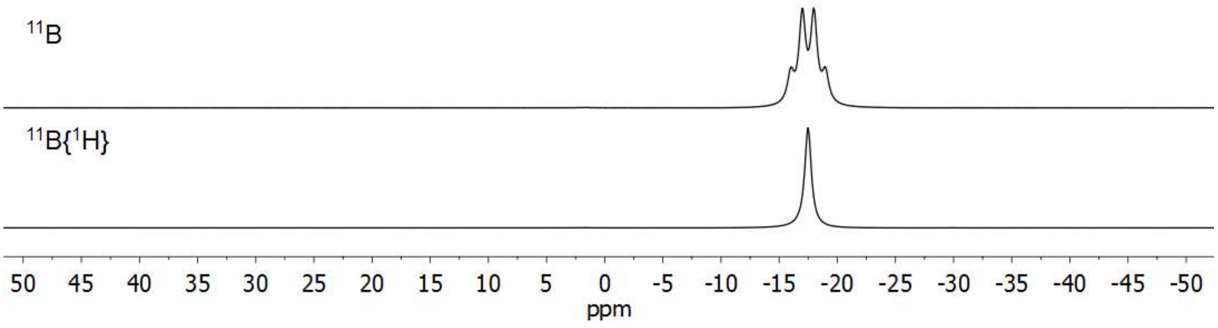

Figure S3. ^{11}B and $^{11}B\{^{1}H\}$ NMR spectra (96 MHz, $CDCl_3$) of **4**.

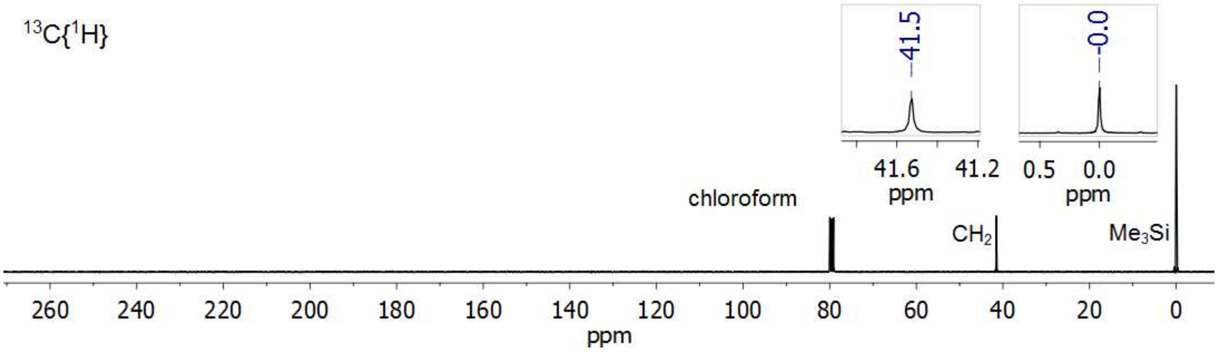

Figure S4. $^{13}C\{^{1}H\}$ NMR spectrum (101 MHz, $CDCl_3$) of **4**.

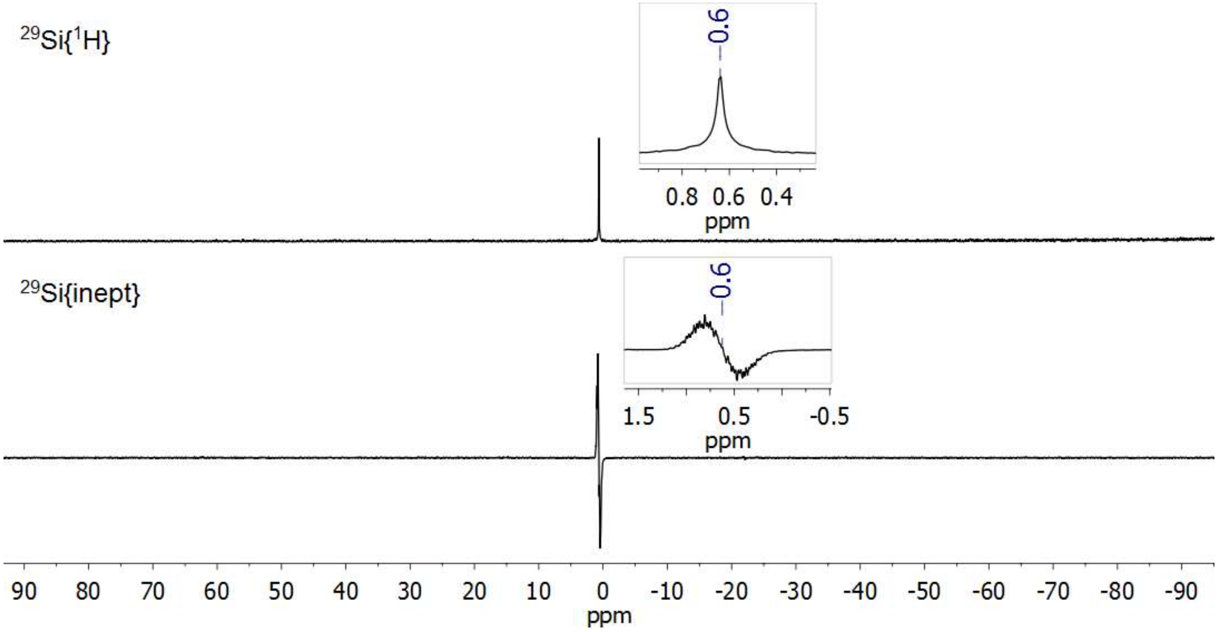

Figure S5. $^{29}Si\{^1H\}$ and ^{29}Si\{inept\} NMR spectra (79 MHz, $CDCl_3$) of **4**.

Synthesis of *N*-trimethylsilylmethyl-cyclotriborazane $[H_2BN(CH_2SiMe_3)H]_3$: The attempt to obtain the corresponding cyclic borazane from compound **4** was carried out in analogy to the synthesis of cyclic *N*-methyl-borazane reported by Vaultier *et al.*[16] The cyclic borazane was obtained by thermally induced dehydrogenation of **4** (500 mg, 4.27 mmol). The amine borane was heated to 120 °C within 30 minutes and kept at this temperature for one hour. During cooling, a white solid precipitated consisting of the desired cyclic borazane as well as unreacted **4** and *N*-trimethylsilylmethyl-borazine $[HB{=}NCH_2SiMe_3]_3$. Through sublimation at 70 °C at $1{\cdot}10^{-3}$ mbar the by-products were removed and pure cyclotriborazane remained as a white solid at the bottom of the flask. The product consists of two isomers.

^{1}H NMR (300 MHz, $CDCl_3$, 22°C): δ 2.35 (br s, NH), 2.21 (br s, NH), 2.00 (m, CH_2), 1.71 (br s, NH), 0.11, 0.10, 0.09 (s, $(CH_3)_3Si$), between 2.4 and 1.4 (br overlapped, BH_2). ^{11}B{^{1}H} NMR (96 MHz, $CDCl_3$, 22°C): δ -3.8 (s), -4.4 (s). ^{13}C{^{1}H} NMR (101 MHz, $CDCl_3$, 22°C) δ 41.4, 41.2, 39.7(s, CH_2), -1.1, -1.3, -1.4, (s, Me_3Si). ^{29}Si{inept} NMR (79 MHz, $CDCl_3$, 22°C) δ 1.1 (m), 0.4 (m). ^{29}Si{^{1}H} NMR (79 MHz, $CDCl_3$, 22°C) δ 1.0 (s), 0.6 (s), 0.3 (s). ESI-MS, calculated for $[C_8H_{26}BN_2Si_2]^+$: 217.17.Found: 217.1728 [M, - BH_2 $(NH(CH_2SiCH_3))_2]^+$. Calculated for $[C_{11}H_{39}B_3N_3Si_3]^+$: 330.27. Found: 330.2571 [M, - $CH_3]^+$.

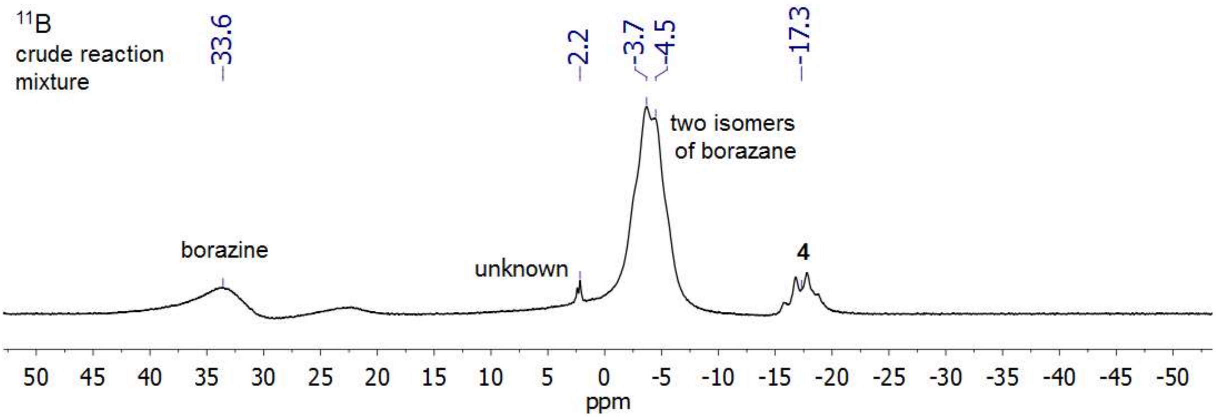

Figure S6. ^{11}B NMR (96 MHz, $CDCl_3$) spectrum of the crude reaction mixture before purification.

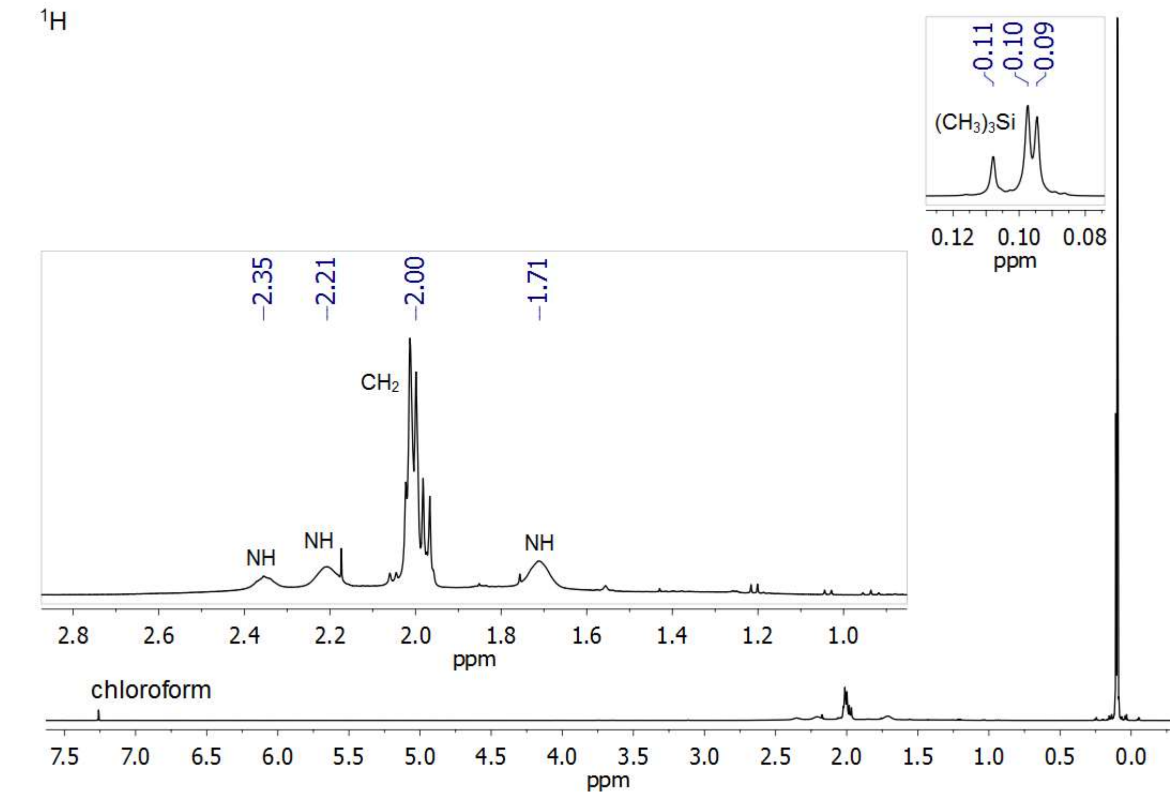

Figure S7. ^{1}H NMR spectrum (300 MHz, $CDCl_3$) of the two isomers of $[H_2BN(CH_2SiMe_3)H]_3$ after sublimation. The broad signal for BH_2 is not visible.

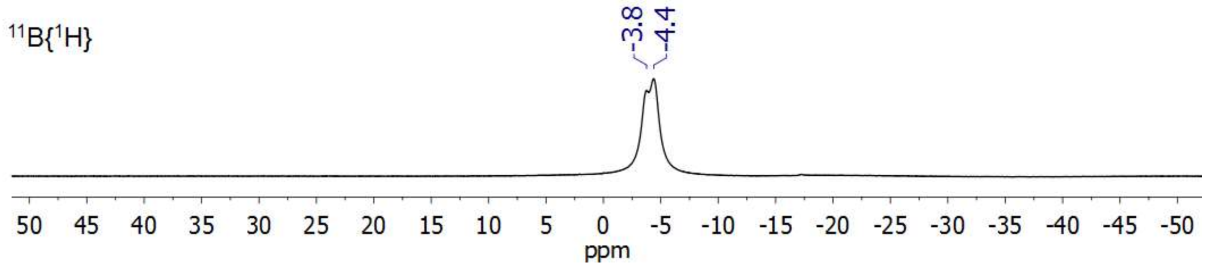

Figure S8. $^{11}B\{^{1}H\}$ NMR spectrum (96 MHz, $CDCl_3$) of the two isomers of $[H_2BN(CH_2SiMe_3)H]_3$ after sublimation.

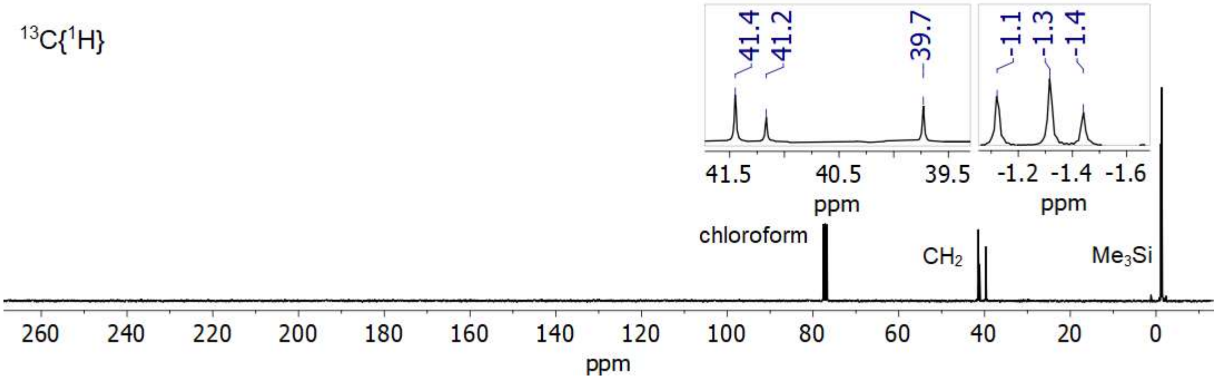

Figure S9. $^{13}C\{^{1}H\}$ NMR spectrum (101 MHz, $CDCl_3$) of the two isomers of $[H_2BN(CH_2SiMe_3)H]_3$ after sublimation.

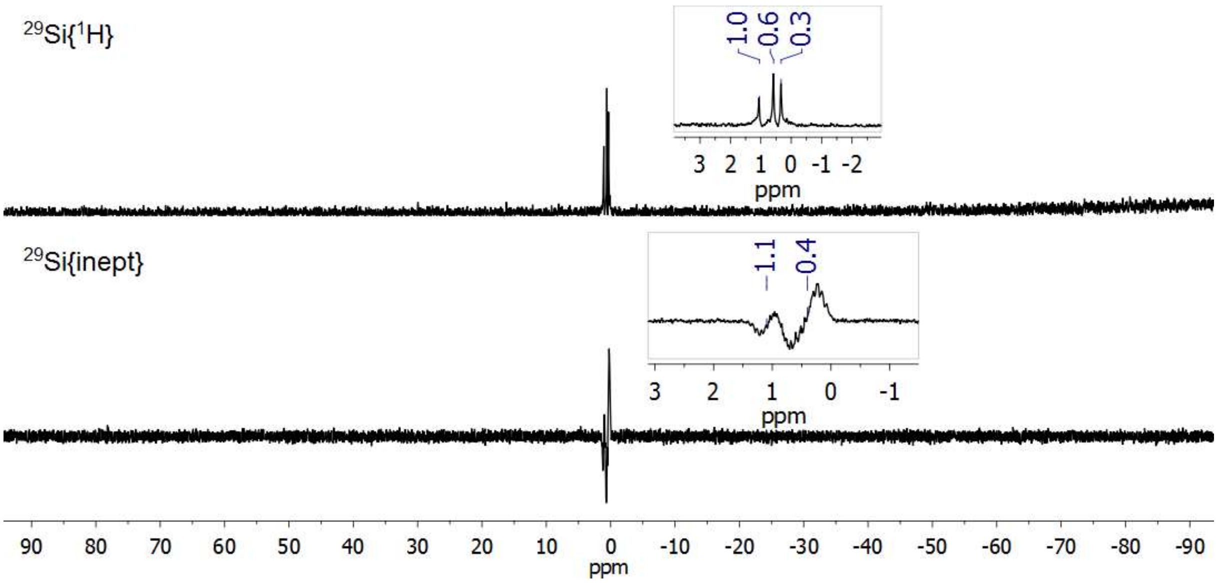

Figure S10. ^{29}Si{^{1}H}, ^{29}Si{inept} NMR spectra (79 MHz, $CDCl_3$) of the two isomers of $[H_2BN(CH_2SiMe_3)H]_3$ after sublimation.

Catalytic dehydropolymerisation of $H_3B·NMeH_2$

Catalytic dehydropolymerisation of $H_3B·NMeH_2$ using complex 1

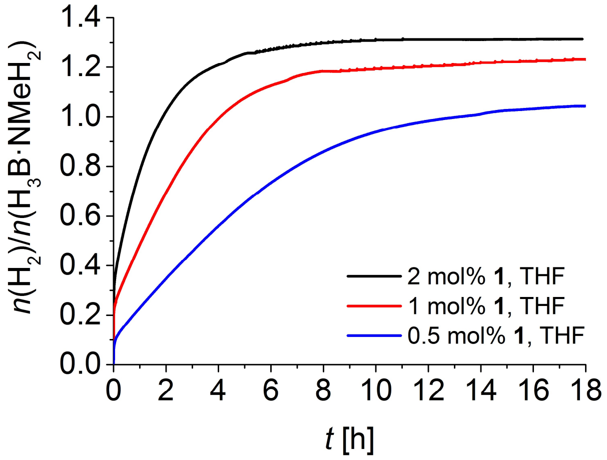

Figure S11. Volumetric curves of $H_3B·NMeH_2$ dehydropolymerisation using complex **1** in THF at T = 25 °C and $[H_3B·NMeH_2]$ = 0.33 M.

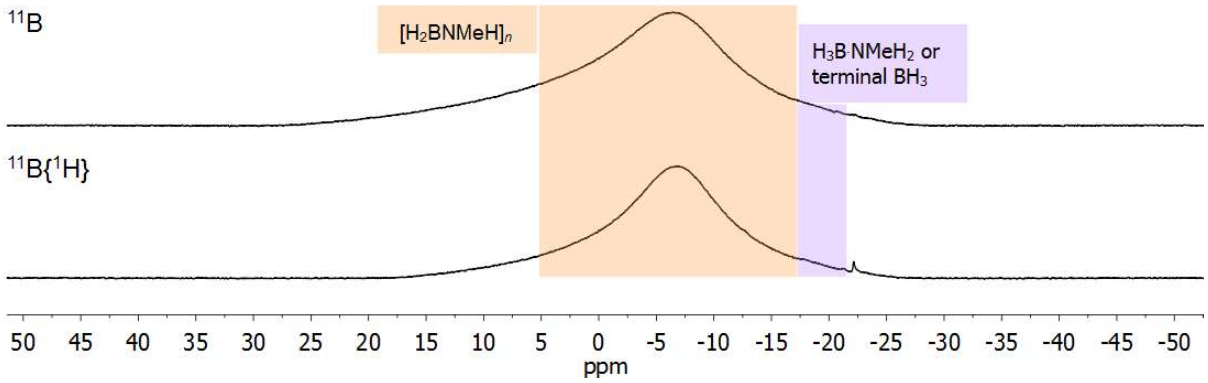

Figure S12. ^{11}B, $^{11}B\{^1H\}$ NMR (96 MHz, $CDCl_3$) spectra of the isolated $[H_2BNMeH]_n$ from H_3BNMeH_2 dehydropolymerisation using complex **1** (2 mol%) in THF at T = 25 °C and $[H_3B·NMeH_2]$ = 0.33 M.

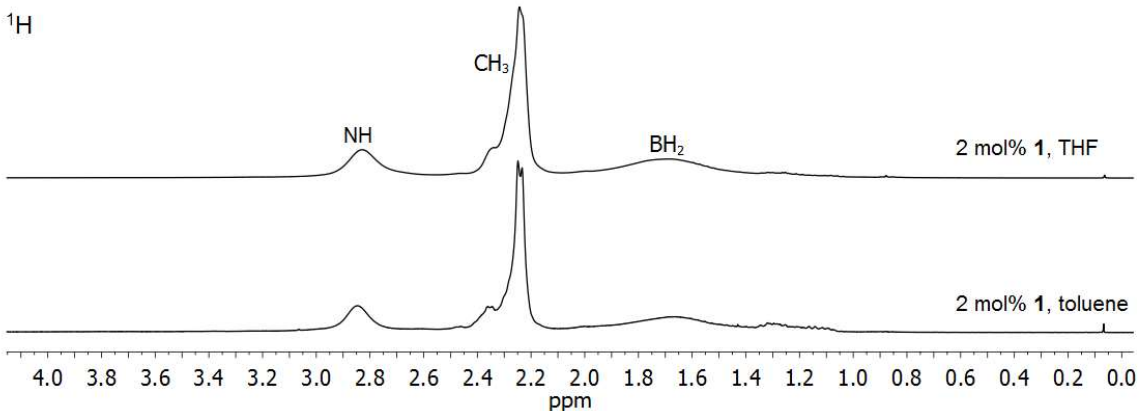

Figure S13. ^1H NMR (300 MHz, $CDCl_3$) spectra of the isolated $[H_2BNMeH]_n$ from the H_3BNMeH_2 dehydropolymerisation using complex **1** (2 mol%) in THF and toluene at T = 25 °C and $[H_3B·NMeH_2]$ = 0.33 M.

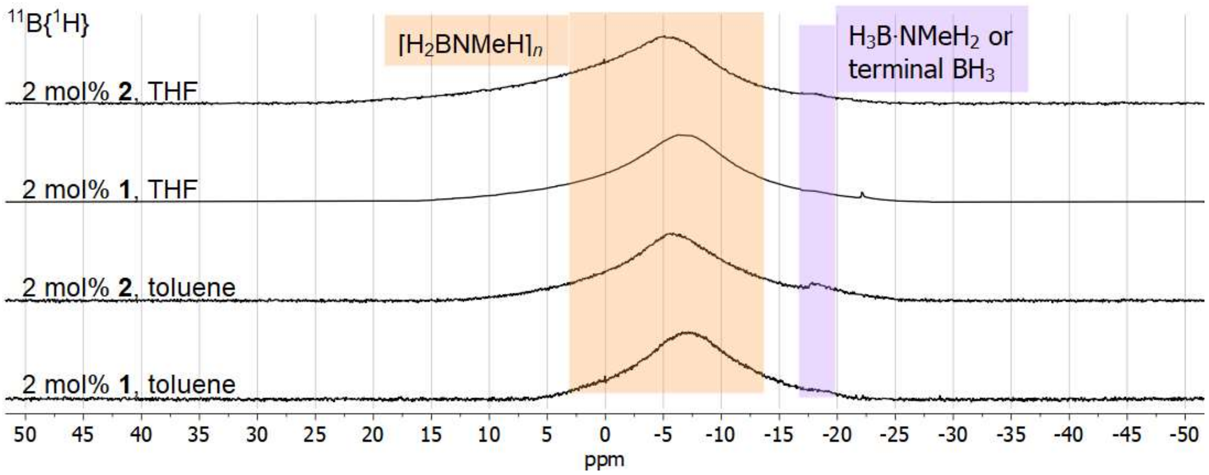

Figure S14. $^{11}B\{^{1}H\}$ NMR (96 MHz, $CDCl_3$) spectra of the isolated $[H_2BNMeH]_n$ from $H_3B{\cdot}NMeH_2$ dehydropolymerisation at T = 25 °C and $[H_3B{\cdot}NMeH_2]$ = 0.33 M. The BH_2 resonance is slightly shifted when using different iron catalysts.

Monitoring the dehydropolymerisation of $H_3B{\cdot}NMeH_2$ using complex 1

Reactions in THF: The reaction was carried out in an oven dried Schlenk flask containing Fe catalyst (15.6 mg, 0.04 mmol, 2 mol%) and $H_3B{\cdot}NMeH_2$ (90 mg, 2 mmol) in 6 mL of THF. The flask was connected to a bubbler. After reaction, an aliquot of 0.4 mL was taken from the solution and transferred into an NMR tube for NMR analysis.

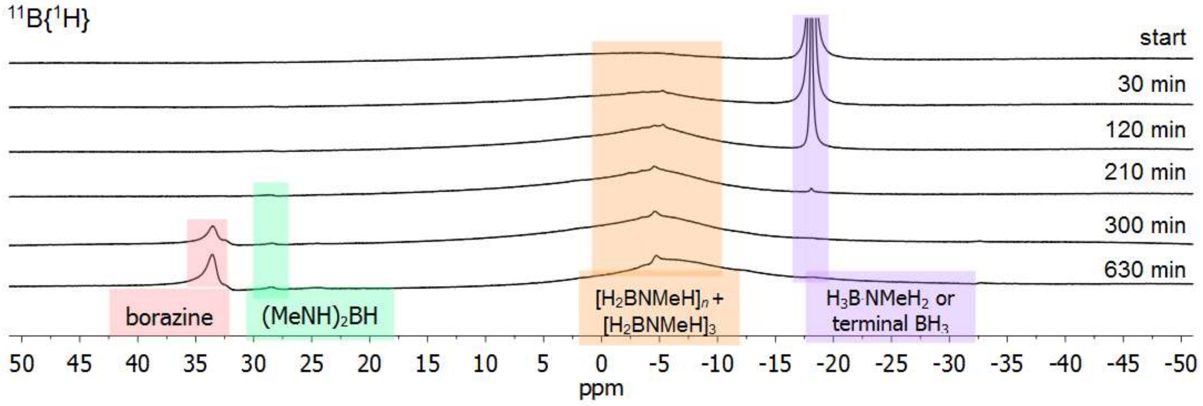

Figure S15. *In situ* $^{11}B\{^1H\}$ NMR (96 MHz, THF-d_8) spectra recorded during dehydropolymerisation of $H_3B{\cdot}NMeH_2$ using complex **1** in THF at T = 25 °C and [$H_3B{\cdot}NMeH_2$] = 0.33 M.

Reactions in toluene: The reaction was carried out in an oven dried Schlenk flask containing Fe catalyst (3.9 mg, 0.01 mmol, 0.5 mol%) and $H_3B{\cdot}NMeH_2$ (90 mg, 2 mmol) in 6 mL of toluene. The flask was connected to a bubbler. After reaction, an aliquot of 0.4 mL was taken from the solution and transferred into an NMR tube for NMR analysis.

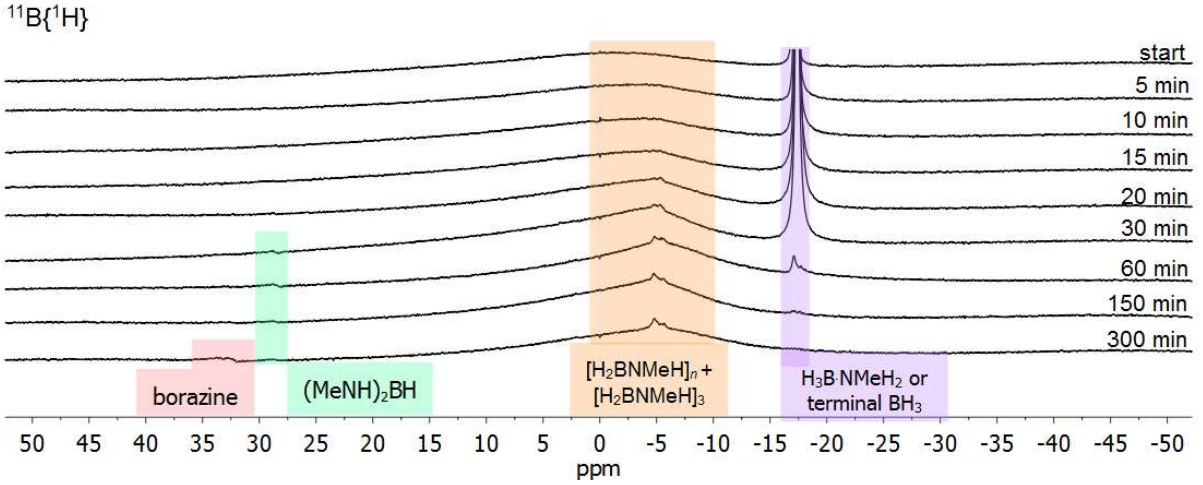

Figure S16. *In situ* $^{11}B\{^1H\}$ NMR (96 MHz, toluene-d_8) spectra recorded during dehydropolymerisation of $H_3B{\cdot}NMeH_2$ using complex **1** in toluene at T = 25 °C and [$H_3B{\cdot}NMeH_2$] = 0.33 M.

Kinetic analysis

As can be seen from the figures below, classical kinetic analysis using linearisations of the integrated first-order rate law give significant deviations from ideal linear behaviour, especially for low concentrations of the Fe catalyst (Figure S17). Generally, such systematic deviations can be assigned to systematic errors in $[S]_0$ (in this case $[H_3B{\cdot}NMeH_2]_0$), caused by wrong weighing (problems with transfer of chemicals).
For this reason, we have investigated our experimental volumetric data using non-linear regression analysis. Examples are shown below. Values determined from this analysis are given in Table 1 and Table S1. Analysis using non-linear regression (first-order) fits well for dehydrogenation reactions at high catalyst concentration (Figure S18, left, see R.M.S. and standard deviation values). When going to lower catalyst-to-substrate ratio and high substrate concentrations (e.g. 0.5 mol% Fe, $[H_3B{\cdot}NMeH_2]_0$ = 0.33 M) the calculated data results are less accurate (Figure S19, left) according to first-order evaluation. Using non-linear regression in a Michaelis-Menten approach seems to be the best model to describe the system in these cases (Figure S19, right).
It should be noted that due to poor solubility of $H_3B{\cdot}NMeH_2$ in toluene we were not able to reach the saturation range (v_{max}). In our case the standard reaction conditions (0.5 mol% Fe,$[H_3B{\cdot}NMeH_2]_0$ = 0.33 M) correspond to a range $v{\approx}v_{max}/2$.

First-order plots

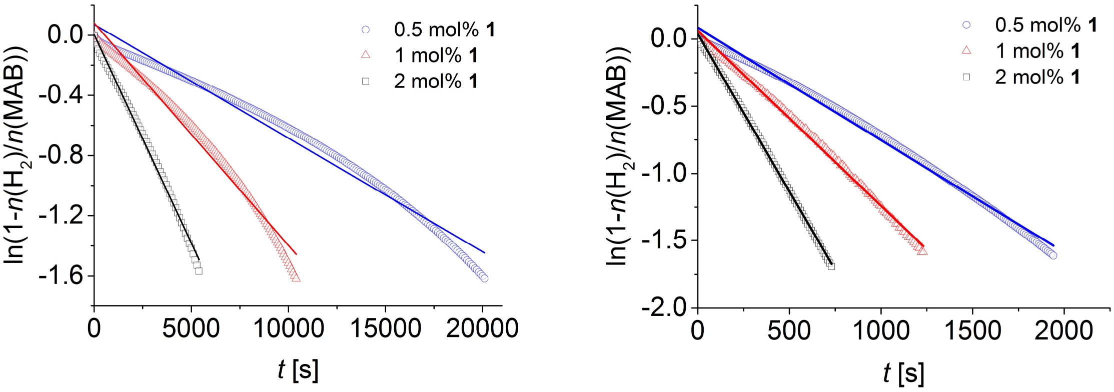

Figure S17. First-order plot of $H_3B{\cdot}NMeH_2$ dehydropolymerisation using complex **1** in THF (left) and in toluene (right) at T = 25°C and $[H_3B{\cdot}NMeH_2]$ = 0.33 M. Both graphics show that for less than 2 mol% of Fe complex significant deviations from first-order behaviour are found.

Comparison of first-order and Michaelis-Menten model, toluene

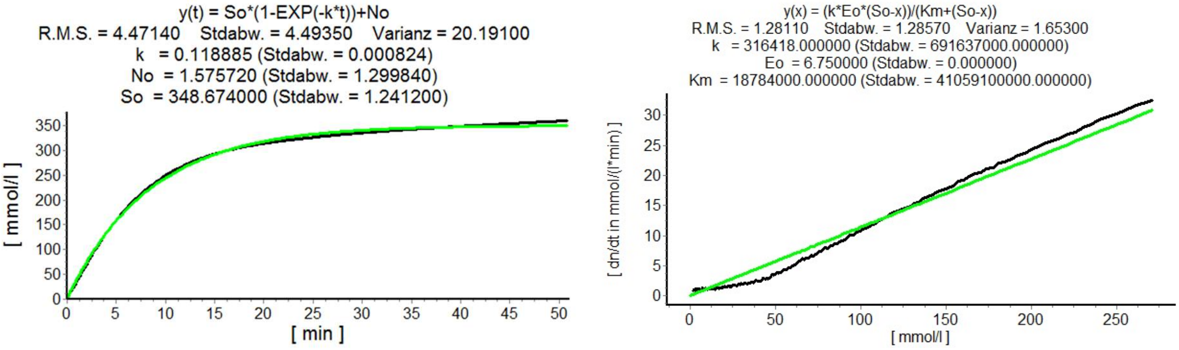

Figure S18. Left: First-order plot of experimental (black) and fitted (green) data using complex **1** (2 mol%). Right: Michaelis-Menten plot of experimental (black) and fitted (green) data using complex **1** (2 mol%). Conditions: toluene at T = 25 °C and $[H_3B{\cdot}NMeH_2]$ = 0.33 M.

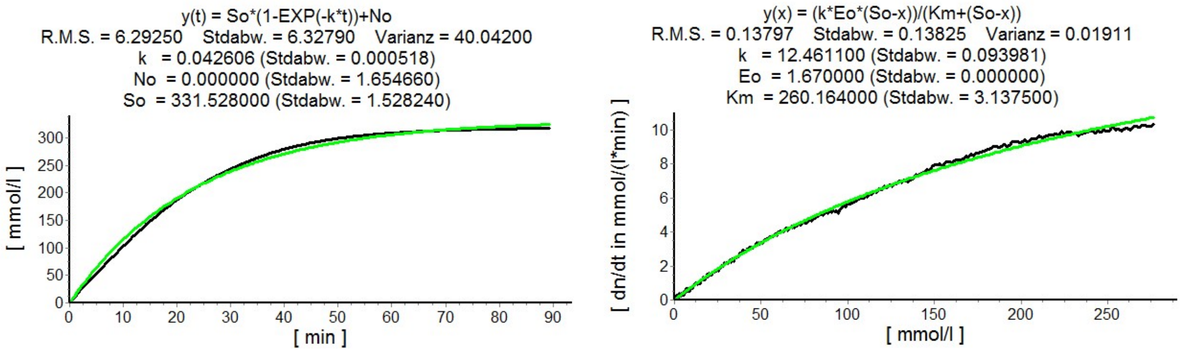

Figure S19. Left: First-order plot of experimental (black) and fitted (green) data using complex **1** (0.5 mol%). Right: Michaelis-Menten plot of experimental (black) and fitted (green) data using complex **1** (0.5 mol%). Conditions: toluene at T = 25 °C and [$H_3B{\cdot}NMeH_2$] = 0.33 M.

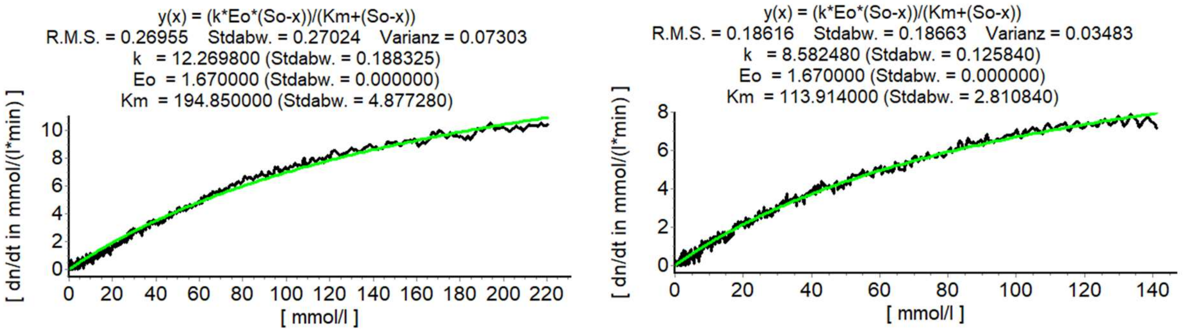

Figure S20. Left: Michaelis-Menten plot of experimental (black) and fitted (green) data using [$H_3B{\cdot}NMeH_2$] = 0.25 M. Right: Michaelis-Menten plot of experimental (black) and fitted (green) data using [$H_3B{\cdot}NMeH_2$] = 0.17 M. Conditions: complex **1** (0.00167 M) and toluene at T = 25 °C.

Comparison of first-order and Michaelis-Menten model, THF

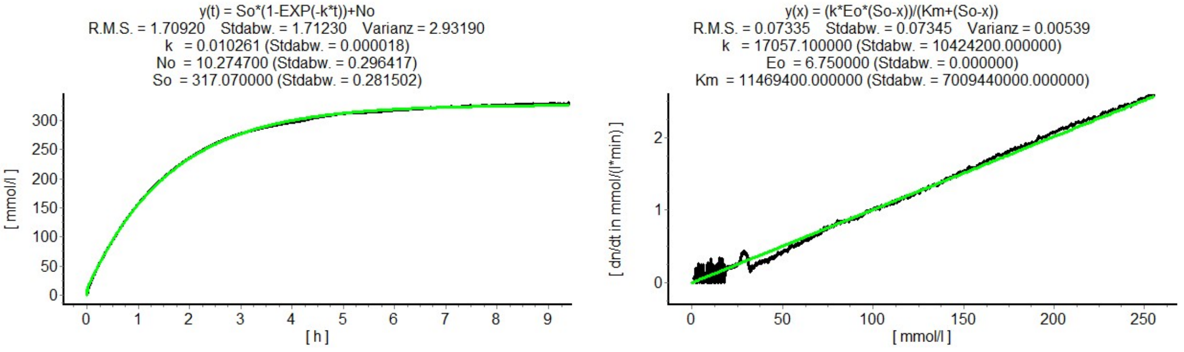

Figure S21. Left: First-order plot of experimental (black) and fitted (green) data using complex **1** (2 mol%). Right: Michaelis-Menten plot of experimental (black) and fitted (green) data using complex **1** (2 mol%). Conditions: THF at T = 25 °C and [$H_3B{\cdot}NMeH_2$] = 0.33 M.

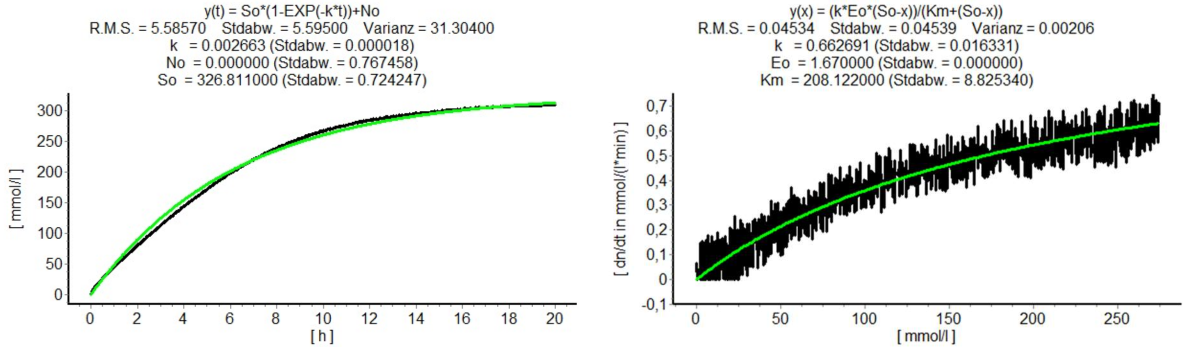

Figure S22. Left: First-order plot of experimental (black) and fitted (green) data using complex **1** (0.5 mol%). Right: Michaelis-Menten plot of experimental (black) and fitted (green) data using complex **1** (0.5 mol%). Conditions: THF at T = 25 °C and [$H_3B{\cdot}NMeH_2$] = 0.33 M.

Variable Time Normalisation Analysis (VTNA)[17,18]

Plotting of the reaction profiles in toluene and THF against $t \cdot [\mathbf{1}]^x$ shows a good visual overlay for the power value/reaction order x = 1 in both cases (Figure S23). It should however be noted that slight deviations are observed for low catalyst concentrations. We believe that this indicates differences in the overall kinetic regime of the reaction which can be regarded as a limiting case of first-order/Michaelis-Menten kinetics.

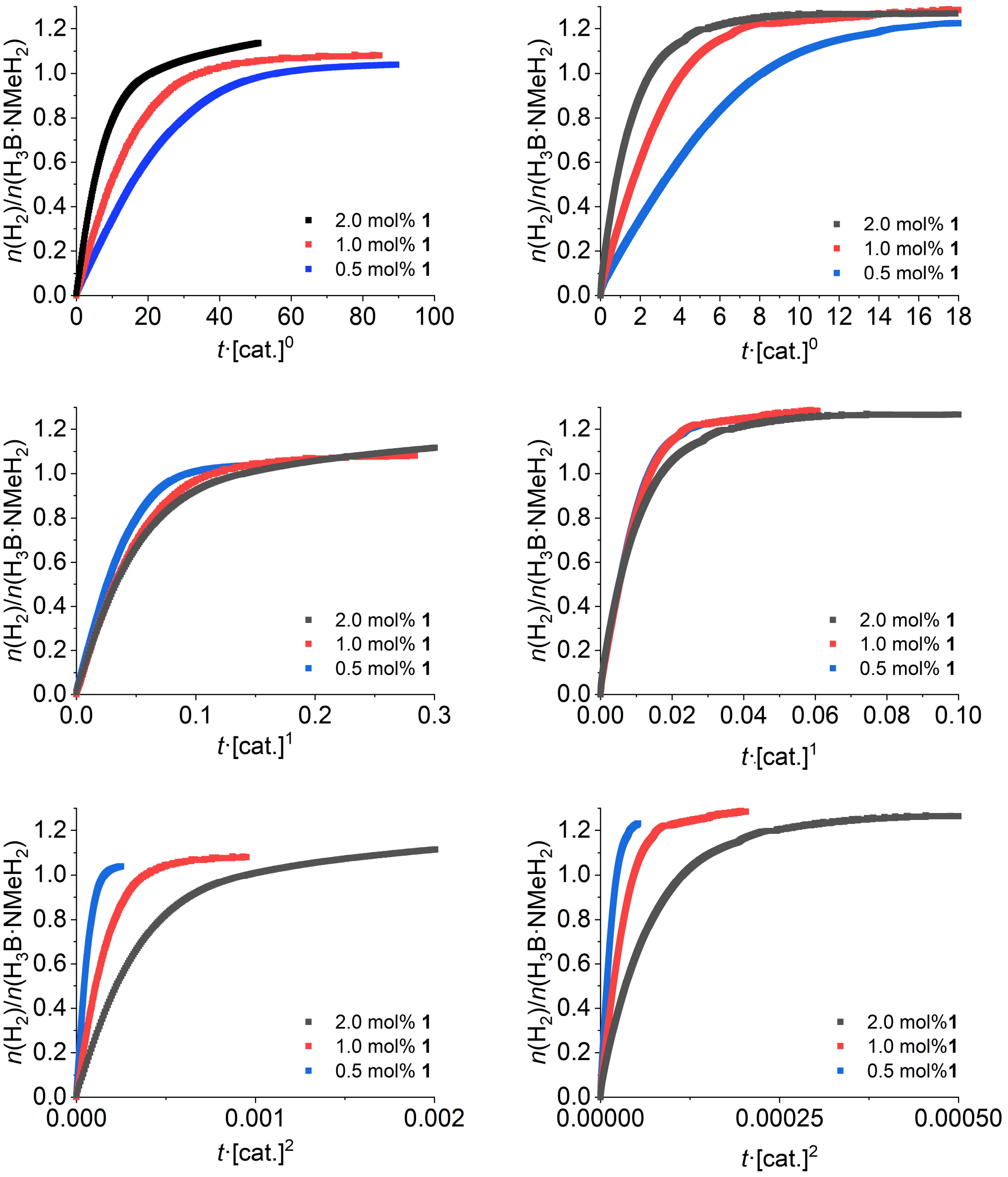

Figure S23. VTNA plots for reactions in toluene (left) and THF (right).

Table S1. Compilation of further kinetic data of amine borane dehydropolymerisation using complex **1**.

Entry	Reaction conditions [a]	k_{obs} [min^{-1}]	K_M [mmol/mL]	k_2 [min^{-1}]
1	[**1**] = 0.00675 M, THF, $H_3B{\cdot}NMeH_2$	$1.026(2)\cdot10^{-2}$ [b]		
2	[**1**] = 0.00335 M, THF, $H_3B{\cdot}NMeH_2$	$1.48(3)\cdot10^{-2}$ [c]	$1.80(3)\cdot10^{-1}$	$7.99(6)\cdot10^{-1}$
3	[**1**] = 0.00167 M, THF, $H_3B{\cdot}NMeH_2$	$5.3(4)\cdot10^{-2}$ [c]	$2.08(9)\cdot10^{-1}$	$6.6(2)\cdot10^{-1}$
4	[**1**] = 0.00167 M, toluene, $H_3B{\cdot}NMe_2H$	$1.768(4)\cdot10^{-2}$ [b]		

[a] T = 25 °C, [amine borane] = 0.33 M. [b] According to first-order model. [c] Calculated according to Michaelis-Menten model: $k_{obs} = (k_2{\cdot}E_0)/K_M$.

Catalytic dehydropolymerisation with deuterated amine boranes adducts (KIE)

$H_3B{\cdot}NMeD_2$ was prepared according to a literature procedure.[19] $D_3B{\cdot}NMeH_2$ was prepared from $D_3B{\cdot}NMe_3$ according to a literature procedure.[20] $D_3B{\cdot}NMe_3$ was prepared by deuteration of $H_3B{\cdot}NMe_3$ (500 mg, 6.85 mmol) in THF (25 mL) using [(dppp)Rh(nbd)][BF_4] (57 mg, 1.5 mol%) as the catalyst. Deuteration was done at room temperature under 30 bar D_2, followed by transfer of the solution into an oven-dried Schlenk flask and removal of THF under a stream of Ar at room temperature. The solid was sublimed under static vacuum at room temperature to yield pure $D_3B{\cdot}NMe_3$ (220 mg, 44%). Spectroscopic data are in accordance with the literature.[19]

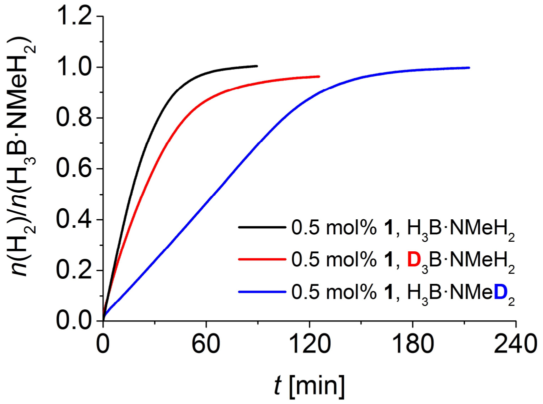

Figure S24. Volumetric curves of dehydropolymerisation of deuterated $R_3B{\cdot}NMeR_2$ (R = H or D) using complex **1** in toluene at T = 25 °C and [$R_3B{\cdot}NMeR_2$] = 0.33 M.

Poisoning experiments

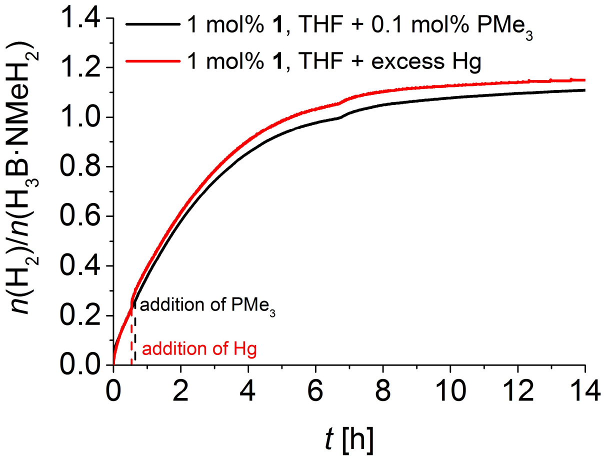

Figure S25. Volumetric curves of $H_3B{\cdot}NMeH_2$ dehydropolymerisation using complex **1** in THF (left) and toluene (right) in presence of poisoning reagents PMe_3 and mercury at T = 25 °C and [$H_3B{\cdot}NMeH_2$] = 0.33 M.

Low-temperature dehydropolymerisation of $H_3B{\cdot}NMeH_2$ using complex 1

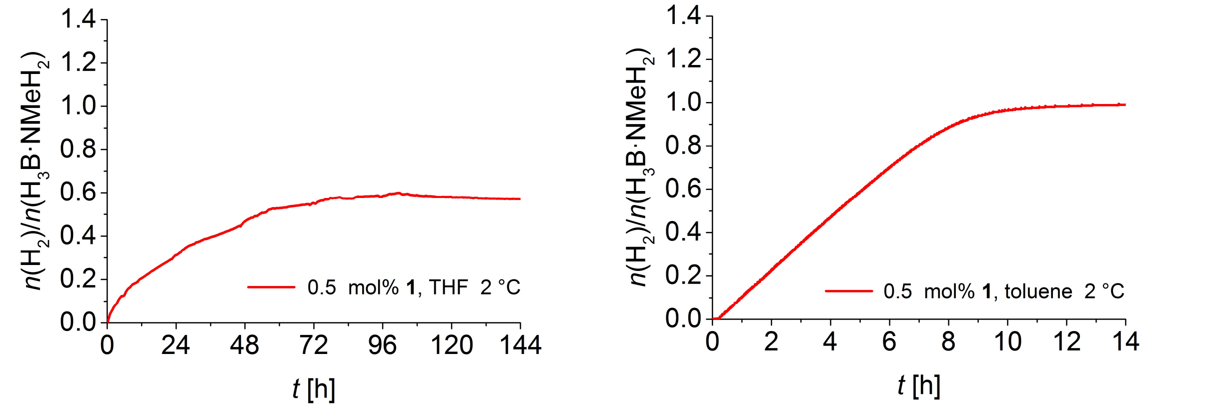

Figure S26. Volumetric curves of $H_3B{\cdot}NMeH_2$ dehydropolymerisation using complex **1** in THF (left) and toluene (right) at T = 2 °C and $[H_3B{\cdot}NMeH_2]$ = 0.33 M.

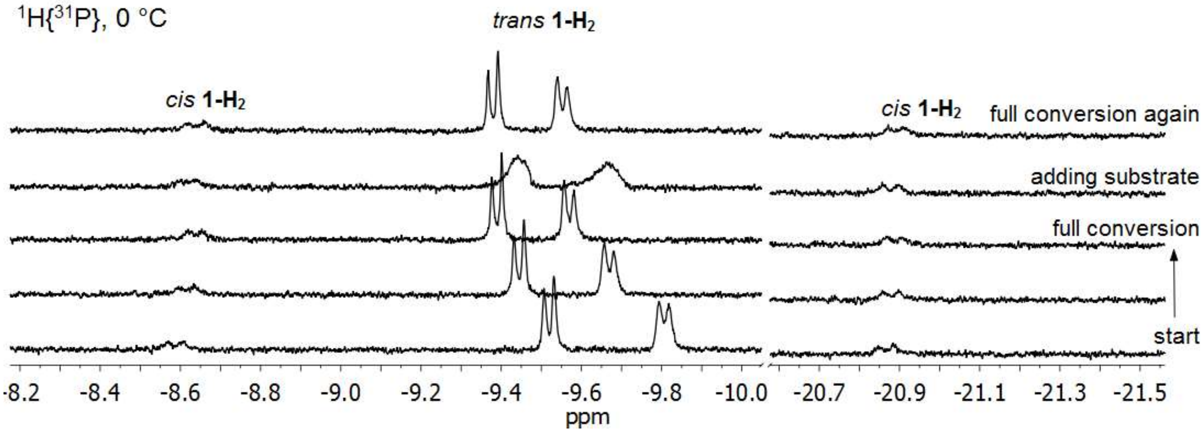

Figure S27. *In situ* $^{1}H\{^{31}P\}$ NMR spectra (400 MHz, 0°C) of $H_3B{\cdot}NMeH_2$ dehydropolymerisation using complex **1** (0.5 mol%) in toluene-d_8 at T = 0 °C and $[H_3B{\cdot}NMeH_2]$ = 0.33 M.

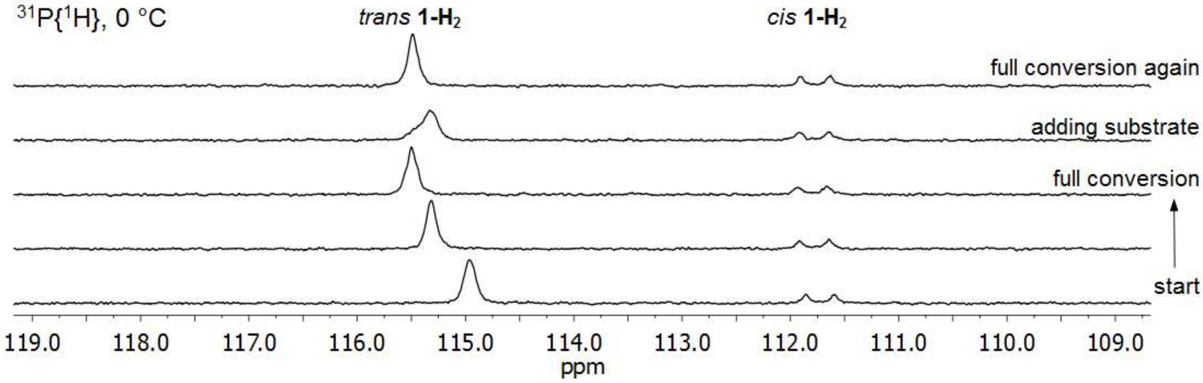

Figure S28. *In situ* $^{31}P\{^{1}H\}$ NMR spectra (400 MHz, 0°C) of $H_3B{\cdot}NMeH_2$ dehydropolymerisation using complex **1** (0.5 mol%) in toluene-d_8 at T = 0 °C and $[H_3B{\cdot}NMeH_2]$ = 0.33 M.

Catalytic dehydropolymerisation of $H_3B{\cdot}NMeH_2$ using complex 1-BH3

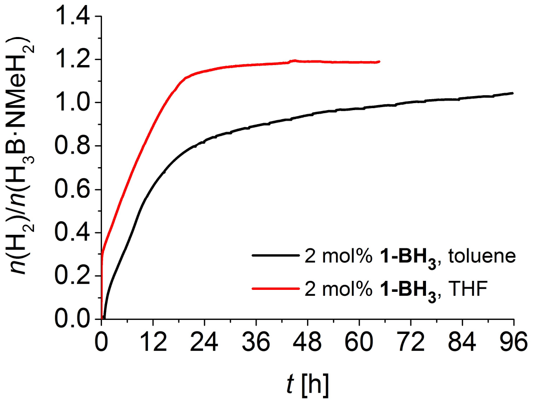

Figure S29. Volumetric curves of $H_3B{\cdot}NMeH_2$ dehydropolymerisation using complex **1-BH3** in THF (red) and toluene (black) at T = 25 °C and [$H_3B{\cdot}NMeH_2$] = 0.33 M.

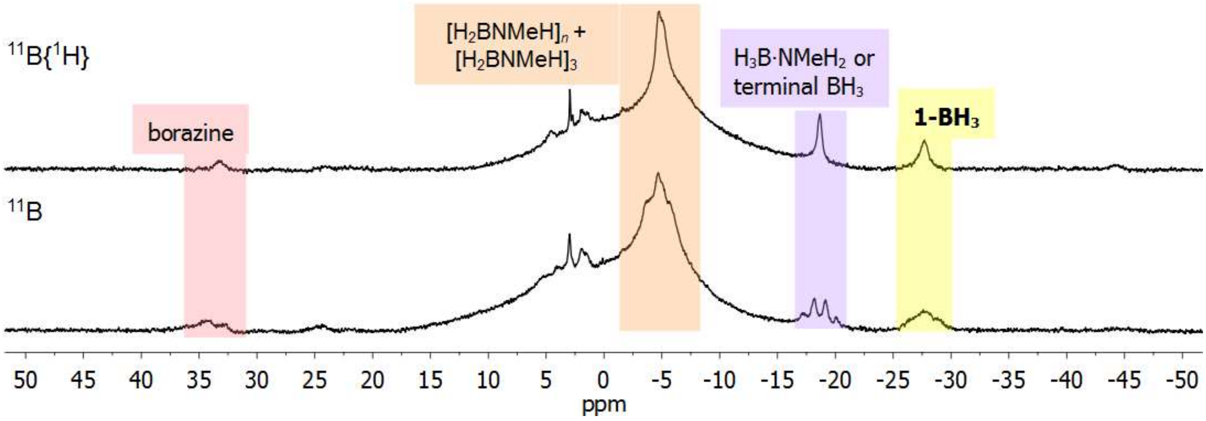

Figure S30. *In situ* $^{11}B\{^1H\}$ and ^{11}B NMR spectra (96 MHz, $CDCl_3$) recorded at the end of $H_3B{\cdot}NMe_2H$ dehydropolymerisation using complex **1-BH3** (2 mol%) in THF at T = 25 °C and [$H_3B{\cdot}NMeH_2$] = 0.33 M.

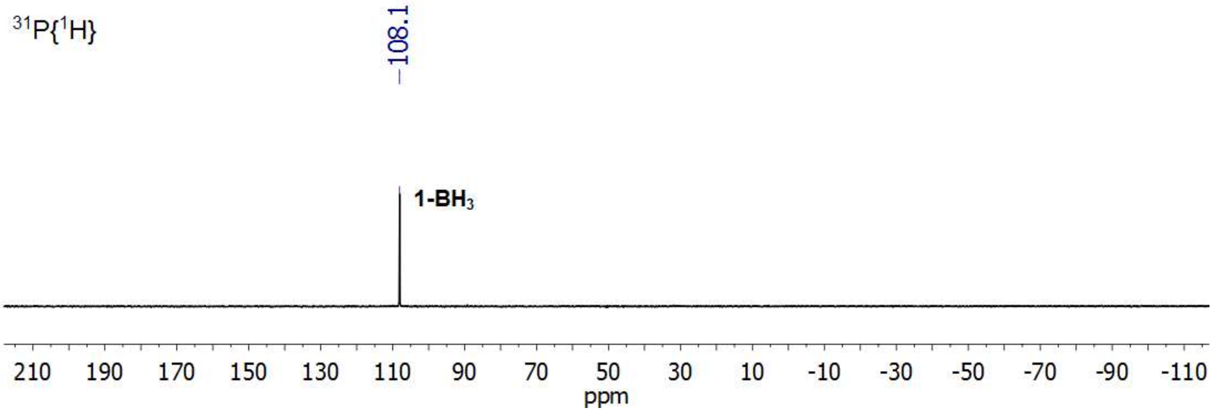

Figure S31. *In situ* ^{31}P NMR spectrum (121 MHz, $CDCl_3$) recorded at the end of $H_3B{\cdot}NMe_2H$ dehydropolymerisation using complex **1-BH3** (2 mol%) in THF at T = 25 °C and [$H_3B{\cdot}NMeH_2$] = 0.33 M.

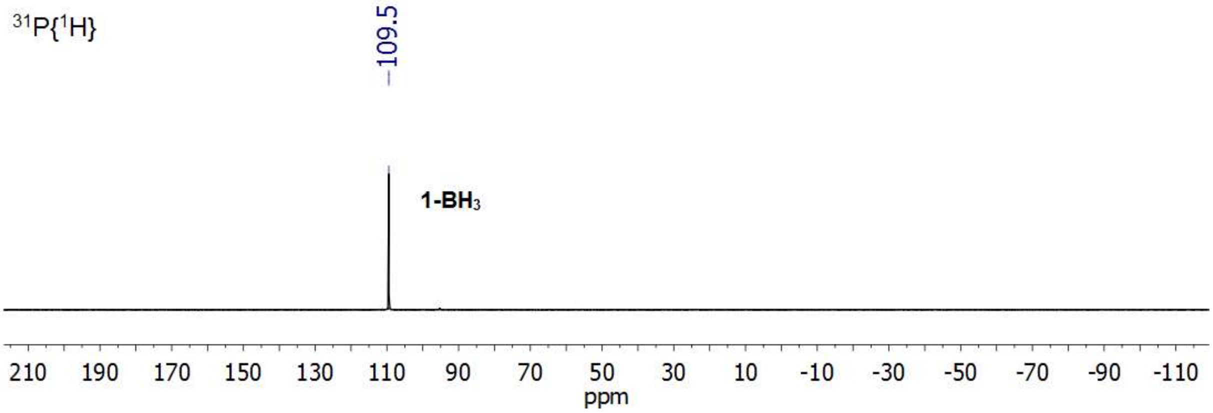

Figure S32. *In situ* ^{31}P NMR spectrum (121 MHz, $CDCl_3$) recorded at the end of $H_3B{\cdot}NMe_2H$ dehydropolymerisation using complex **1-BH$_3$** (2 mol%) in toluene at T = 25 °C and [$H_3B{\cdot}NMeH_2$] = 0.33 M.

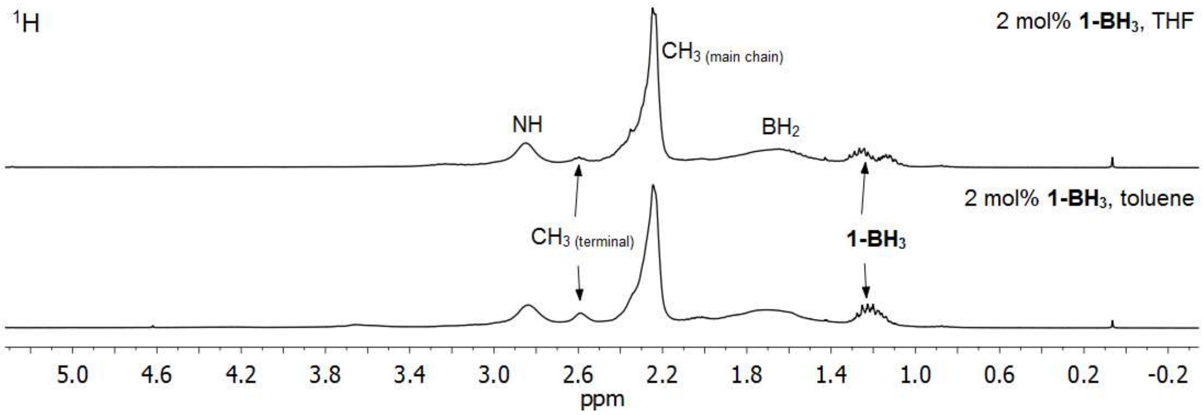

Figure S33. ^{1}H NMR spectra (300 MHz, $CDCl_3$) of the isolated [H_2BNMeH]$_n$ from H_3BNMeH_2 dehydropolymerisation using complex **1-BH$_3$** (2 mol%) in THF and toluene at T = 25 °C and [$H_3B{\cdot}NMeH_2$] = 0.33 M.

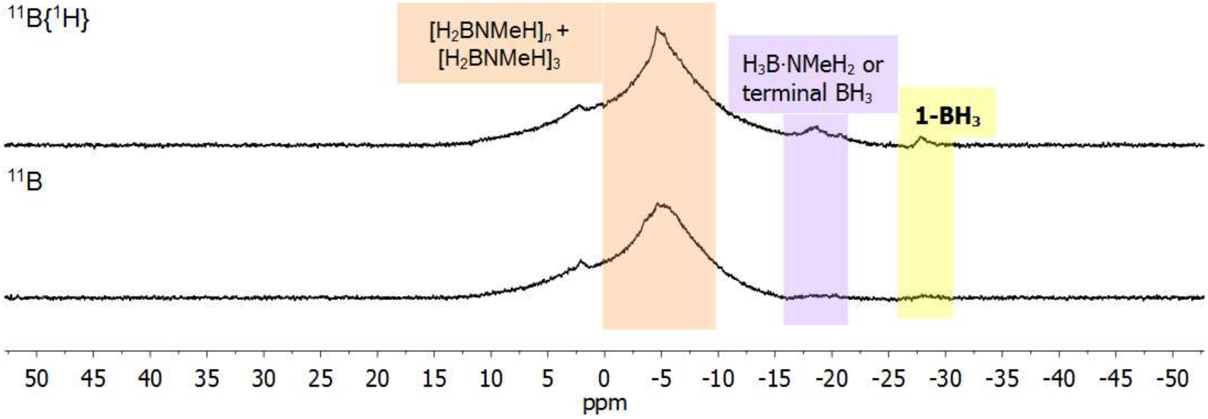

Figure S34. ^{11}B and ^{11}B{^{1}H} NMR spectra (96 MHz, $CDCl_3$) of the isolated [H_2BNMeH]$_n$ from H_3BNMeH_2 dehydropolymerisation using complex **1-BH$_3$** (2 mol%) in THF at T = 25 °C and [$H_3B{\cdot}NMeH_2$] = 0.33 M.

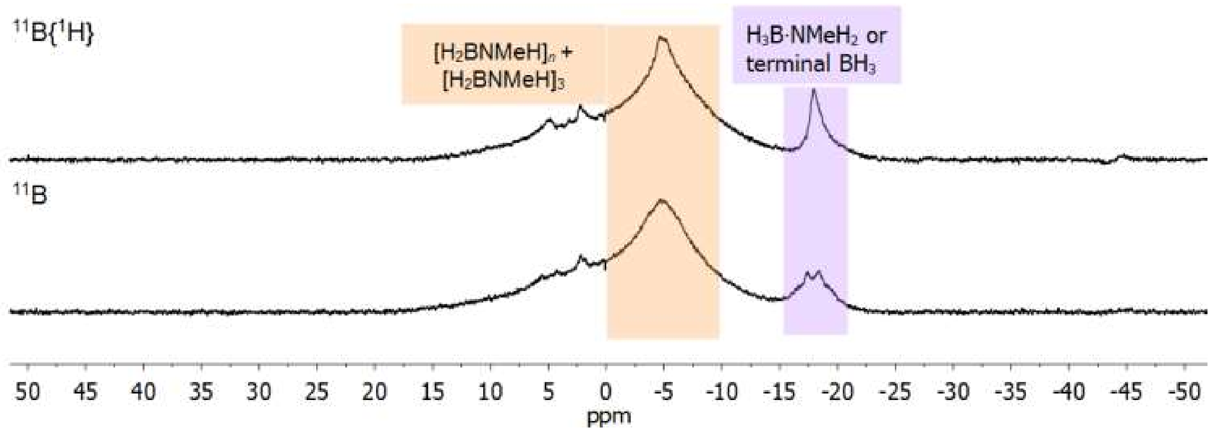

Figure S 35. ^{11}B and $^{11}B\{^1H\}$ NMR spectra (96 MHz, $CDCl_3$) of the isolated $[H_2BNMeH]_n$ from H_3BNMeH_2 dehydropolymerisation using complex **1-BH₃** (2 mol%) in toluene at T = 25 °C and $[H_3B{\cdot}NMeH_2]$ = 0.33 M.

Catalytic dehydropolymerisation of $H_3B{\cdot}NMeH_2$ using complex 3

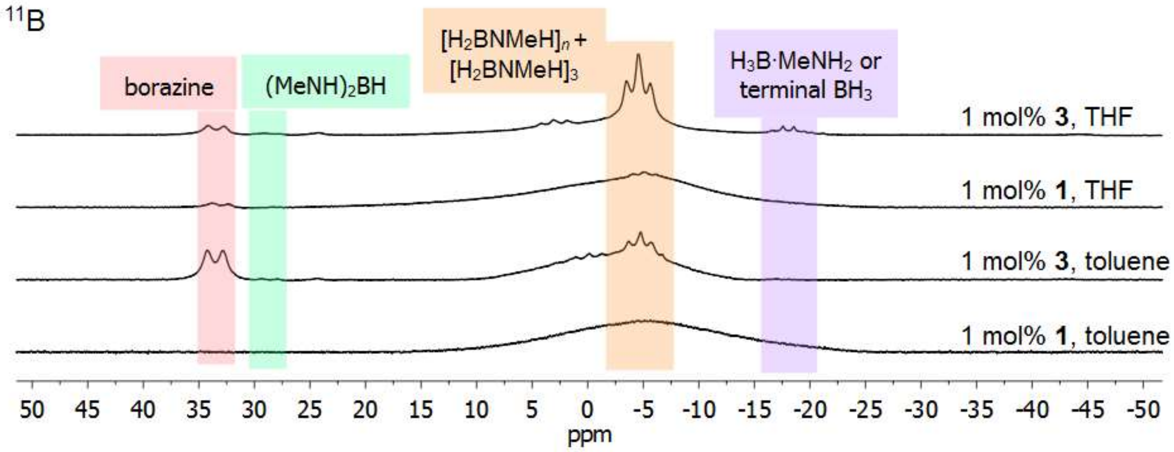

Figure S36. *In situ* ^{11}B NMR spectra (96 MHz, 1024 scans) recorded at the end of $H_3B{\cdot}NMe_2H$ dehydropolymerisation using complexes **1** and **3** in THF and toluene at T = 25 °C and $[H_3B{\cdot}NMeH_2]$ = 0.33 M.

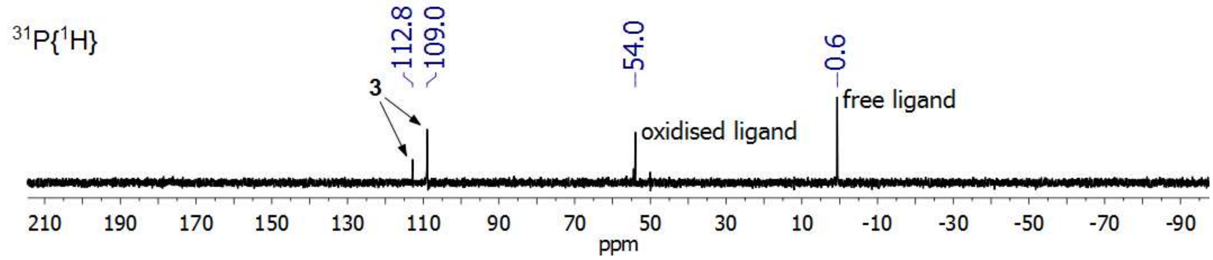

Figure S37. *In situ* ^{31}P NMR spectrum (121 MHz, THF-d_8) recorded at the end of $H_3B{\cdot}NMe_2H$ dehydropolymerisation using complex **3** in THF at T = 25 °C and $[H_3B{\cdot}NMeH_2]$ = 0.33 M.

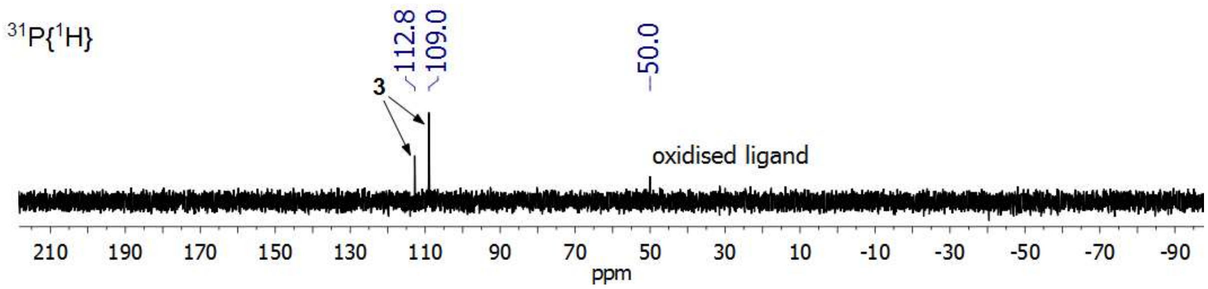

Figure S38. *In situ* ^{31}P NMR spectrum (121 MHz, toluene-d_8) recorded at the end of $H_3B{\cdot}NMe_2H$ dehydropolymerisation using complex **3** in toluene at T = 25 °C and [$H_3B{\cdot}NMeH_2$] = 0.33 M.

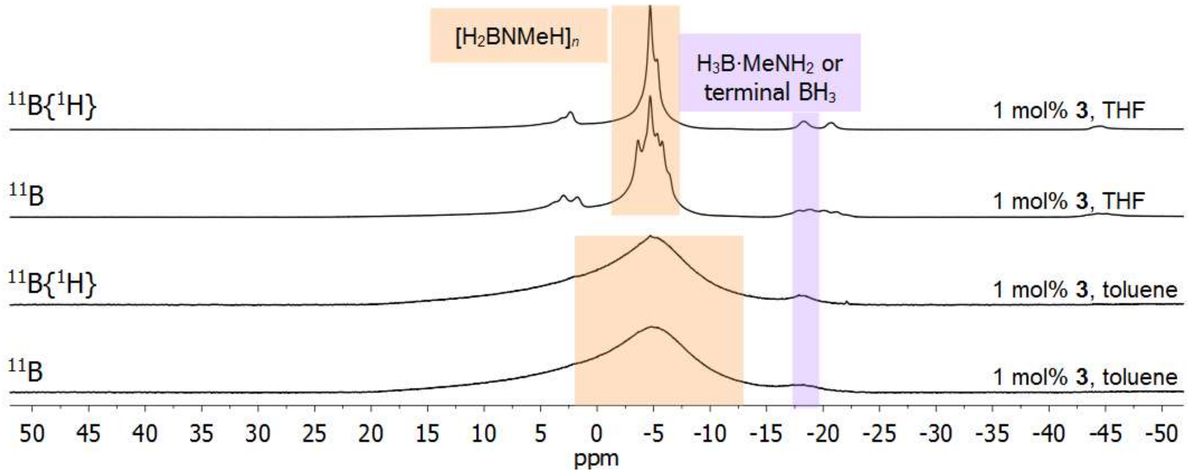

Figure S39. ^{11}B and ^{11}B{^{1}H} NMR spectra (96 MHz $CDCl_3$) of isolated [H_2BNMeH]$_n$ from dehydropolymerisation using complex **3** in THF and toluene. The signals at 2 and -21 ppm might indicate some cross linking motifs.

Polymer analysis

SEC data

Table S2. SEC-LS data.

entry	reaction conditions [a]	t [h]	M_n [g·mol^{-1}] [b]	M_w [g·mol^{-1}] [b]	$Đ$
1	2 mol% **1**	5	13400	17500	1.3
2	1 mol% **1**	1	9100	12800	1.4
3	0.5 mol% **1**	1.5	8000	10900	1.4
4	0.5 mol% **1**, closed system	24	6500	15900	2.4
5	1 mol% **3**	48	1050	1790	1.7

[a] THF, T = 25 °C, [$H_3B{\cdot}NMeH_2$] = 0.33 M. [b] absolute molecular weights determined using light scattering detection.

Table S3. Comparison of molecular weights of selected polymer samples.

		LS detector (absolute molecular weight)		*Calibration with polystyrene standard (relative molecular weight)*	
entry	reaction conditions	M_n [g·mol^{-1}]	M_w [g·mol^{-1}]	M_n [g·mol^{-1}]	M_w [g·mol^{-1}]
1[a]	2 mol% **1**, THF	13400	17500	26500	81000
2[a]	1 mol% **1**, THF	9100	12800	62500	134000
3[a]	0.5 mol% **1**, THF	8000	10900	49000	111000
4[a]	2 mol% **1**, toluene	24300	85400	83000	305000
5[a]	1 mol% **1**, toluene	18900	78600	81000	293000
6[a]	0.5 mol% **1**, toluene	13000	32500	88000	209000
6[b]	1 mol% **1**, toluene	11400	14400	64400	102000
6[c]	0.5 mol% **1**, toluene	61200	98500	119000	205000

[a] T = 25 °C, [$H_3B{\cdot}NMeH_2$] = 0.33 M. [b] Co-dehydropolymerisation of $H_3B{\cdot}NMeH_2$ and **4** (1:1) [amine borane] = 0.33 M, T = 25 °C. [c] T = 25 °C, [**4**] = 0.33 M, addition of 10 mol% NEt_2Me.

Polymer growth kinetics

$H_3B{\cdot}NMeH_2$ (60 mg, 1.33 mmol) and the corresponding Fe catalyst were weighed in the glovebox and transferred to a three-necked reaction vessel. Then, $H_3B{\cdot}NMeH_2$ containing dehydrogenation vessel was connected to the gas buret under Ar atmosphere. The gas buret was initialised and $H_3B{\cdot}NMeH_2$ and Fe catalyst were dissolved in 4 mL of THF or toluene and data acquisition was started immediately. The reaction was stopped at certain points and the solution was immediately transferred into a Schlenk flask containing 50 ml cold *n*-hexane (-78°C). The polymer was precipitated, filtered off and dried in vacuum overnight.

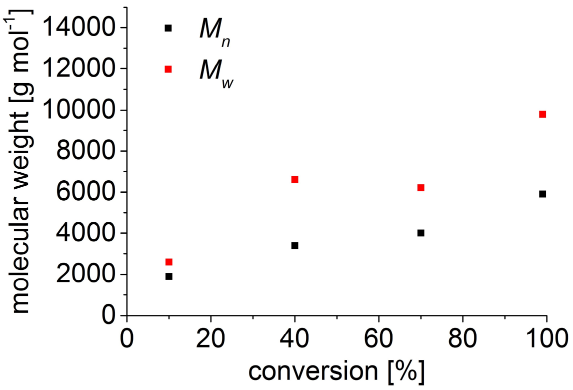

Figure S40. Polyaminoborane growth kinetics in THF. Reaction conditions: 0.5 mol% 1, $[H_3B{\cdot}NMeH_2]$ = 0.33 M, T = 25 °C, isobaric system connected to gas buret. Conversion was determined from volumetric data.

In situ NMR spectroscopy

In situ dehydropolymerisation of $H_3B{\cdot}NMeH_2$ using complex 1 in the presence of cyclohexene (NMR experiment)

In a Young NMR tube, $H_3B{\cdot}NMeH_2$ (7.3 mg, 0.163 mmol) and **1** (1 mol%, 0.64 mg, 0.00163 mmol) were dissolved in 0.4 mL of THF-*d*$_8$ or toluene-*d*$_8$ and cyclohexene (80.4 mg, 0.1 mL, 0.978 mmol, 6 equivalents) was added. To record the NMR spectra, the NMR tube was vented at regular intervals.

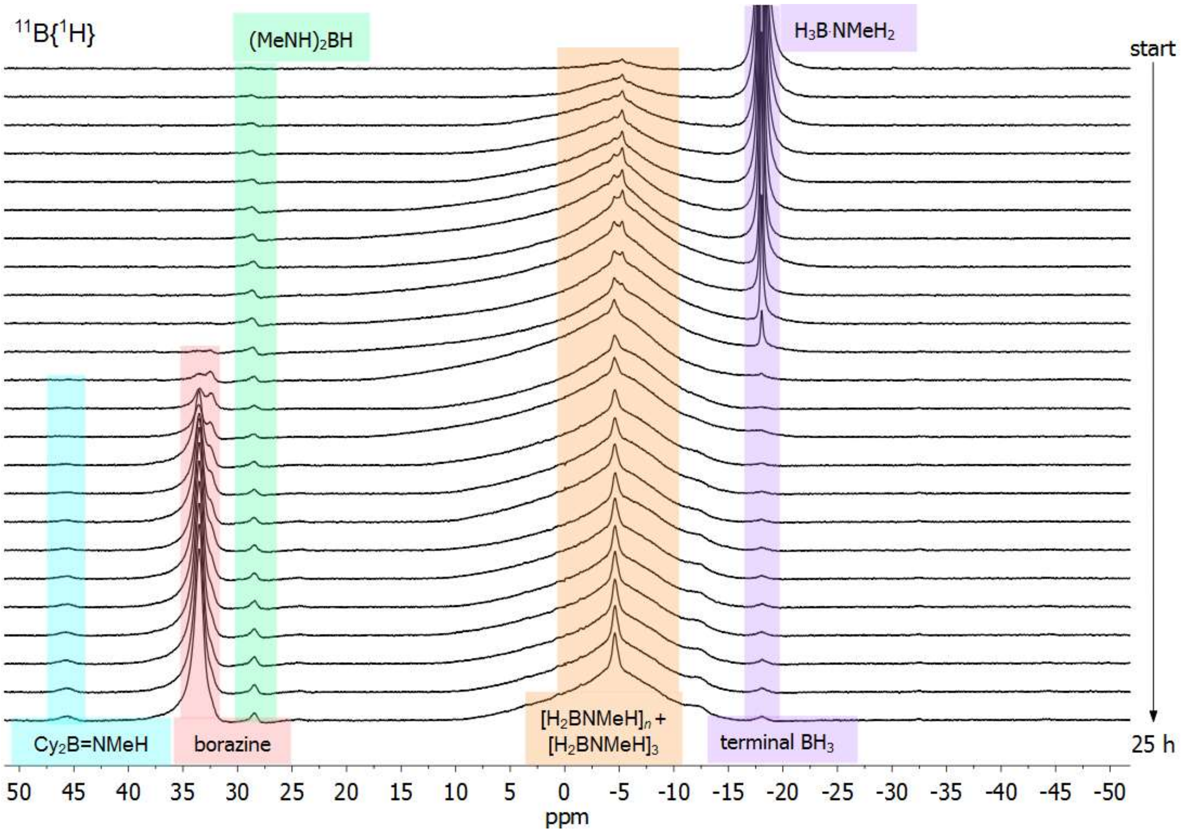

Figure S41. *In situ* $^{11}B\{^1H\}$ NMR spectra (96 MHz, THF-*d*$_8$, 1024 scans) recorded during dehydropolymerisation of $H_3B{\cdot}NMeH_2$ using complex **1** in presence of cyclohexene.

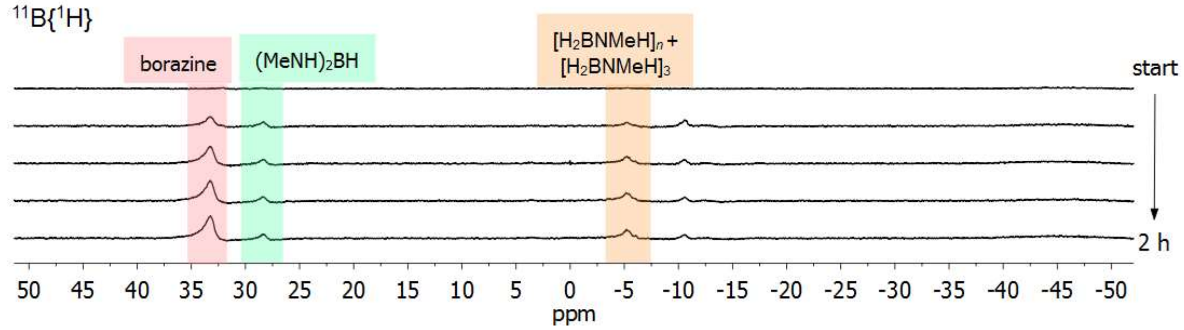

Figure S42. *In situ* $^{11}B\{^1H\}$ NMR spectra (96 MHz, toluene-*d*$_8$, 1024 scans) recorded during dehydropolymerisation of $H_3B{\cdot}NMeH_2$ using complex **1** in presence of cyclohexene. The reaction in toluene was too fast to monitor the first dehydrogenation step. Compared to reactions in THF no $Cy_2B{=}NMeH$ was observed.

In situ depolymerisation of $[H_2B{\cdot}NMeH]_n$ using complex 1 (NMR experiment)

In a Young NMR tube the polymer $[H_2B{\cdot}NMeH]_n$ (7 mg, 0.163 mmol) and **1** (1 mol%, 0.64 mg, 0.00163 mmol) were dissolved in 0.5 mL of THF-d_8 or toluene-d_8. The NMR tube was vented at regular intervals.

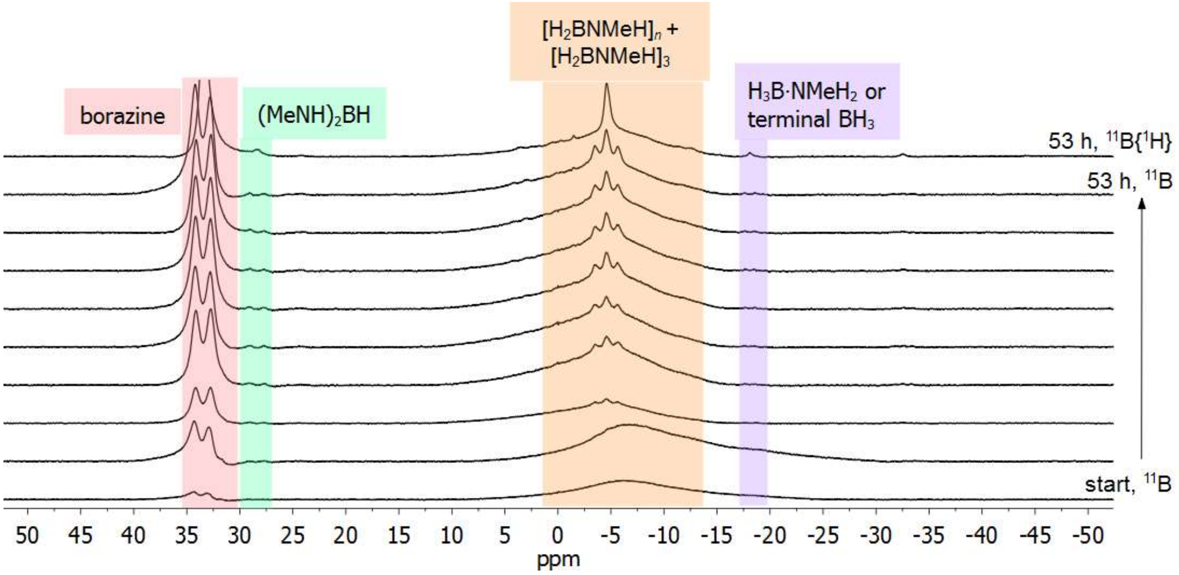

Figure S43. *In situ* ^{11}B and $^{11}B\{^1H\}$ NMR spectra (96 MHz, THF-d_8, 512 scans) recorded during depolymerisation of $[H_2B{\cdot}NMeH]_n$ using complex **1**.

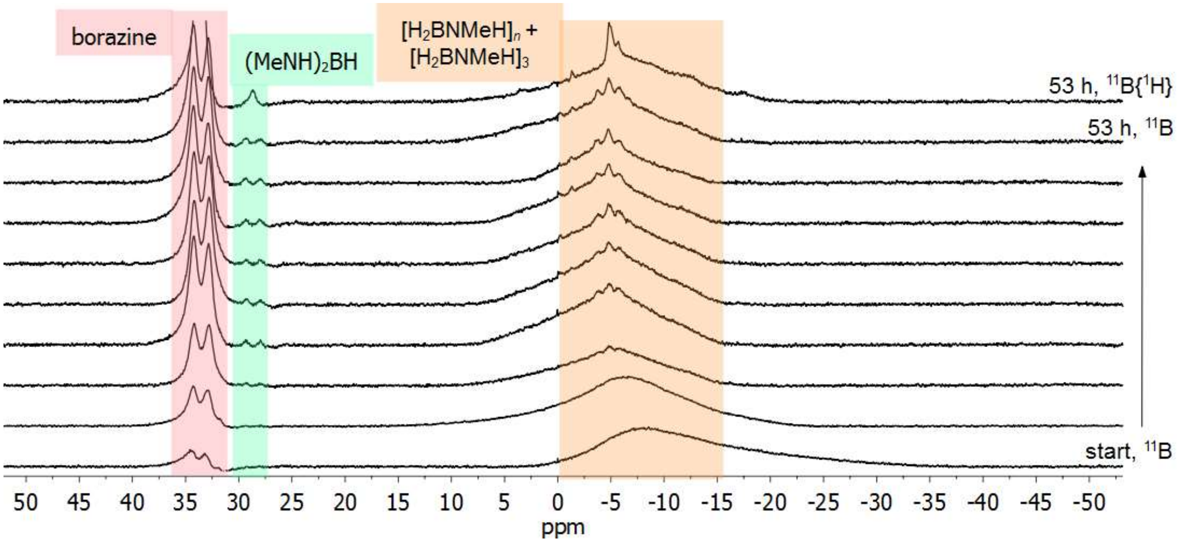

Figure S44. *In situ* ^{11}B and $^{11}B\{^1H\}$ NMR spectra (96 MHz, toluene-d_8, 512 scans) recorded during depolymerisation of $[H_2B{\cdot}NMeH]_n$ using complex **1**.

The experiment was repeated in 0.4 mL solvent and cyclohexene (80.4 mg, 0.1 mL, 0.978 mmol, 6 eq.) was added to the solution.

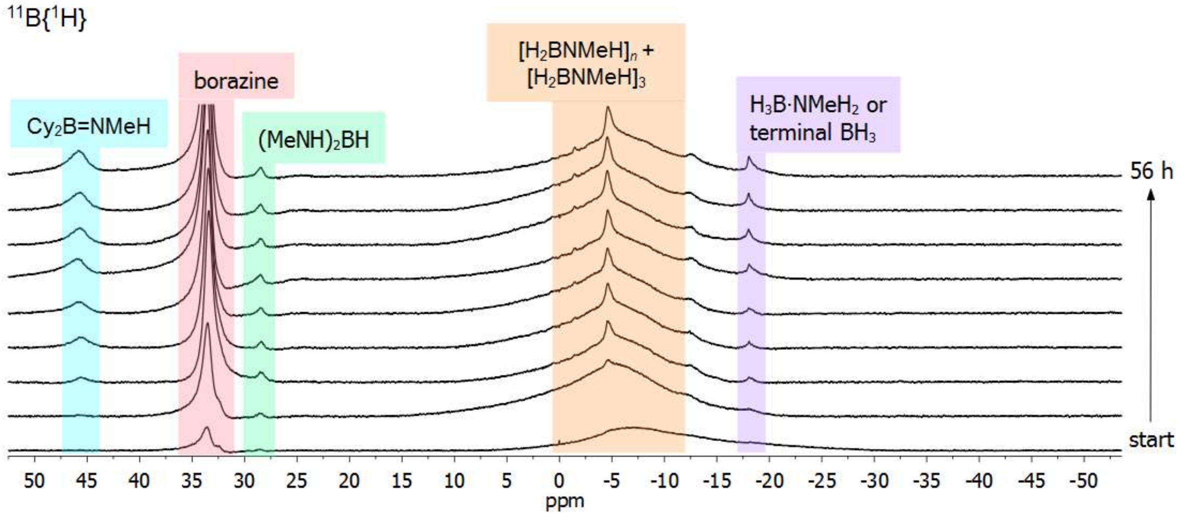

Figure S45. *In situ* ^{11}B and $^{11}B\{^1H\}$ NMR spectra (96 MHz, THF-d_8, 512 scans) recorded during depolymerisation of $[H_2B{\cdot}NMeH]_n$ using complex **1** in the presence of cyclohexene.

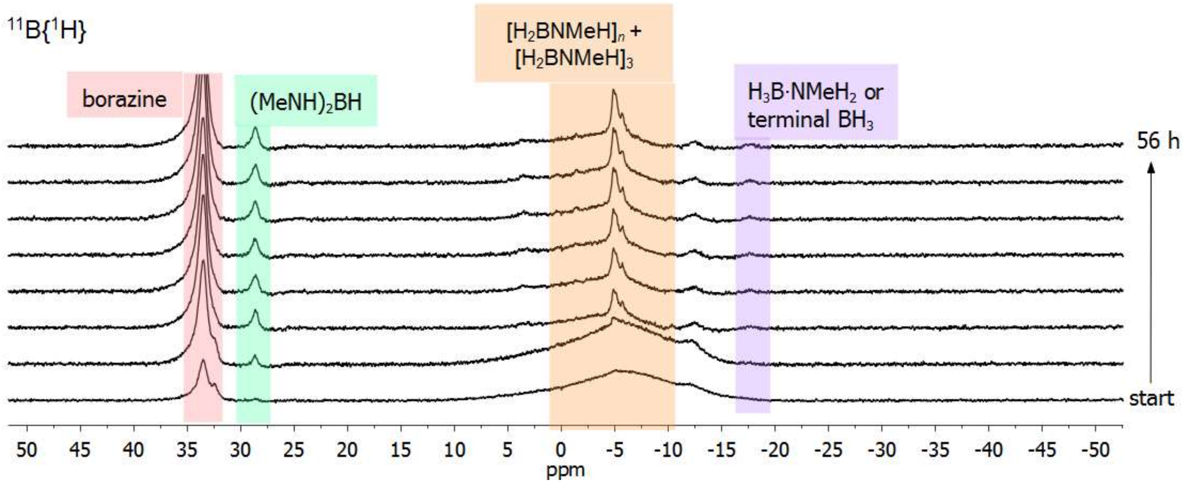

Figure S46. *In situ* ^{11}B and $^{11}B\{^1H\}$ NMR spectra (96 MHz, toluene-d_8, 512 scans) recorded during depolymerisation of $[H_2B{\cdot}NMeH]_n$ using complex **1** in the presence of cyclohexene.

In situ dehydrogenation of cyclotriborazane $(H_2B{\cdot}NMeH)_3$ using complex 1 (NMR experiment)

In a Young NMR tube $(H_2B{\cdot}NMeH)_3$ (21 mg, 0.163 mmol) and **1** (1 mol%, 0.64 mg, 0.00163 mmol) were dissolved in 0.5 mL of THF-d_8 or toluene-d_8. The NMR tube was vented at regular intervals.

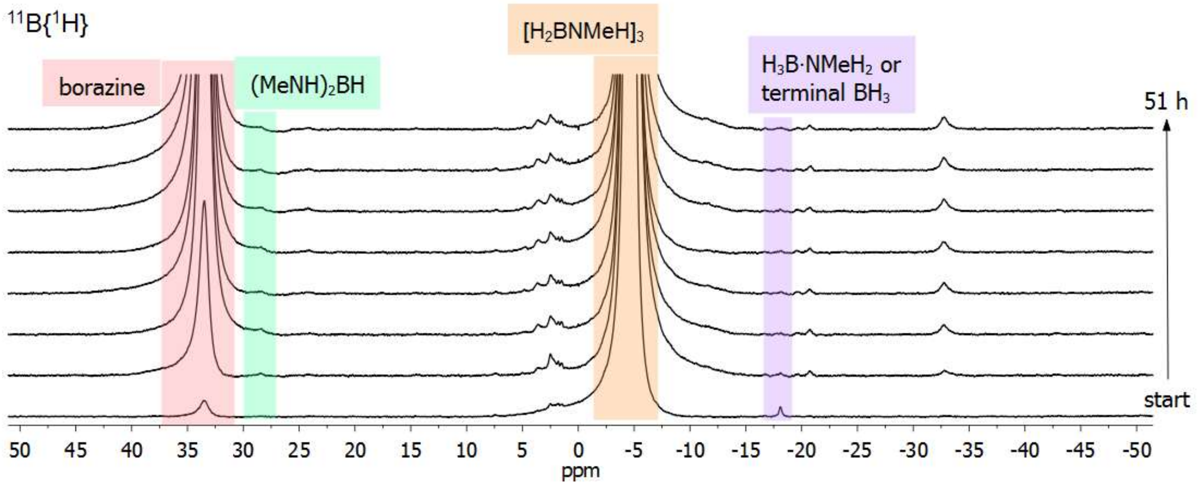

Figure S47. *In situ* $^{11}B\{^1H\}$ NMR spectra (96 MHz, THF-d_8, 512 scans) recorded during dehydrogenation of $(H_2B{\cdot}NMeH)_3$ using complex **1**. The signal at -33 ppm corresponds to the Fe borate complex **2**.

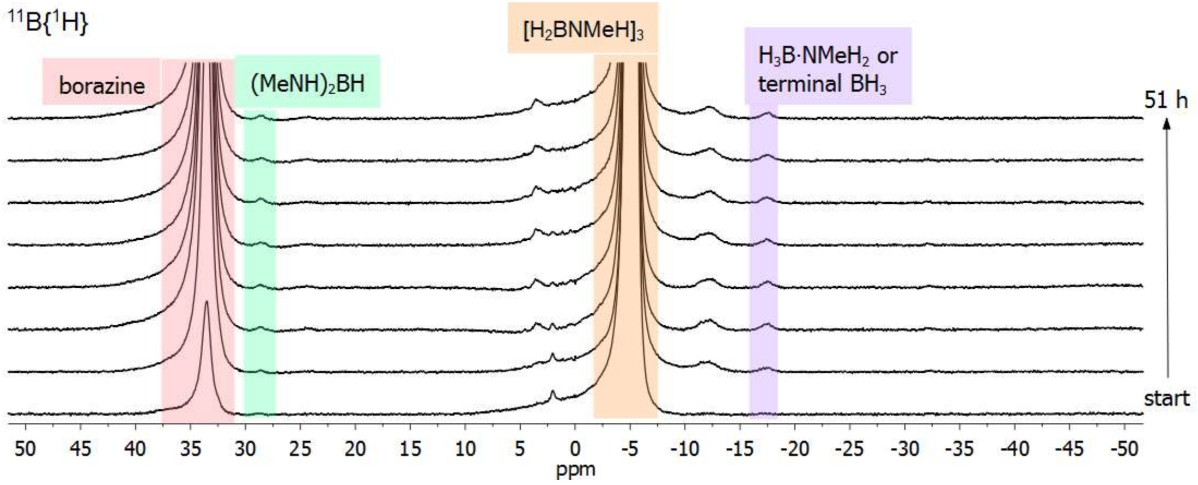

Figure S48. *In situ* $^{11}B\{^1H\}$ NMR spectra (96 MHz, toluene-d_8, 512 scans) recorded during dehydrogenation of $(H_2B{\cdot}NMeH)_3$ using complex **1**.

The experiment was repeated in 0.4 mL solvent and cyclohexene (80.4 mg, 0.1 mL, 0.978 mmol, 6 eq.) was added to the solution.

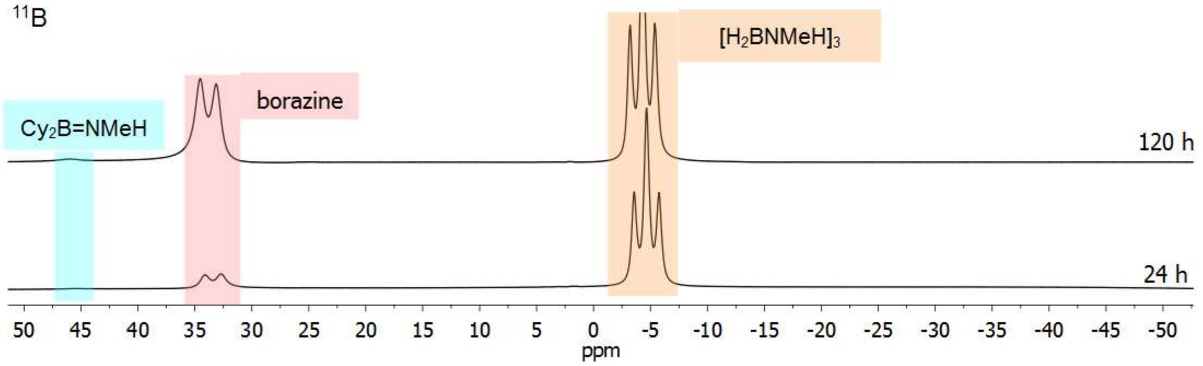

Figure S49. *In situ* ^{11}B NMR spectra (96 MHz, THF-d_8, 512 scans) recorded during dehydrogenation of $(H_2B{\cdot}NMeH)_3$ using complex **1** in the presence of cyclohexene.

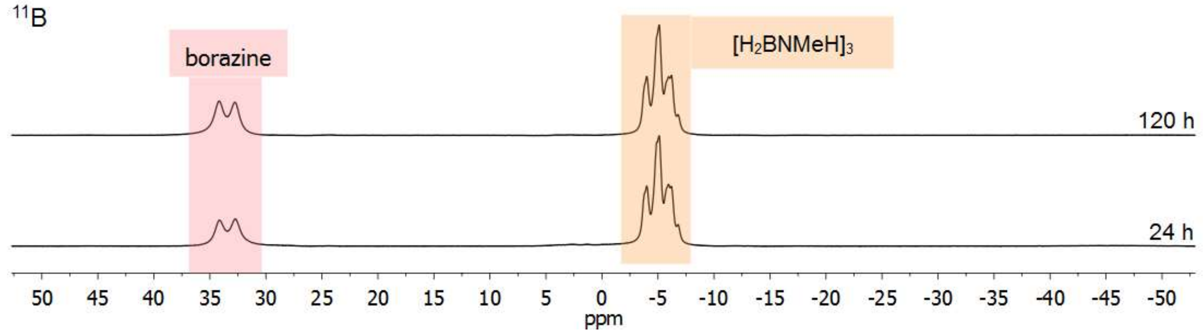

Figure S50. *In situ* ^{11}B NMR (96 MHz, toluene-d_8, 512 scans) spectra recorded during dehydrogenation of $(H_2B{\cdot}NMeH)_3$ using complex **1** in the presence of cyclohexene.

In situ **dehydropolymerisation of $H_3B{\cdot}NMeH_2$ using complex 3 in the presence of cyclohexene (NMR experiment)**

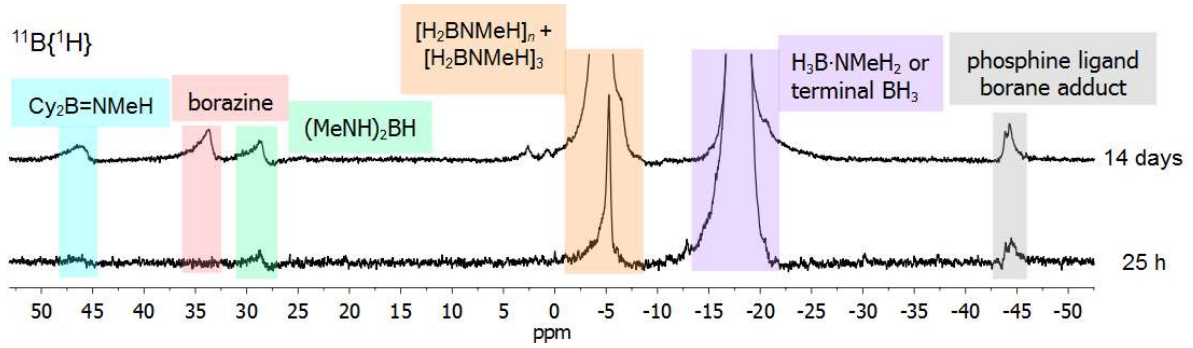

Figure S51. *In situ* $^{11}B\{^1H\}$ NMR spectra (96 MHz, THF-d_8, 1024 scans) recorded during dehydropolymerisation of $H_3B{\cdot}NMeH_2$ using complex **3** in presence of cyclohexene in THF at T = 25 °C and $[H_3B{\cdot}NMeH_2]$ = 0.33 M.

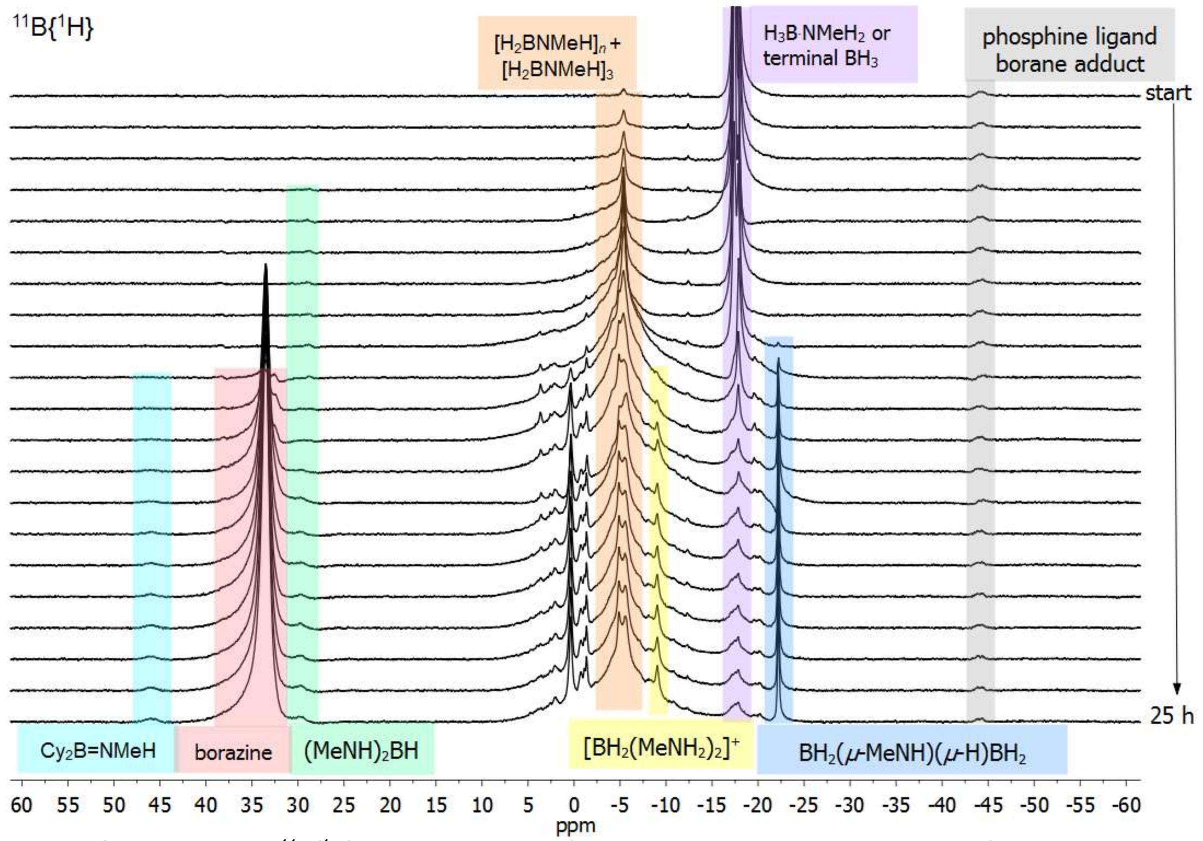

Figure S52. *In situ* $^{11}B\{^1H\}$ NMR spectra (96 MHz, toluene-d_8, 1024 scans) recorded during dehydropolymerisation of $H_3B{\cdot}NMeH_2$ using complex **3** in presence of cyclohexene in toluene at T = 25 °C and [$H_3B{\cdot}NMeH_2$] = 0.33 M.

Stoichiometric reaction of 1 with $H_3B{\cdot}NMeH_2$

In a Young NMR tube $H_3B{\cdot}NMeH_2$ (1.3 mg, 0.028 mmol) and **1** (10 mg, 0.026 mmol) were dissolved in 0.6 mL of THF-d_8 or toluene-d_8. The solution changes its colour immediately from violet to yellow and was analysed by NMR spectroscopy. A new aminoborane capped Fe species [(PN^{BH_2NMeH}P)Fe(H)CO] **1-H₂BNMeH** was detected as major product along with a minor amount of the dihydrido complex [(PN*H*P)Fe(H_2)CO], similar to the reaction of the related Ru complex [(PNP)Ru(H)PMe_3 with amine borane.[21] Complex **1-H₂BNMeH** consists also of two isomers (*cis/trans*).

^{1}H NMR (300 MHz, THF-d_8, 22°C): δ -14.27 (dt, $^2J_{PH}$ = 58.9 Hz, $^2J_{H\text{-}H}$ = 6.2 Hz, 1H, Fe−H), -10.73 (br, 1H, Fe−HB). ^{11}B NMR (96 MHz, THF-d_8, 22°C): δ -10.2 (d br, $^2J_{BH}$ = 111 Hz). $^{31}P\{^1H\}$ NMR (121 MHz, THF-d_8, 22 °C): δ 110.5 (s). ^{1}H NMR (300 MHz, C_6D_6, 22°C): δ −14.23 (dt, $^2J_{PH}$ = 58.5 Hz, $^2J_{H\text{-}H}$ = 6.1 Hz, 1H, Fe−H), -10.37 (br, 1H, Fe−HB). ^{11}B NMR (96 MHz, C_6D_6, 22°C): δ -10.2 (d br, $^2J_{BH}$ = 111 Hz). $^{31}P\{^1H\}$ NMR (121 MHz, C_6D_6, 22 °C): δ 110.3(s).

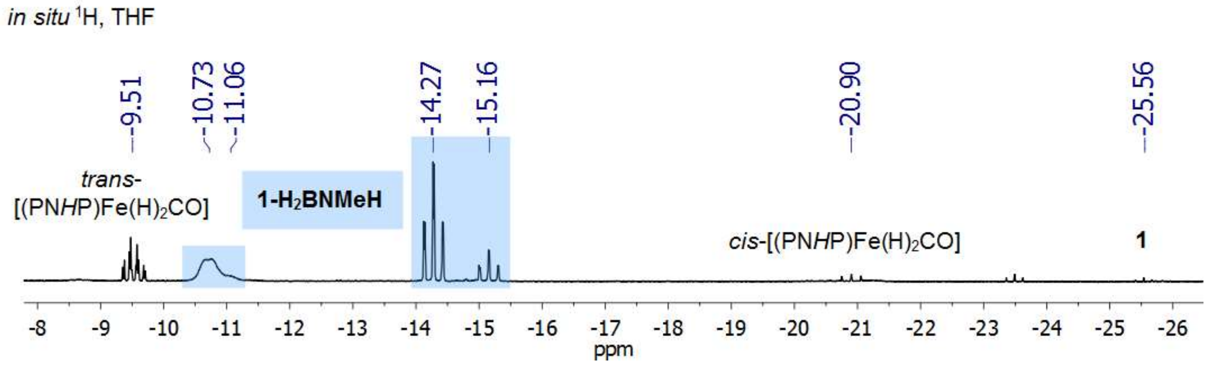

Figure S53. *In situ* ^{1}H NMR spectra (300 MHz, THF-d_8) from the stoichiometric reaction of **1** with $H_3B{\cdot}NMeH_2$.

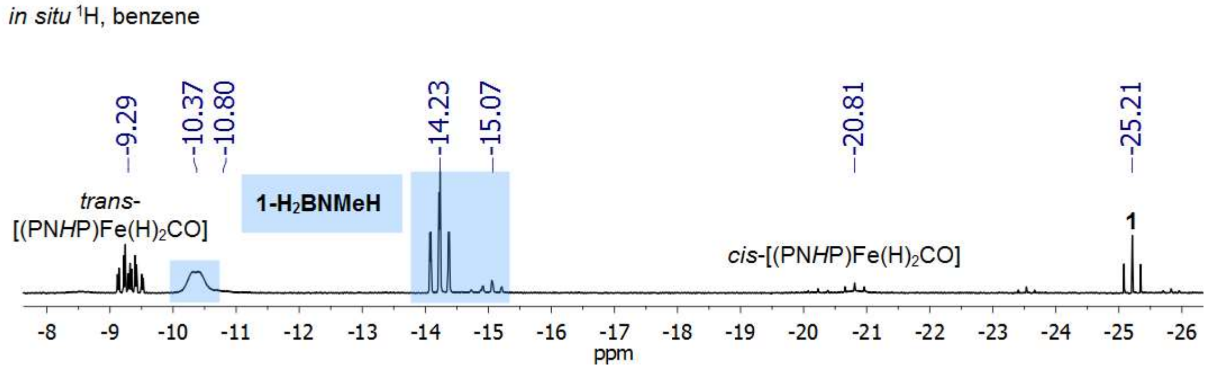

Figure S54. *In situ* ^{1}H NMR spectra (300 MHz, benzene-d_6) of a stoichiometric reaction of **1** with $H_3B{\cdot}NMeH_2$.

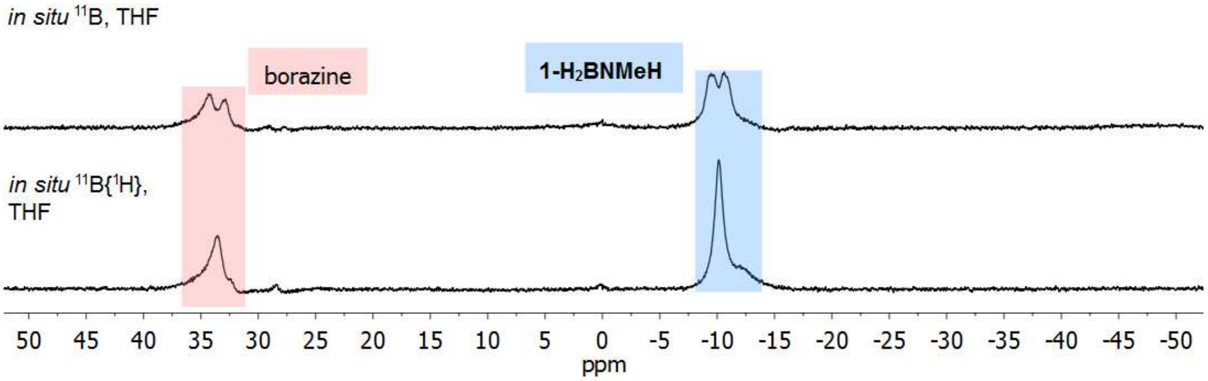

Figure S55. *In situ* ^{11}B, $^{11}B\{^{1}H\}$ NMR spectra (96 MHz, THF-d_8) of a stoichiometric reaction of **1** with $H_3B{\cdot}NMeH_2$.

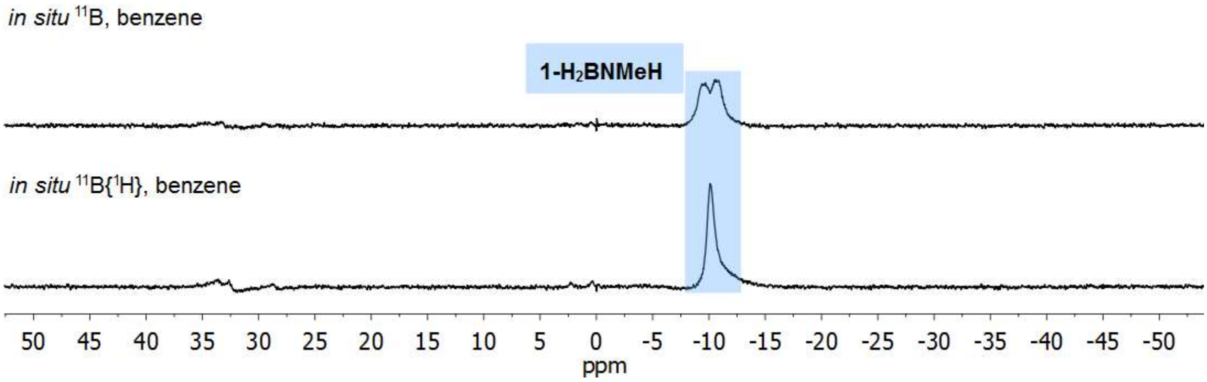

Figure S56. *In situ* ^{11}B, $^{11}B\{^1H\}$ NMR spectra (96 MHz, benzene-d_6) of a stoichiometric reaction of **1** with $H_3B{\cdot}NMeH_2$.

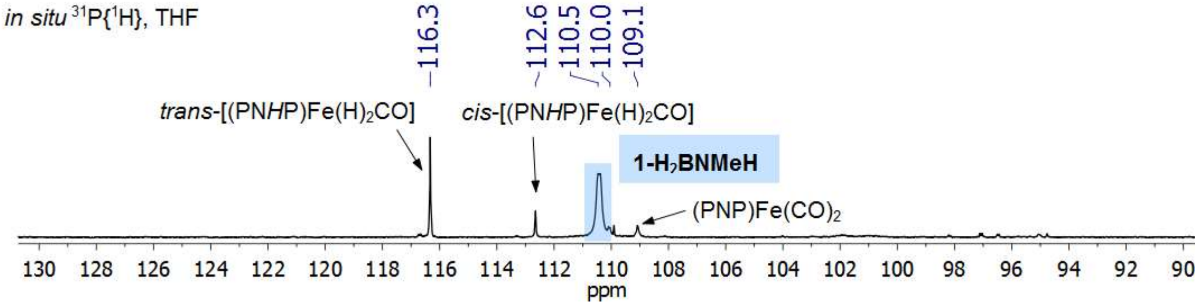

Figure S57. *In situ* $^{31}P\{^1H\}$ NMR spectra (121 MHz, THF-d_8) of a stoichiometric reaction of **1** with $H_3B{\cdot}NMeH_2$. $(PNP)Fe(CO)_2$[22].

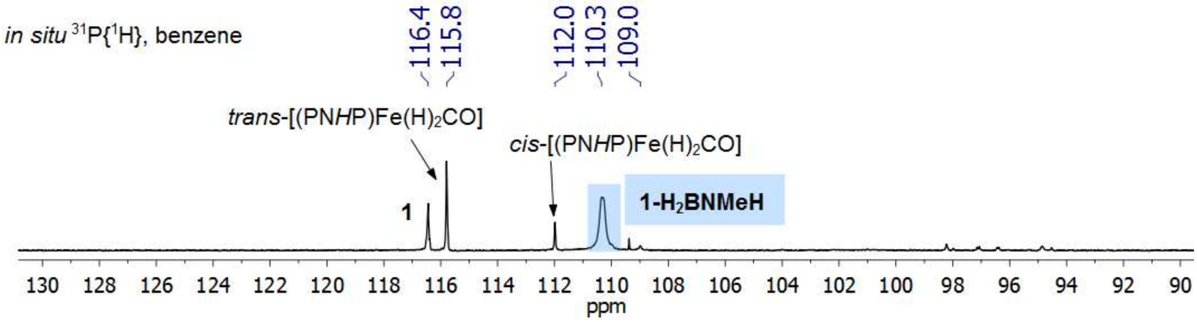

Figure S58. *In situ* $^{31}P\{^1H\}$ NMR spectra (121 MHz, benzene-d_6) of a stoichiometric reaction of **1** with $H_3B{\cdot}NMeH_2$.

Stoichiometric reaction of 3 with $H_3B{\cdot}NMeH_2$

In a Young NMR tube $H_3B{\cdot}NMeH_2$ (1.3 mg, 0.028 mmol) and **3** (11 mg, 0.026 mmol) were dissolved in 0.6 mL of THF-d_8 or toluene-d_8. No other Fe species then **3** was detected by $^{31}P\{^1H\}$ NMR spectroscopy.

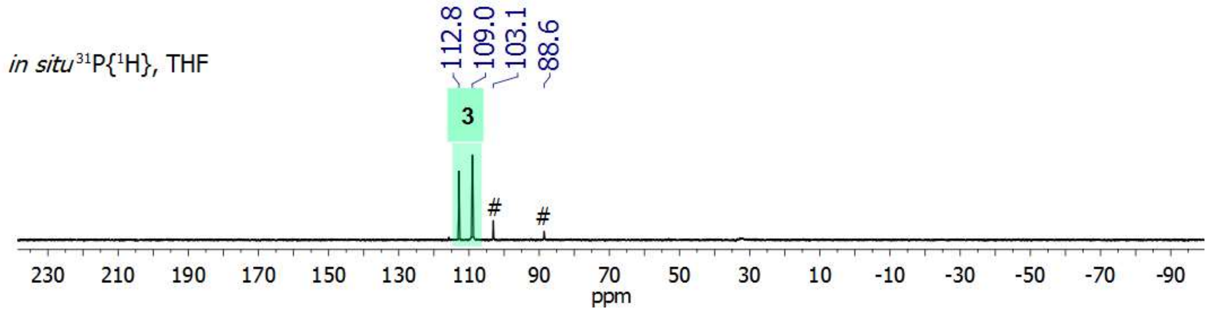

Figure S 59. *In situ* $^{31}P\{^1H\}$ NMR spectra (121 MHz, THF-d_8) of a stoichiometric reaction of **3** with $H_3B{\cdot}NMeH_2$. # Impurities from the synthesis of complex **3**.

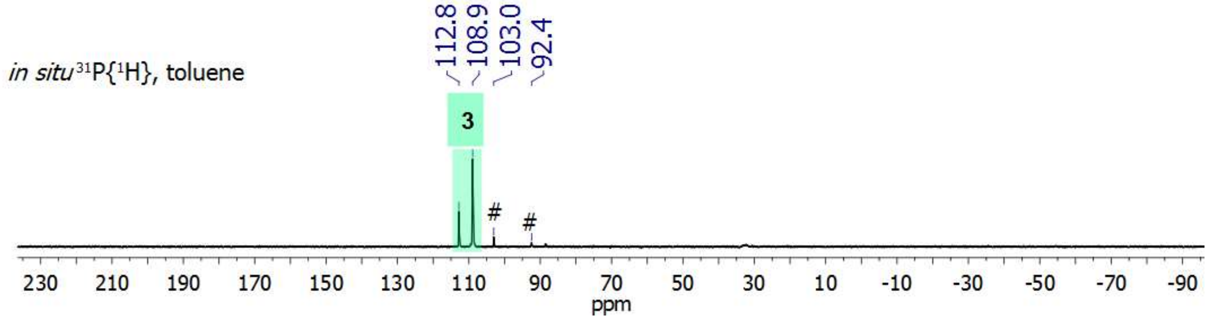

Figure S 60. *In situ* $^{31}P\{^1H\}$ NMR spectra (121 MHz, toluene-d_8) of a stoichiometric reaction of **3** with $H_3B{\cdot}NMeH_2$. # Impurities from the synthesis of complex **3**.[5]

Catalytic dehydrocoupling of $H_3B{\cdot}NMe_2H$

$H_3B{\cdot}NMe_2H$ (78 mg, 1.33 mmol) and the corresponding Fe catalyst were weighed in the glovebox and transferred to a three-necked reaction vessel. Then, the $H_3B{\cdot}NMe_2H$ containing dehydrogenation vessel was connected to the gas buret under Ar atmosphere. The gas buret was initialised and $H_3B{\cdot}NMe_2H$ and Fe catalyst were dissolved in 4 mL of THF or toluene and data acquisition was started immediately. After completion of the dehydrogenation reaction, a gas sample was taken and analysed by GC-TCD and an aliquot was analysed by ^{11}B NMR spectroscopy.

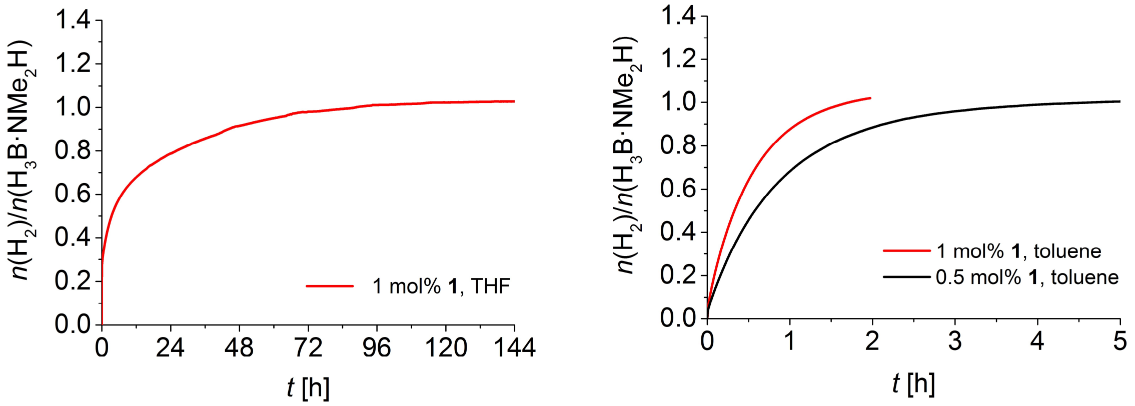

Figure S61. Volumetric curves of $H_3B{\cdot}NMe_2H$ dehydrocoupling using complex **1** in THF (left) and toluene (right) at T = 25 °C and $[H_3B{\cdot}NMe_2H]$ = 0.33 M.

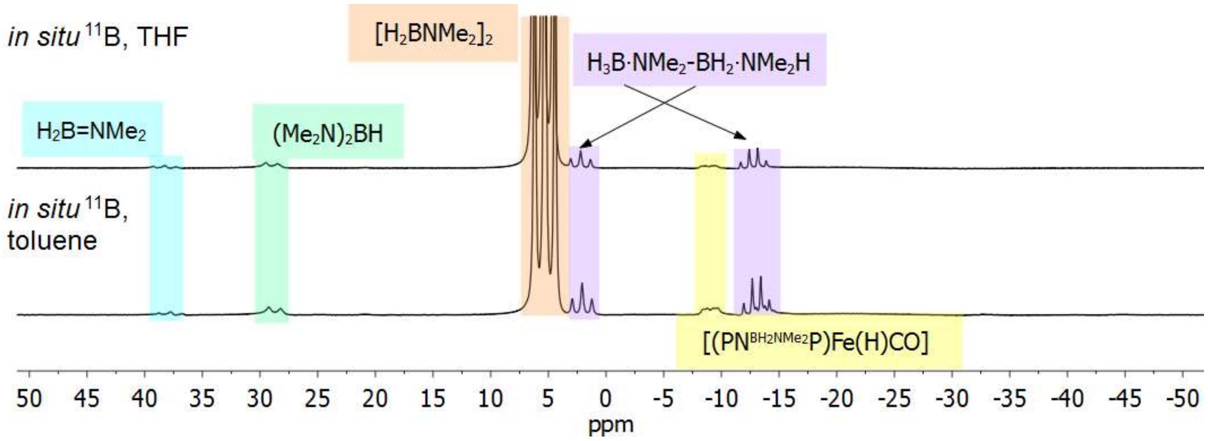

Figure S62. *In situ* ^{11}B NMR (96 MHz, 512 scans) spectra at the end of $H_3B{\cdot}NMe_2H$ dehydrocoupling using complex **1** in THF (top) and toluene (bottom) at T = 25 °C and $[H_3B{\cdot}NMe_2H]$ = 0.33 M.

Catalytic dehydrocoupling of $H_3B{\cdot}N(CH_2SiMe_3)H_2$

Table S4. ^{11}B NMR chemical shifts of amine borane species.

compound	δ ^{11}B	
	R = Me	R = CH_2SiMe_3
aminoborane<br>$H_2B{=}NRH$	37.1 ppm (-10 °C)<br>(Et_2O, t, J_{BH} = 130 Hz)[23]	37.5 ppm<br>(toluene-d_8, br)
borazine<br>$[HBNR]_3$	32.3 ppm<br>(THF, d, J_{BH} = 135 Hz)[2]	32.0 ppm<br>(toluene-d_8, br)
diaminoborane<br>$HB(NRH)_2$	27.7 ppm<br>(THF, d br)[2]	28.5 ppm<br>(THF-d_8, d br, J_{BH} = 107 Hz)
cyclic triborazane<br>$[H_2B{-}NRH]_3$	-5.4 ppm, two isomers<br>(acetone-d_6, t, J_{BH} = 107 Hz)[24]	-4.5 ppm<br>(THF-d_8, t br, J_{BH} = 106 Hz)
amine borane<br>$H_3B{\cdot}NRH_2$	-18.8 ppm<br>($CDCl_3$, q, J_{BH} = 94 Hz)[1]	-17.5 ppm<br>($CDCl_3$, q, J_{BH} = 95 Hz)
aminodiborane<br>$(H_2B)_2(\mu\text{-}NRH)(\mu\text{-}H)$	-22.7 ppm<br>(Et_2O, td, J_{BH} = 130 Hz, J_{BH} = 30 Hz)[25]	-22 ppm<br>(THF-d_8, t br, J_{BH} = 135 Hz)

Catalytic dehydropolymerisation of 4 with [(POCOP)IrH$_2$] and [Rh(COD)Cl]$_2$ in THF: Amine borane **4** (156 mg, 1.33 mmol) and the corresponding catalyst were weighed in the glovebox and transferred to a three-necked reaction vessel. Then, the dehydrogenation vessel containing **4** was connected to the gas buret under Ar atmosphere. The gas buret was initialised and **4** and the catalyst were dissolved in 4 mL of THF and data acquisition was started immediately. After completion of the dehydrogenation reaction, a gas sample was taken and analysed by GC-TCD and an aliquot was analysed by ^{11}B NMR spectroscopy.

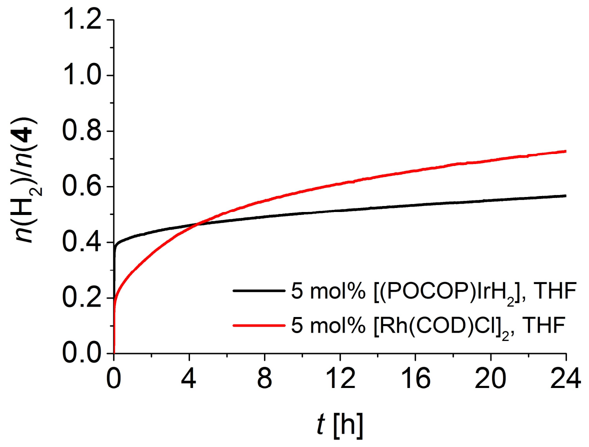

Figure S63. Volumetric curves of dehydropolymerisation of **4** using [(POCOP)IrH$_2$] and [Rh(COD)Cl]$_2$ in THF at T = 25 °C and $[H_3B{\cdot}NMeH_2]$ = 0.33 M.

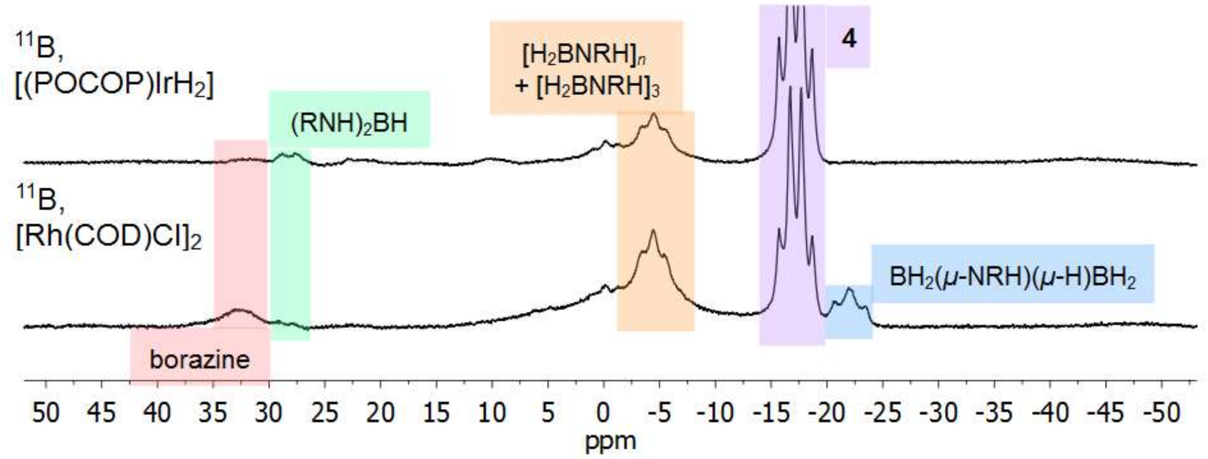

Figure S64. *In situ* ^{11}B NMR spectra (96 MHz, THF-d_8) at the end of dehydropolymerisation of **4** using [(POCOP)IrH$_2$] and [Rh(COD)Cl]$_2$ in THF at T = 25 °C and [$H_3B{\cdot}NMeH_2$] = 0.33 M. R = CH_2SiMe_3.

Catalytic dehydropolymerisation of 4 using complex 1: 4 (100 mg, 0.85 mmol) and the corresponding Fe catalyst were weighed in the glovebox and transferred to a three-necked reaction vessel. Then, the dehydrogenation vessel containing **4** was connected to the gas buret under Ar atmosphere. The gas buret was initialised and **4** and Fe catalyst were dissolved in 4 mL of THF or toluene and data acquisition was started immediately. After completion of the dehydrogenation reaction, a gas sample was taken and analysed by GC-TCD and an aliquot was analysed by ^{11}B NMR spectroscopy.

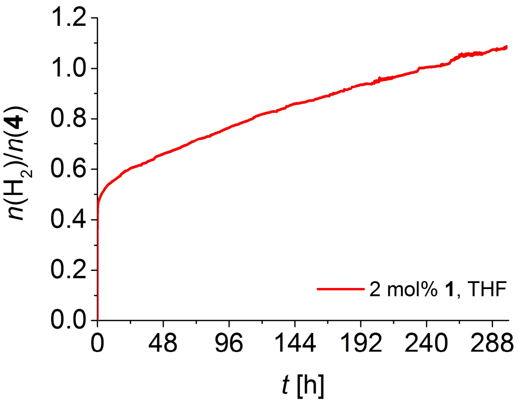

Figure S65. Volumetric curves of **4** dehydropolymerisation using complex **1** in THF and at T = 25 °C and [$H_3B{\cdot}NMeH_2$] = 0.21 M.

Reactions in THF: An increase in viscosity was not observed and the addition of *n*-hexane did not result in any precipitation. The solvent was removed in vacuum and the crude residue was analysed by NMR spectroscopy.

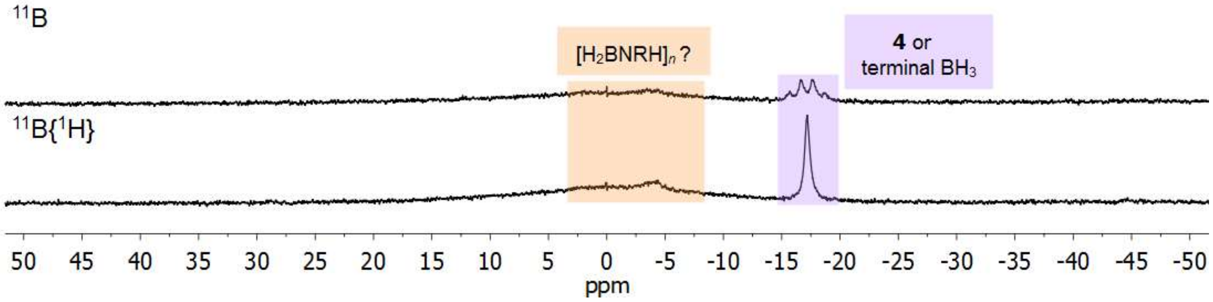

Figure S66. *In situ* ^{11}B NMR spectra (96 MHz, THF-*d*$_8$) at the end of dehydropolymerisation of **4** using complex **1** in THF at *T* = 25 °C and [$H_3B{\cdot}NRH_2$] = 0.21 M. R = CH_2SiMe_3.

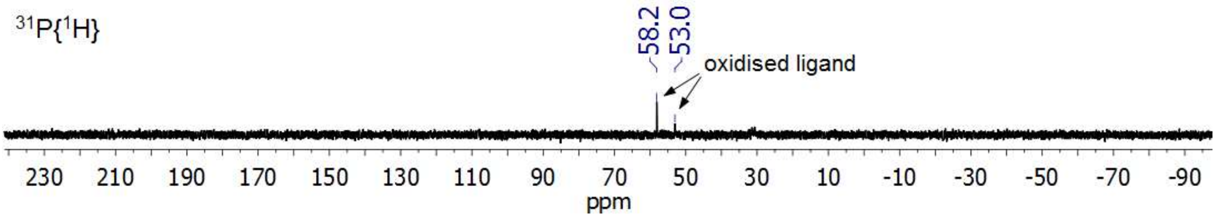

Figure S67. *In situ* ^{31}P{^{1}H} NMR spectrum (121 MHz, THF-*d*$_8$) at the end of dehydropolymerisation of **4** using complex **1** in THF at *T* = 25 °C and [$H_3B{\cdot}NRH_2$] = 0.21 M. R = CH_2SiMe_3. Oxidised PNP ligand.[5]

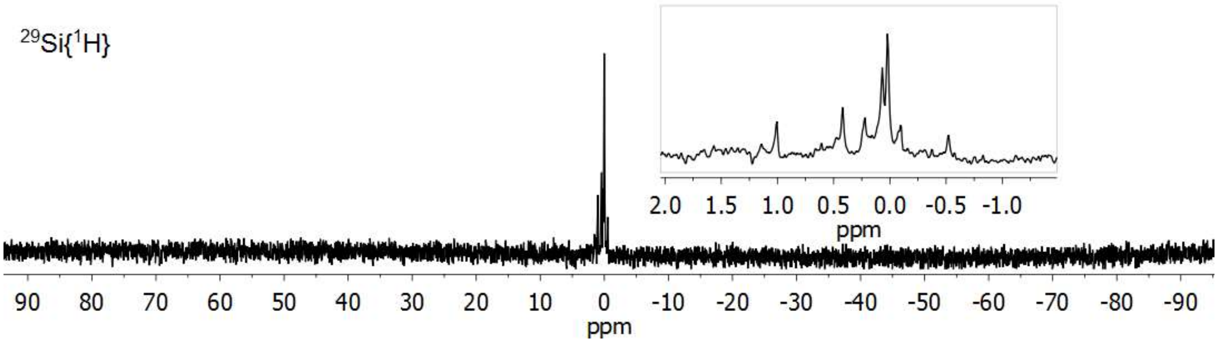

Figure S68. ^{29}Si{^{1}H} NMR spectrum (79 MHz, THF-*d*$_8$) of the solid residue from dehydropolymerisation of **4** using complex **1** in THF at *T* = 25 °C and [$H_3B{\cdot}NRH_2$] = 0.21 M. The spectra were recorded from the solid residue without further purification. R = CH_2SiMe_3.

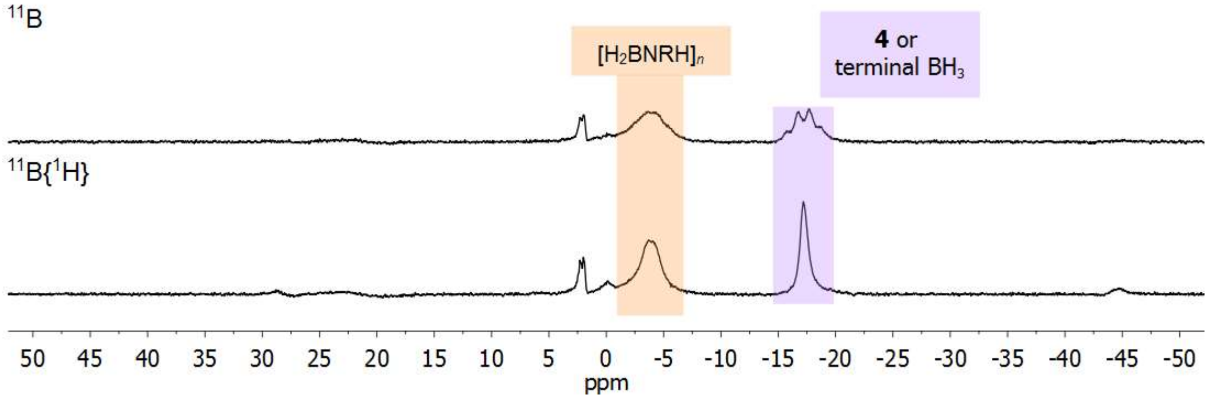

Figure S69. ^{11}B and ^{11}B{^{1}H} NMR spectra (96 MHz, THF-*d*$_8$) of the solid residue from dehydropolymerisation of **4** using complex **1** in THF at *T* = 25 °C and [$H_3B{\cdot}NRH_2$] = 0.21 M. The spectra were recorded from the solid residue without further purification. R = CH_2SiMe_3. The signal between 0 and 5 ppm might indicate some cross-linking.

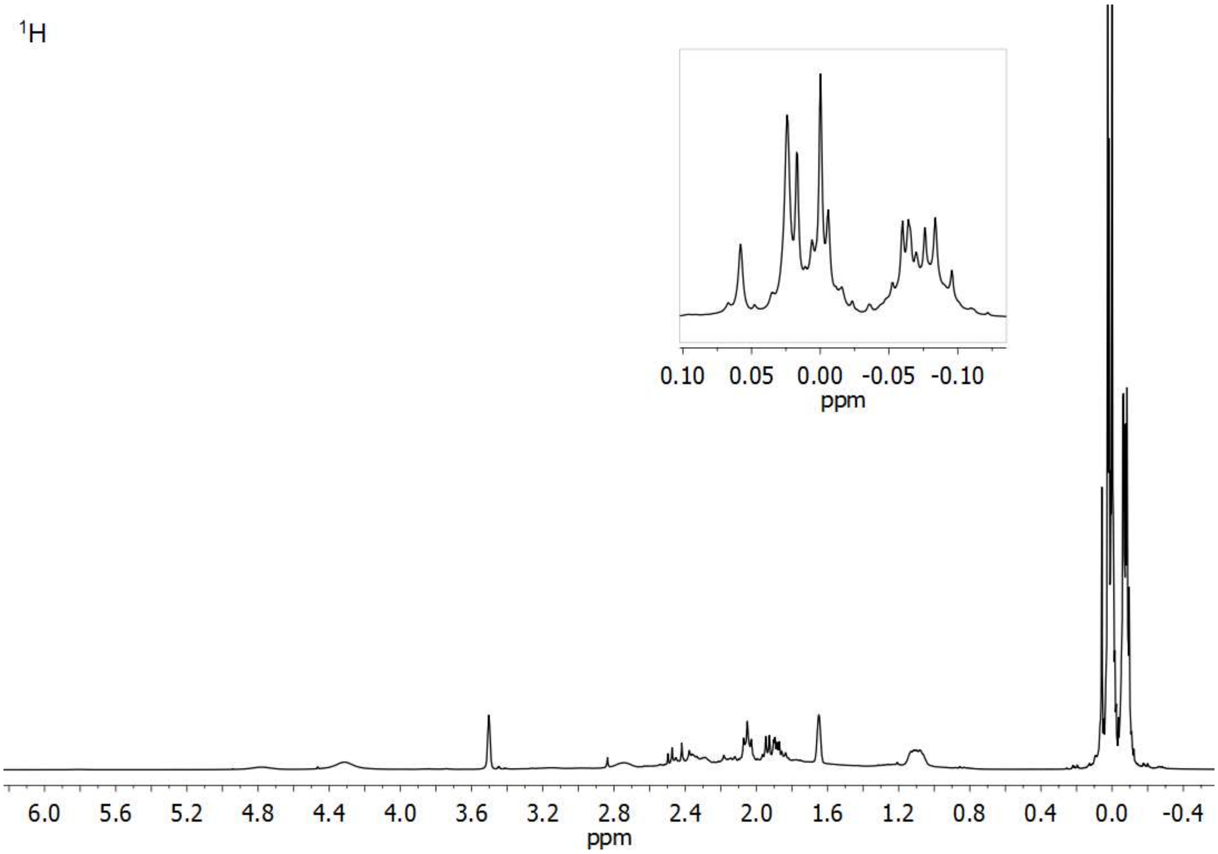

Figure S70. ^{1}H NMR spectrum (300 MHz, THF-d_8) of the solid residue from dehydropolymerisation of **4** using complex **1** in THF at T = 25 °C and [$H_3B{\cdot}NRH_2$] = 0.21 M. The spectra were recorded from the solid residue without further purification. R = CH_2SiMe_3.

Reactions in toluene: During the reaction the viscosity of the solution increased. The catalyst was separated from the polymer by addition of *n*-hexane which resulted in the precipitation of the polymer and was followed by filtration.

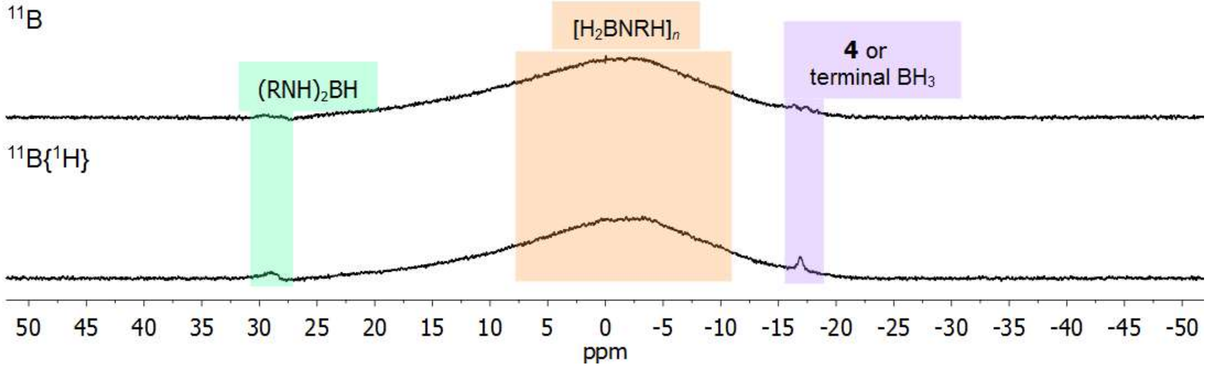

Figure S71. *In situ* ^{11}B NMR spectra (96 MHz, toluene-d_8) recorded at the end of dehydropolymerisation of **4** using 2 mol% complex **1** in toluene at T = 25 °C and [$H_3B{\cdot}NRH_2$] = 0.21 M. R = CH_2SiMe_3.

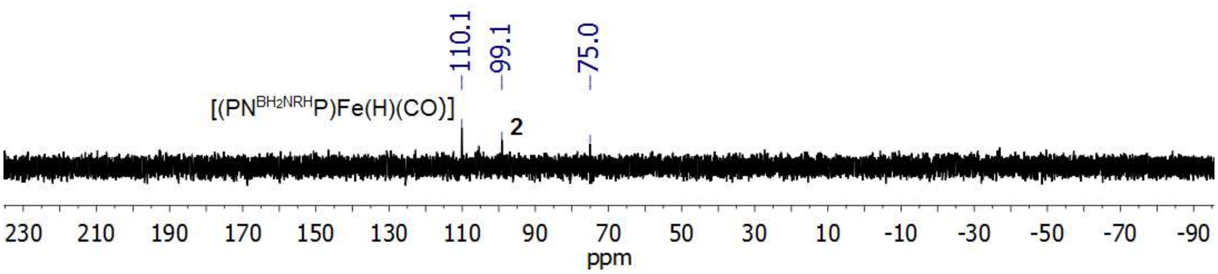

Figure S72. *In situ* $^{31}P\{^1H\}$ NMR spectrum (121 MHz, toluene-d_8) recorded at the end of dehydropolymerisation of **4** using 2 mol% complex **1** in toluene at T = 25 °C and $[H_3B{\cdot}NRH_2]$ = 0.21 M. R = CH_2SiMe_3.

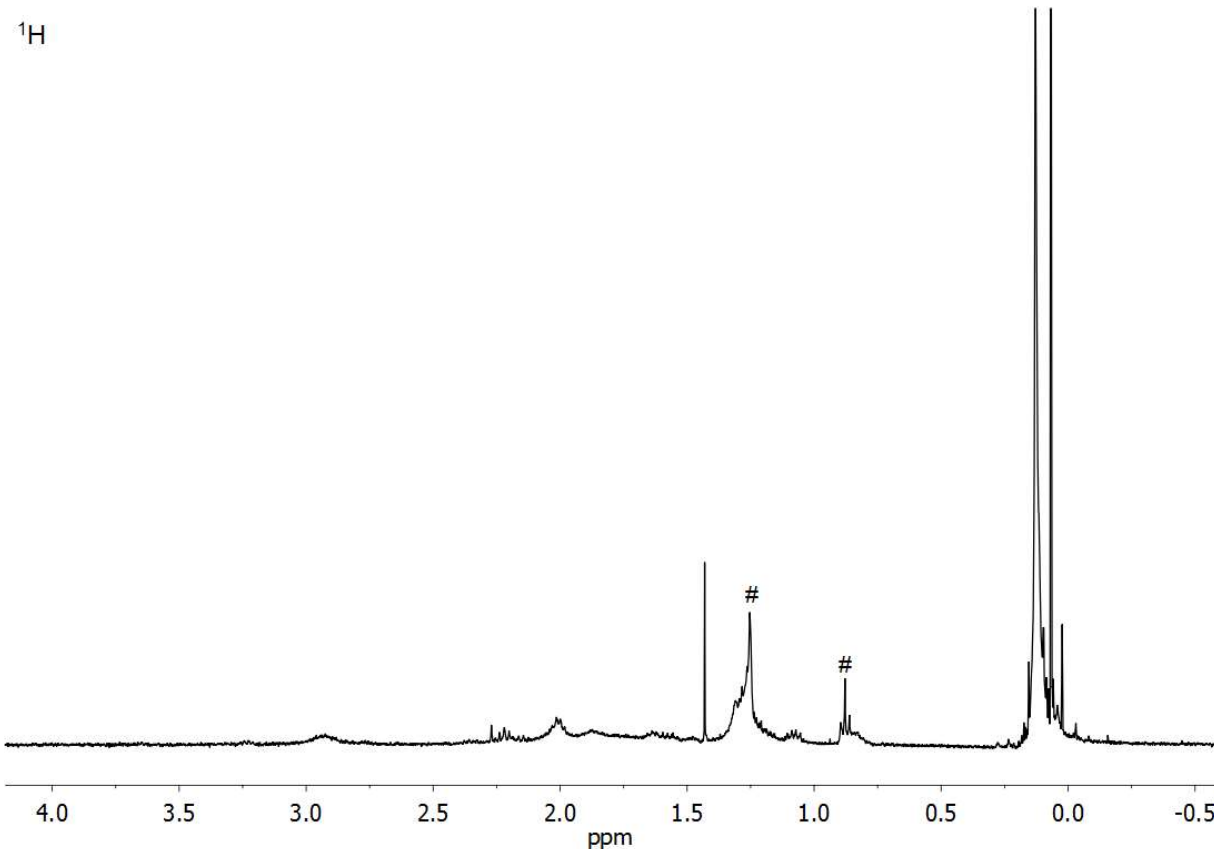

Figure S73. ^1H NMR spectrum (300 MHz, $CDCl_3$) of the solid residue from dehydropolymerisation of **4** using 2 mol% complex **1** in toluene at T = 25 °C and $[H_3B{\cdot}NRH_2]$ = 0.21 M after the separation with *n*-hexane. R = CH_2SiMe_3. # = *n*-hexane.

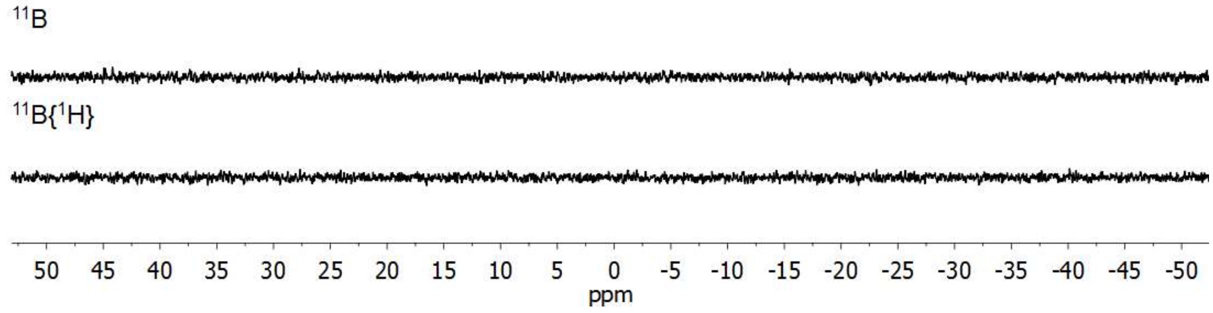

Figure S74. ^{11}B, $^{11}B\{^1H\}$ NMR spectra (96 MHz, $CDCl_3$) of the solid residue from dehydropolymerisation of **4** using complex **1** in toluene at T = 25 °C and $[H_3B{\cdot}NRH_2]$ = 0.21 M after the separation with *n*-hexane. R = CH_2SiMe_3.

Catalytic dehydropolymerisation of 4 using complex 1 in the presence of amine

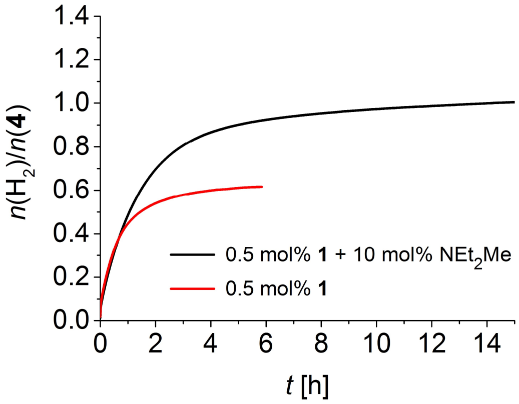

Figure S75. Volumetric curves of dehydropolymerisation of **4** using complex **1** in toluene and at T = 25 °C and [**4**] = 0.33 M.

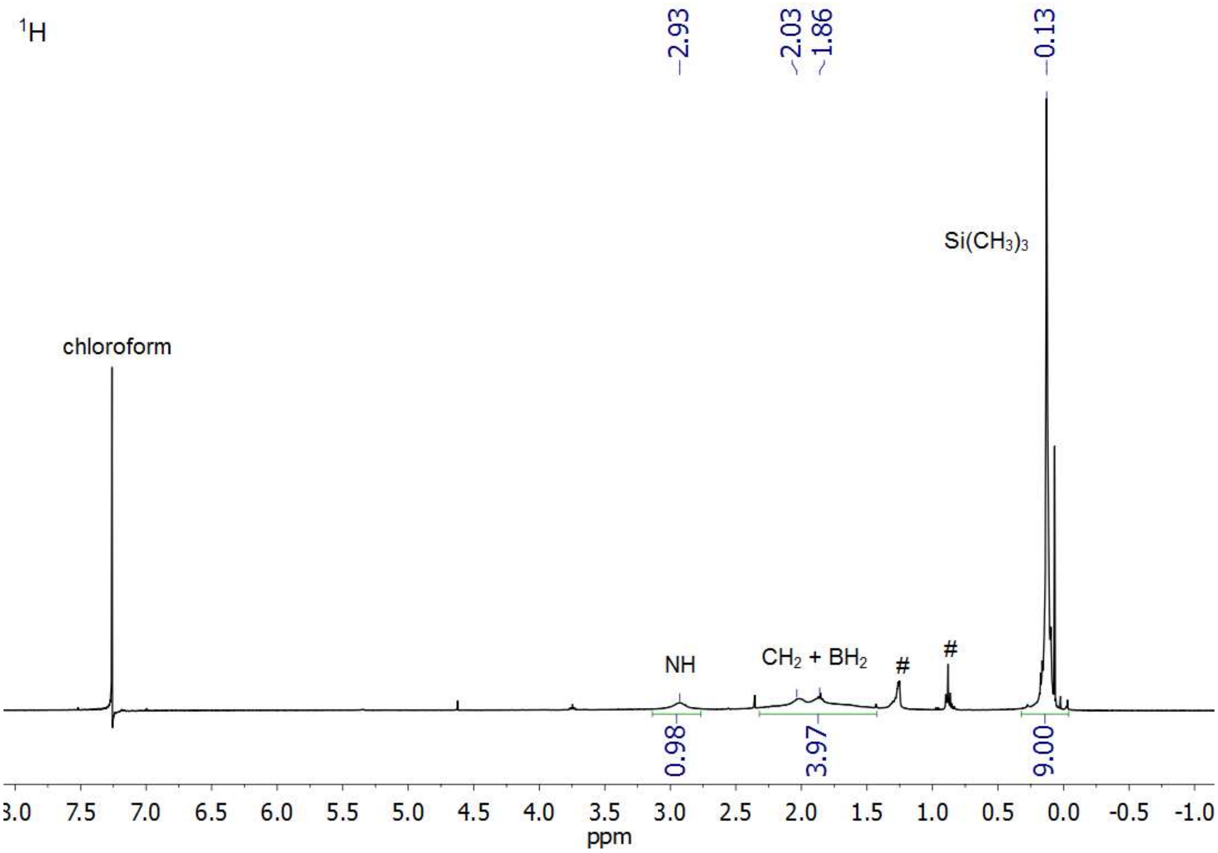

Figure S76. ^{1}H NMR spectrum (300 MHz, $CDCl_3$) of the isolated residue from dehydropolymerisation of **4** using complex **1** in toluene at T = 25 °C and [$H_3B{\cdot}NRH_2$] = 0.33 M. # = *n*-hexane. R = CH_2SiMe_3.

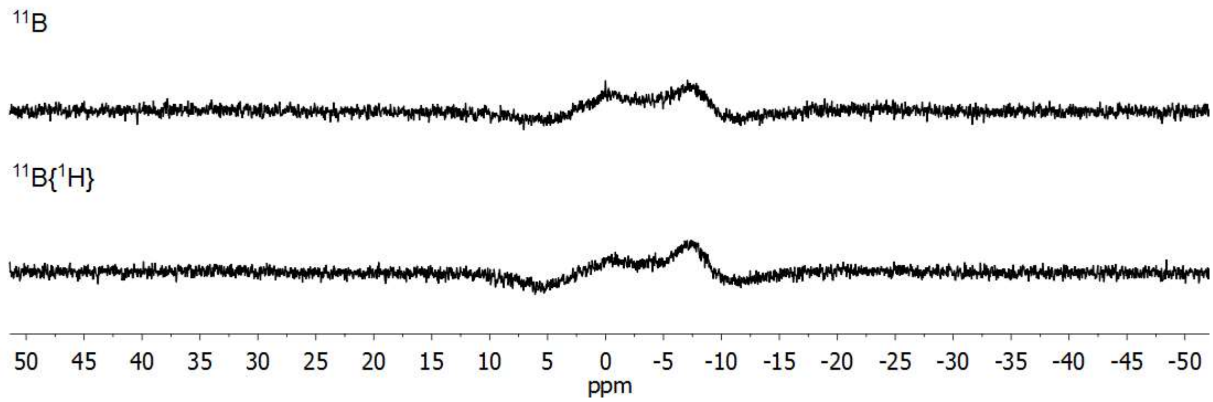

Figure S77. ^{11}B and $^{11}B\{^1H\}$ NMR spectra (96 MHz, $CDCl_3$) of the isolated residue from dehydropolymerisation of **4** using complex **1** in toluene at T = 25 °C and [$H_3B·NRH_2$] = 0.33 M.

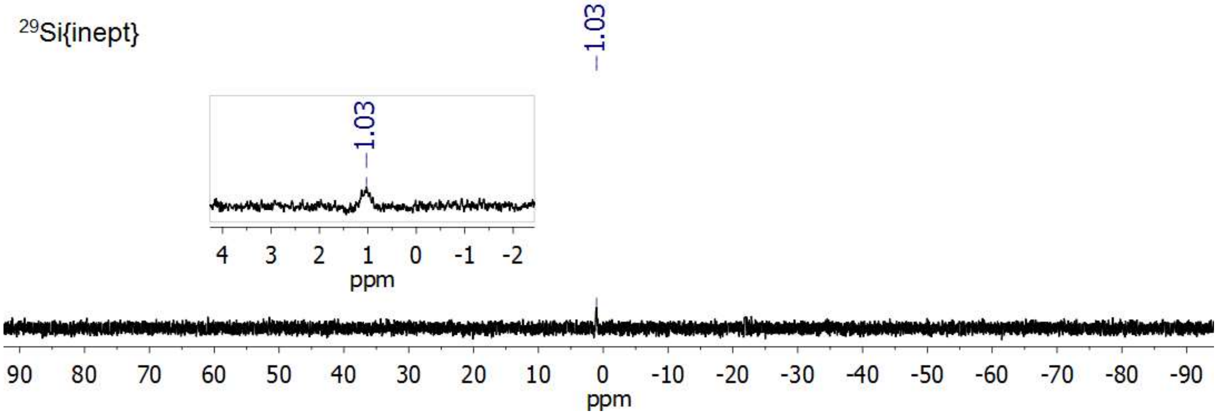

Figure S78. ^{29}Si\{inept\} NMR (79 MHz, $CDCl_3$) spectrum of the isolated residue from dehydropolymerisation of **4** using complex **1** in toluene at T = 25 °C and [$H_3B·NRH_2$] = 0.33 M.

Co-dehydropolymerisation of H_3BNMeH_2 and 4: $H_3B·NMeH_2$ (30 mg, 0.067 mmol), **4** (78 mg, 0.067 mmol) and the corresponding Fe catalyst were weighed in the glovebox and transferred to a three-necked reaction vessel. Then, amine borane containing dehydrogenation vessel was connected to the gas buret under Ar atmosphere. The gas buret was initialised and the amine borane adducts and Fe catalyst was dissolved in 4 mL of THF or toluene and data acquisition was started immediately. After completion of the dehydrogenation reaction, a gas sample was taken and analysed by GC-TCD and an aliquot was analysed by ^{11}B NMR spectroscopy.

The solution was concentrated in vacuum and acetonitrile was added, resulting in precipitation of plates, followed by filtration. The pale yellow solid was dried in vacuum overnight.

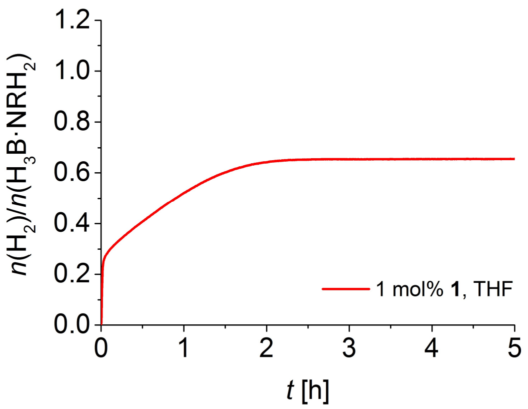

Figure S79. Volumetric curves of co-dehydropolymerisation of $H_3B{\cdot}NMeH_2$ and **4** (1:1) using complex **1** in THF and at T = 25 °C and $[H_3B{\cdot}NRH_2]$ = 0.33 M. R = Me and CH_2SiMe_3.

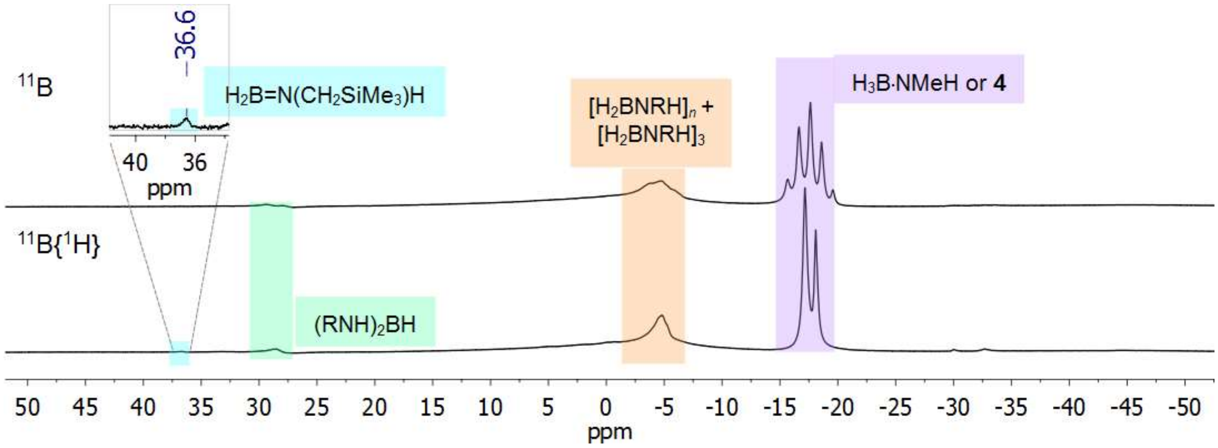

Figure S80. *In situ* ^{11}B and $^{11}B\{^1H\}$ NMR spectra (96 MHz, THF-d_8) after co-dehydropolymerisation of $H_3B{\cdot}NMeH_2$ and **4** (1:1) in THF. R = Me and CH_2SiMe_3.

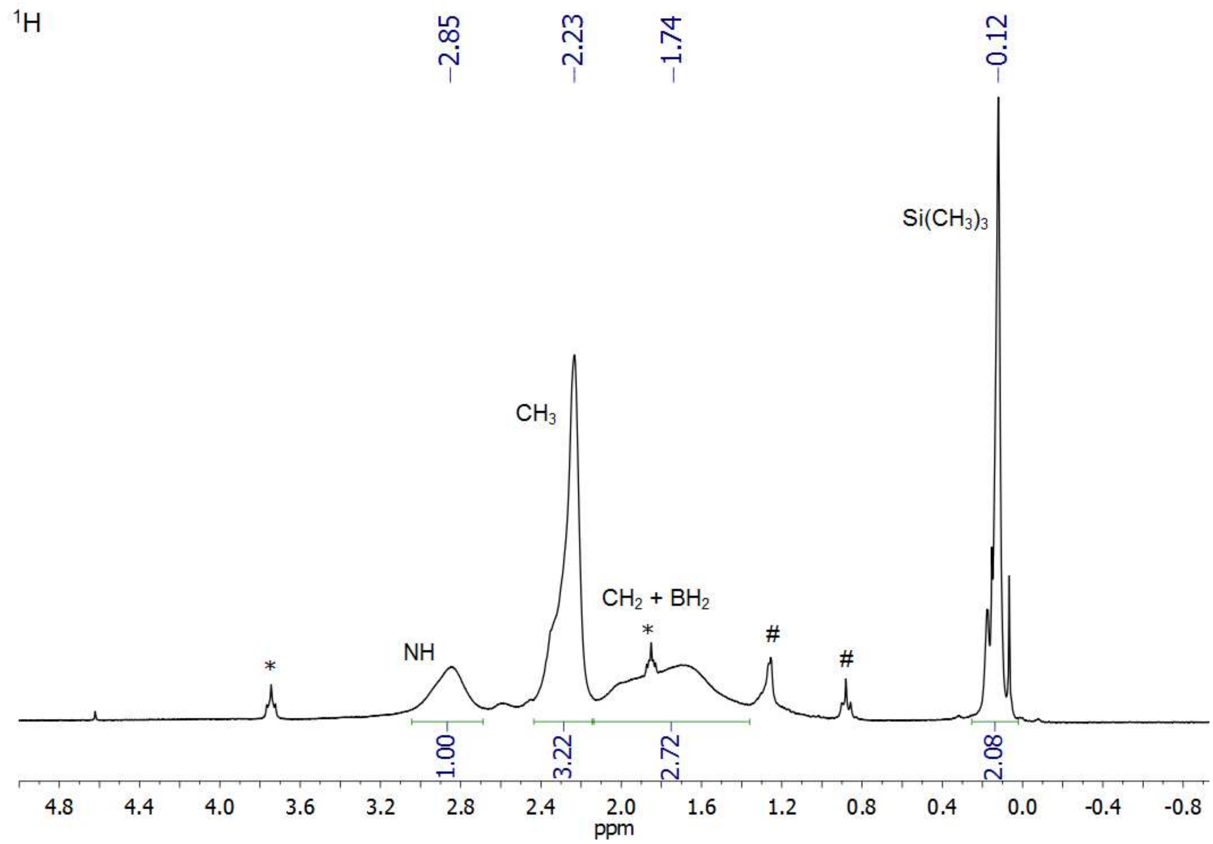

Figure S81. ^{1}H NMR spectrum (300 MHz, $CDCl_3$) of the residue from co-dehydropolymerisation of $H_3B{\cdot}NMeH_2$ and **4** using complex **1** in THF at T = 25 °C and $[H_3B{\cdot}NRH_2]$ = 0.33 M. # = *n*-hexane; * = THF. R = Me or CH_2SiMe_3.

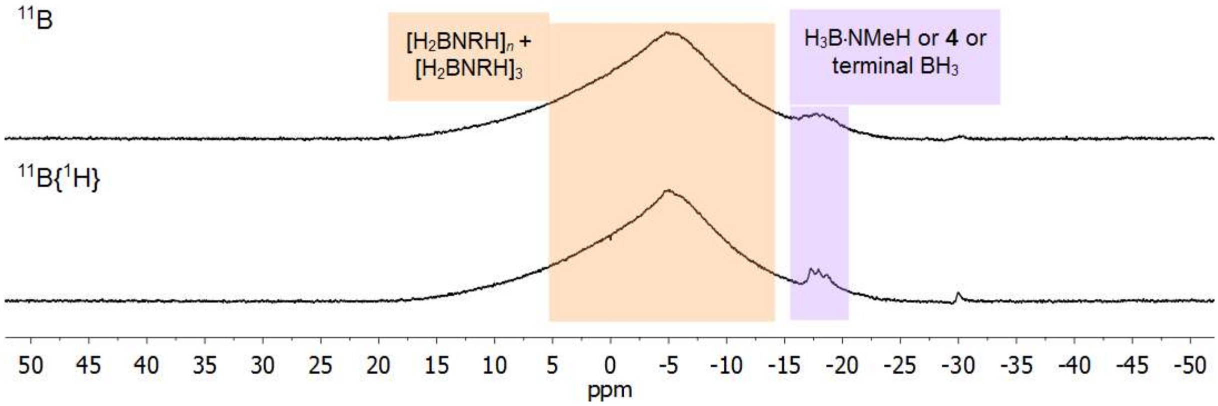

Figure S82. ^{11}B and ^{11}B{^{1}H} NMR spectra (96 MHz, $CDCl_3$) of the residue from co-dehydropolymerisation of $H_3B{\cdot}NMeH_2$ and **4** using complex **1** in THF at T = 25 °C and $[H_3B{\cdot}NRH_2]$ = 0.33 M. . R = Me or CH_2SiMe_3.

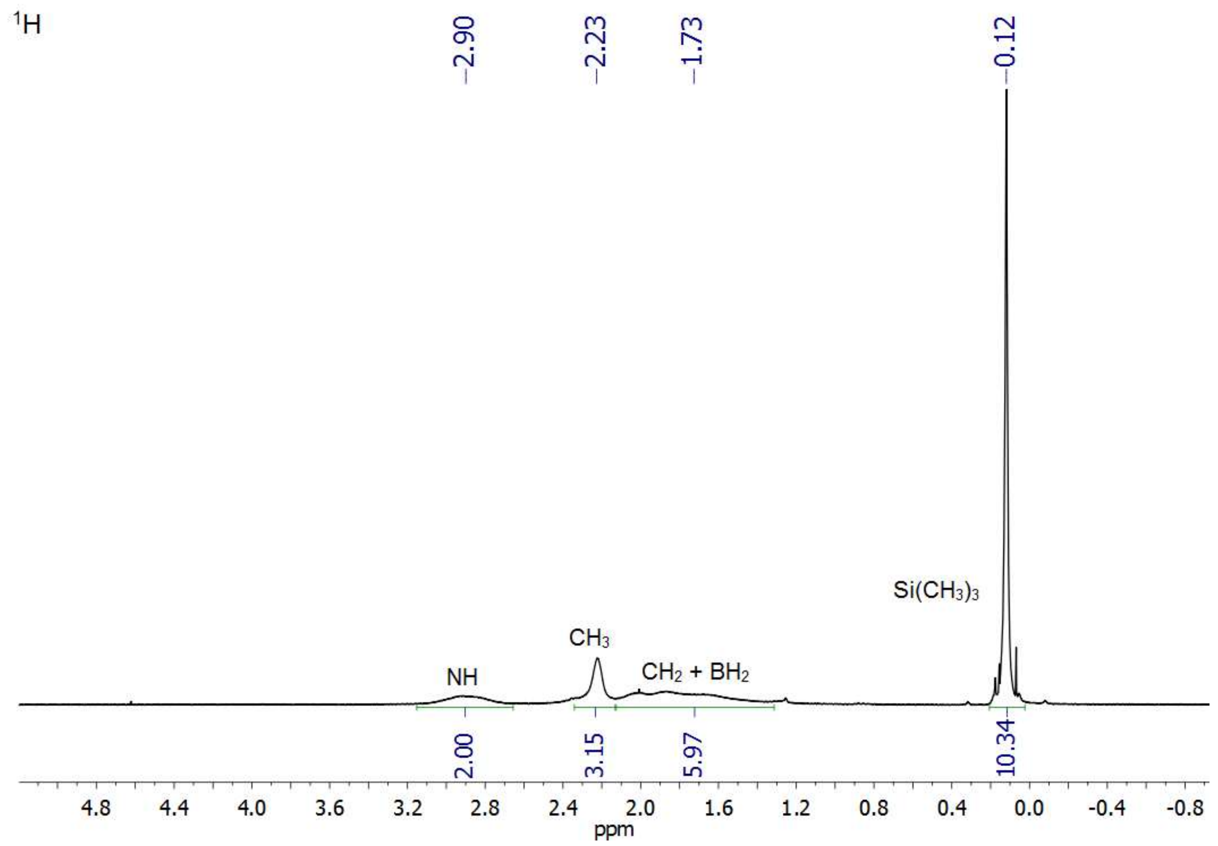

Figure S83. ^{1}H NMR spectrum (300 MHz, $CDCl_3$) of the copolymer from co-dehydropolymerisation of $H_3B{\cdot}NMeH_2$ and **4** using complex **1** in toluene at T = 25 °C and [$H_3B{\cdot}NRH_2$] = 0.33 M.

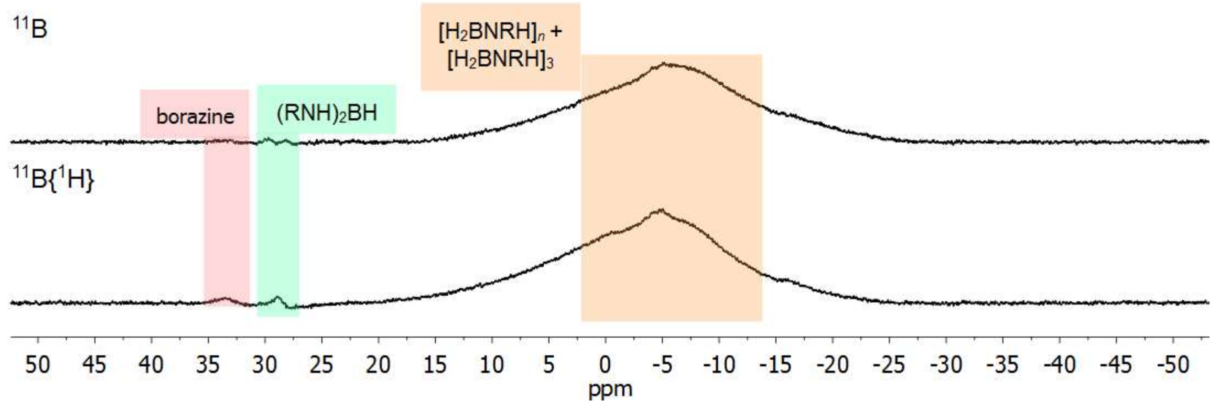

Figure S84. *In situ* ^{11}B and ^{11}B{^{1}H} NMR spectra (96 MHz, toluene-d_8) after co-dehydropolymerisation of $H_3B{\cdot}NMeH_2$ and **4** (1:1) in toluene. R = Me and CH_2SiMe_3.

References

[1] C. A. Jaska, K. Temple, A. J. Lough, I. Manners, *J. Am. Chem. Soc.* **2003**, *125*, 9424-9434.
[2] N. E. Stubbs, E. M. Leitao, C. H. Woodall, I. Manners, *Chem. Commun.* **2013**, *49*, 9098-9100.
[3] I. Koehne, T. J. Schmeier, E. A. Bielinski, C. J. Pan, P. O. Lagaditis, W. H. Bernskoetter, M. K. Takase, C. Würtele, N. Hazari, S. Schneider, *Inorg. Chem.* **2014**, *53*, 2133-2143.
[4] F. Anke, D. Han, M. Klahn, A. Spannenberg, T. Beweries, *Dalton Trans.* **2017**, *46*, 6843-6847.
[5] C. Bornschein, S. Werkmeister, B. Wendt, H. Jiao, E. Alberico, W. Baumann, H. Junge, K. Junge, M. Beller, *Nat. Commun.* **2014**, *5*, 4111.
[6] S. Werkmeister, K. Junge, B. Wendt, E. Alberico, H. Jiao, W. Baumann, H. Junge, F. Gallou, M. Beller, *Angew. Chem. Int. Ed.* **2014**, *53*, 8722-8726.
[7] I. Göttker-Schnetmann, P. S. White, M. Brookhart, *Organometallics* **2004**, *23*, 1766-1776.
[8] G. M. Sheldrick, *Acta Cryst.* **2008**, *A64*, 112.
[9] G. M. Sheldrick, *Acta Cryst.* **2015**, *C71*, 3-8.
[10] Diamond - Crystal and Molecular Structure Visualisation, Crystal Impact - Dr. H. Putz & Dr. K. Brandenburg GbR, Kreuzherrenstr. 102, 53227 Bonn, Germany, http://www.crystalimpact.com/diamond.
[11] M. Trose, M. Reiß, F. Reiß, F. Anke, A. Spannenberg, S. Boye, A. Lederer, P. Arndt, T. Beweries, *Dalton Trans.* **2018**, *47*, 12858-12862.
[12] T. Beweries, J. Thomas, M. Klahn, A. Schulz, D. Heller, U. Rosenthal, *ChemCatChem* **2011**, *3*, 1865-1868.
[13] Johannes G. De Vries, C. J. Elsevier, *The Handbook of Homogeneous Hydrogenation*, Wiley-VCH Verlag GmbH & KGaA, Weinheim, **2007**.
[14] Matthias Beller, Albert Renken, R. A. v. Santen, Wiley-VCH Verlag GmbH & KGaA, Weinheim, **2013**.
[15] *https://www.messen-nord.de/home.html*.
[16] E. Framery, M. Vaultier, *Heteroat. Chem.* **2000**, *11*, 218-225.
[17] J. Burés, *Angew. Chem. Int. Ed.* **2016**, *55*, 16084-16087.
[18] C. D. T. Nielsen, J. Burés, *Chem. Sci.* **2019**, *10*, 348-353.
[19] H. C. Johnson, A. P. Robertson, A. B. Chaplin, L. J. Sewell, A. L. Thompson, M. F. Haddow, I. Manners, A. S. Weller, *J. Am. Chem. Soc.* **2011**, *133*, 11076-11079.
[20] A. L. Colebatch, B. W. Hawkey Gilder, G. R. Whittell, N. L. Oldroyd, I. Manners, A. S. Weller, *Chem. Eur. J.* **2018**, *24*, 5450-5455.
[21] A. Friedrich, M. Drees, S. Schneider, *Chem. Eur. J.* **2009**, *15*, 10339-10342.
[22] A. Glüer, M. Förster, V. R. Celinski, J. Schmedt auf der Günne, M. C. Holthausen, S. Schneider, *ACS Catal.* **2015**, *5*, 7214-7217.
[23] O. J. Metters, A. M. Chapman, A. P. M. Robertson, C. H. Woodall, P. J. Gates, D. F. Wass, I. Manners, *Chem. Commun.* **2014**, *50*, 12146-12149.
[24] C. K. Narula, J. F. Janik, E. N. Duesler, R. T. Paine, R. Schaeffer, *Inorg. Chem.* **1986**, *25*, 3346-3349.
[25] D. F. Gaines, R. Schaeffer, *J. Am. Chem. Soc.* **1964**, *86*, 1505-1507.
